# Supplementary material for: MetaRibo-Seq measures translation in microbiomes
Source: Nat Commun. 2020 Jun 29;11:3268. doi: 10.1038/s41467-020-17081-z (PMC7324362; doi:10.1038/s41467-020-17081-z)
Supplement: Supplementary file 1 — Supplementary Information [file 41467_2020_17081_MOESM1_ESM.pdf]

Supplementary information

MetaRibo-Seq measures translation in microbiomes

Brayon J. Fremin[1], Hila Sberro[1,2], and Ami S. Bhatt[1,2*]
[1]Department of Genetics, Stanford University, CA, 94305, USA
[2]Department of Medicine (Hematology & Blood and Marrow Transplantation), Stanford University, CA, 94305, USA
*Correspondence: asbhatt@stanford.edu

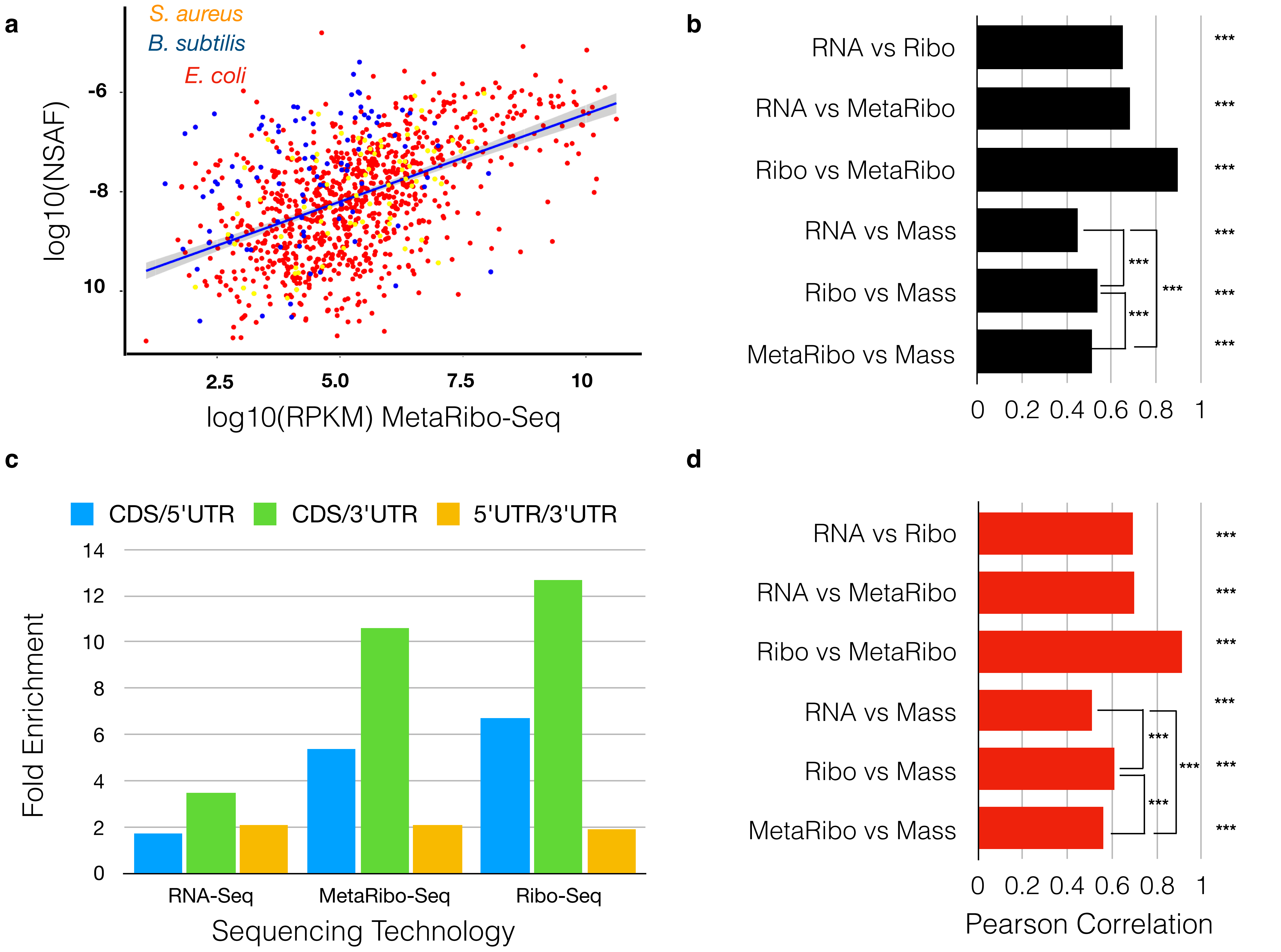
a
S. aureus
B. subtilis
E. coli
log10(NSAF)
-6
-8
10
2.5
5.0
7.5
10
log10(RPKM) MetaRibo-Seq
b
RNA vs Ribo
RNA vs MetaRibo
Ribo vs MetaRibo
RNA vs Mass
Ribo vs Mass
MetaRibo vs Mass
***
0 0.2 0.4 0.6 0.8 1
c
CDS/5'UTR
CDS/3'UTR
5'UTR/3'UTR
Fold Enrichment
14
12
10
8
6
4
2
0
RNA-Seq
MetaRibo-Seq
Ribo-Seq
Sequencing Technology
d
RNA vs Ribo
RNA vs MetaRibo
Ribo vs MetaRibo
RNA vs Mass
Ribo vs Mass
MetaRibo vs Mass
***
0 0.2 0.4 0.6 0.8 1
Pearson Correlation

Supplementary Fig. 1

MetaRibo-Seq correlates to protein levels in an aerobic mock community.

(a) Scatterplot of MetaRibo-Seq RPKM and metaproteomics NSAF (Normalized Spectral Abundance Factor) log-scaled (n = 1055 proteins) in the aerobic mock community. Replicates reads were combined and treated as a single sample for this visualization.

(b) Pearson correlations for pairwise comparisons across technologies to metaproteomics in the aerobic mock community. These correlations to metaproteomics (n = 1055) include RNA-Seq (Pearson's r = 0.45, p value < 2^{-16}), Ribo-Seq (Pearson's r = 0.54, p value < 2^{-16}), and MetaRibo-Seq (Pearson's r = 0.51, p value < 2^{-16}). MetaRibo-Seq is a significantly better predictor of protein abundance than transcriptomics with Zou's[54] 95% confidence interval between -0.1016 and -0.0187 (significance indicated as ***). Ribo-Seq is a significantly better predictor of protein abundance than transcriptomics with Zou's[54] 95 % confidence interval between -0.1330 and -0.0474 (significance indicated as ***).

(c) Histogram showing the fold enrichment of signal across coding regions flanked by UTRs (n = 2263) compared to signal across known UTR regions for RNA-Seq, MetaRibo-Seq, and Ribo-Seq in *E. coli*.

(d) Pearson correlations for pairwise comparisons across technologies to metaproteomics in *E. coli* in the aerobic mock community. These correlations to metaproteomics (n = 917) include RNA-Seq (Pearson's r = 0.51, p value < 2^{-16}), Ribo-Seq (Pearson's r = 0.61, p value < 2^{-16}), and MetaRibo-Seq (Pearson's r = 0.56, p value < 2^{-16}). MetaRibo-Seq is a significantly better predictor of protein abundance than transcriptomics with Zou's[54] 95% confidence interval between -0.0915 and -0.0089 (significance indicated as ***). Ribo-Seq is a significantly better predictor of protein abundance than transcriptomics with Zou's[54] 95 % confidence interval between -0.1415 and -0.0593 (significance indicated as ***).

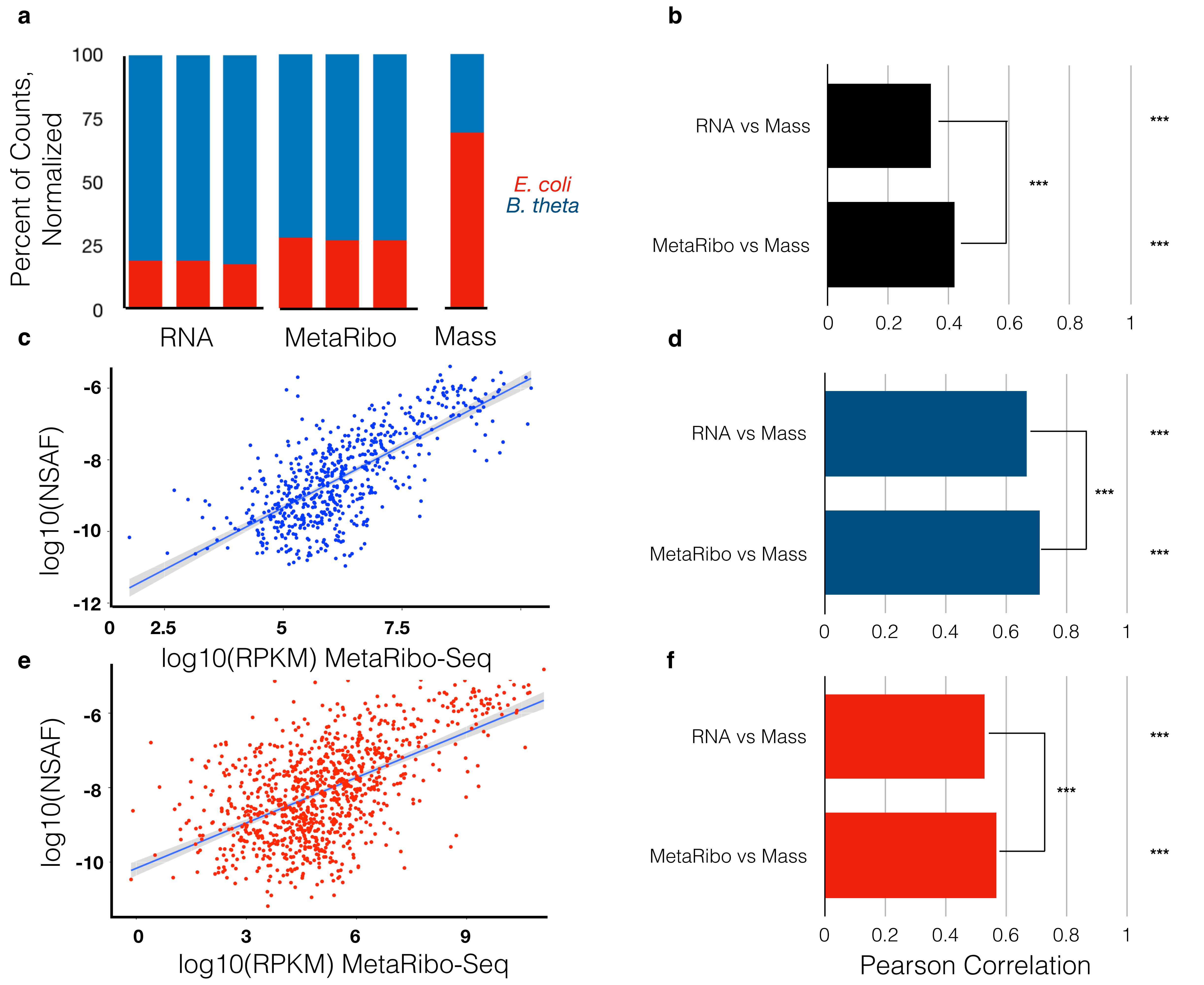
a
Percent of Counts, Normalized
100
75
50
25
0
RNA
MetaRibo
Mass
E. coli
B. theta
b
RNA vs Mass
MetaRibo vs Mass
***
***
***
0
0.2
0.4
0.6
0.8
1
c
log10(NSAF)
-6
-8
-10
-12
0
2.5
5
7.5
log10(RPKM) MetaRibo-Seq
d
RNA vs Mass
MetaRibo vs Mass
***
***
***
0
0.2
0.4
0.6
0.8
1
e
log10(NSAF)
-6
-8
-10
0
3
6
9
log10(RPKM) MetaRibo-Seq
f
RNA vs Mass
MetaRibo vs Mass
***
***
***
0
0.2
0.4
0.6
0.8
1
Pearson Correlation

Supplementary Fig. 2

MetaRibo-Seq correlates to protein levels in an anaerobic mock community.

(a) Proportion of count data from transcriptomics, MetaRibo-Seq, and proteomics corresponding to *E. coli* (red) and *B. thetaiotaomicron* (blue) in a two member mock mixture.

(b) Pearson correlations for pairwise comparisons across technologies in the mixture. These correlations to metaproteomics (n = 1,590 proteins) include RNA-Seq (Pearson's r = 0.34, p value < 2^{-16}) and MetaRibo-Seq (Pearson's r = 0.42, p value < 2^{-16}). MetaRibo-Seq is a significantly better predictor of protein abundance than transcriptomics with Zou's[54] 95% confidence interval between -0.0732 and -0.0327 (significance indicated as ***).

(c) Scatterplot of MetaRibo-Seq RPKM and metaproteomics NSAF log-scaled (n=596 proteins) in *B. thetaiotaomicron*. Replicates reads were combined and treated as a single sample for this visualization.

(d) Pearson correlations for pairwise comparisons across technologies in the *B. thetaiotaomicron*. These correlations to metaproteomics (n = 596) include RNA-Seq (Pearson's r = 0.67, p value < 2^{-16}) and MetaRibo-Seq (Pearson's r = 0.71, p value < 2^{-16}). MetaRibo-Seq is a significantly better predictor of protein abundance than transcriptomics with Zou's[54] 95% confidence interval between -0.0602 and -0.0219 (significance indicated as ***).

(e) Scatterplot of MetaRibo-Seq RPKM and metaproteomics NSAF log-scaled (n = 994 proteins) in *E. coli*. Replicates reads were combined and treated as a single sample for this visualization.

(f) Pearson correlations for pairwise comparisons across technologies in *E. coli*. These correlations to metaproteomics (n = 994) include RNA-Seq (Pearson's r = 0.53, p value < 2^{-16}) and MetaRibo-Seq (Pearson's r = 0.57, p value < 2^{-16}). MetaRibo-Seq is a significantly better predictor of protein abundance than transcriptomics with Zou's[54] 95% confidence interval between -0.0572 and -0.0237 (significance indicated as ***).

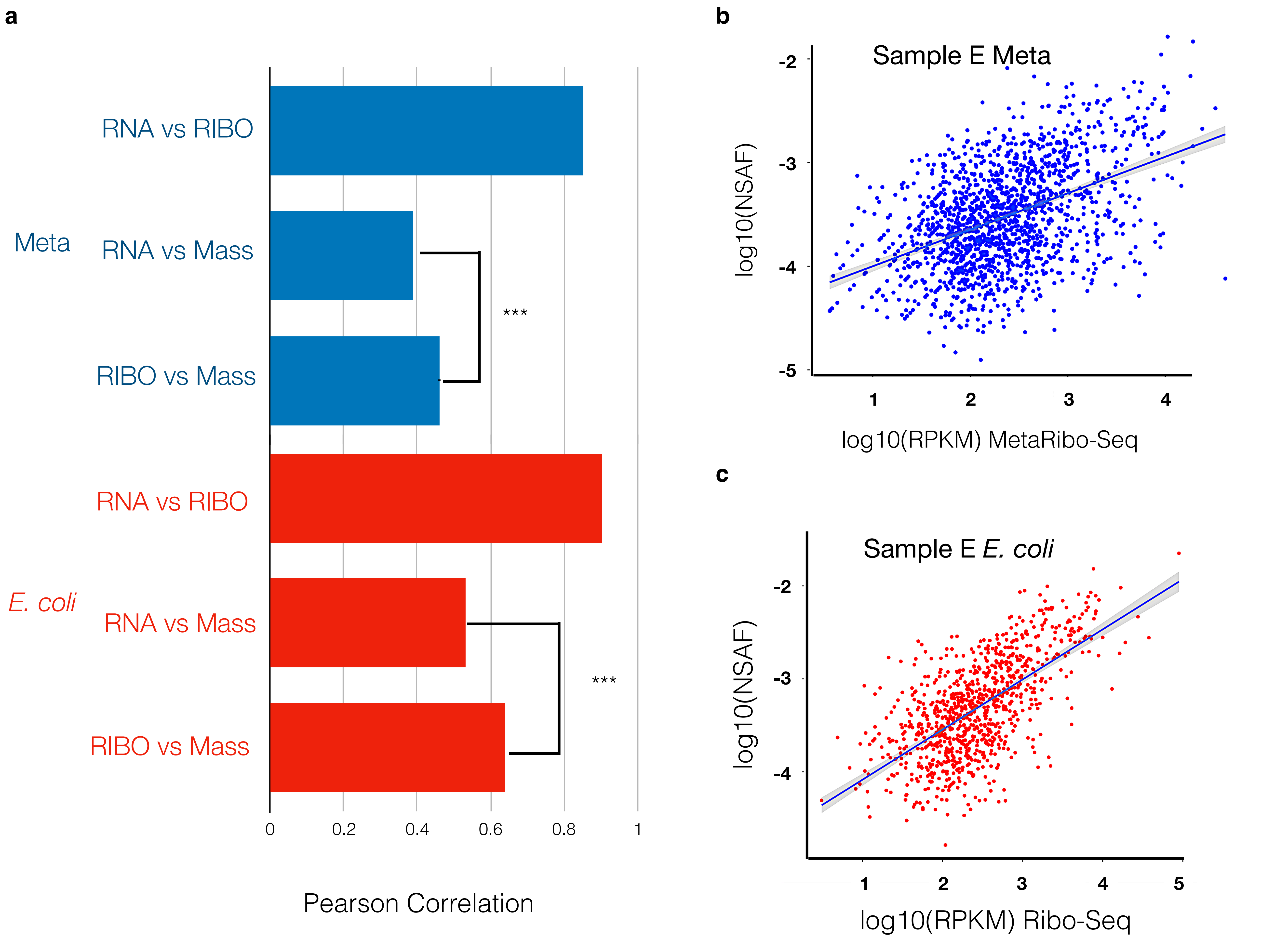

a
Meta
RNA vs RIBO
RNA vs Mass
RIBO vs Mass
***
E. coli
RNA vs RIBO
RNA vs Mass
RIBO vs Mass
***
0
0.2
0.4
0.6
0.8
1
Pearson Correlation
b
Sample E Meta
log10(NSAF)
-2
-3
-4
-5
1
2
3
4
log10(RPKM) MetaRibo-Seq
c
Sample E E. coli
log10(NSAF)
-2
-3
-4
1
2
3
4
5
log10(RPKM) Ribo-Seq

Supplementary Fig. 3

MetaRibo-Seq correlates to protein levels in a low diversity fecal sample.

(a) Pearson correlations for pairwise comparisons across technologies. Blue bars indicate Pearson correlations pertaining to all detected proteins (n = 1,503) in Sample E. Pearson's r comparing metatranscriptomics vs. MetaRibo-Seq, metatranscriptomics vs. metaproteomics, and MetaRibo-Seq vs. metaproteomics are 0.85, 0.39, and 0.46, respectively. All are significant (p value < 2^{-16}). MetaRibo-Seq is a significantly better predictor of protein abundance with Zou's[68] 95 % confidence interval between -0.0917 to -0.0487. Red bars indicate Pearson correlations pertaining only to *E. coli* in Sample E (n = 928 proteins). The correlations between metatranscriptomics vs. MetaRibo-Seq, metatranscriptomics vs. metaproteomics, and MetaRibo-Seq vs. metaproteomics are 0.90, 0.53, and 0.64, respectively. All are significant (p value < 2^{-16}). MetaRibo-Seq is a significantly better predictor of protein abundance with Zou's[54] 95 % confidence interval between -0.1352 and -0.0868 (significance indicated as ***).

(b) Scatterplot of MetaRibo-Seq RPKM and metaproteomics NSAF log-scaled (n = 1,503 proteins) detected by metaproteomics in Sample E. Replicates reads were combined and treated as a single sample for this visualization.

(c) Scatterplot of MetaRibo-Seq RPKM and metaproteomics NSAF log-scaled (n = 928 proteins) from*E. coli* in Sample E. Replicates reads were combined and treated as a single sample for this visualization.

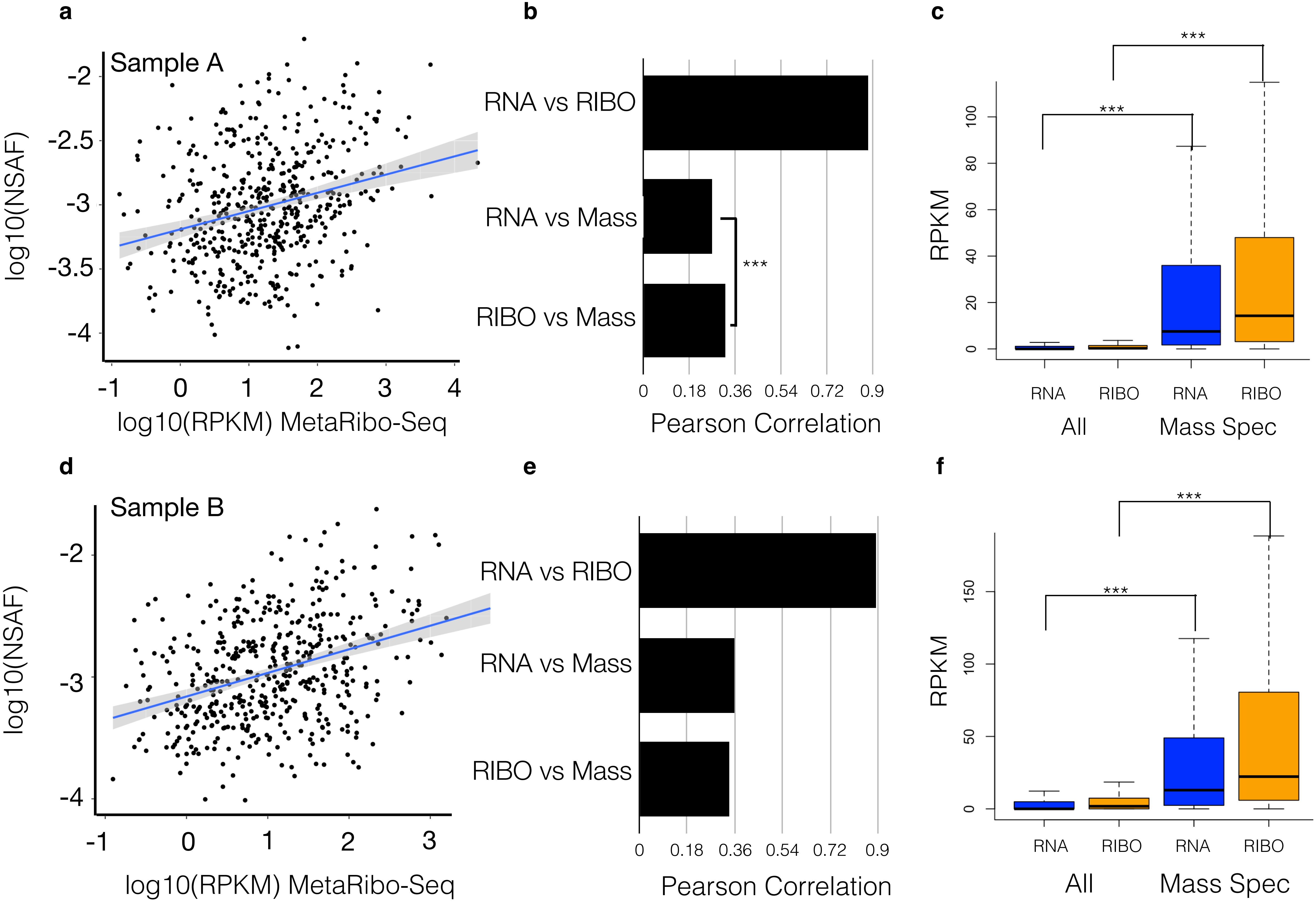

a
Sample A
log10(NSAF)
log10(RPKM) MetaRibo-Seq
b
RNA vs RIBO
RNA vs Mass
RIBO vs Mass
***
Pearson Correlation
0
0.18
0.36
0.54
0.72
0.9
c
RPKM
***
***
RNA
RIBO
RNA
RIBO
All
Mass Spec
d
Sample B
log10(NSAF)
log10(RPKM) MetaRibo-Seq
e
RNA vs RIBO
RNA vs Mass
RIBO vs Mass
Pearson Correlation
0
0.18
0.36
0.54
0.72
0.9
f
RPKM
***
***
RNA
RIBO
RNA
RIBO
All
Mass Spec

Supplementary Fig. 4

MetaRibo-Seq signal correlates to metaproteomics and is enriched for these proteins.

(a) For Sample A, scatterplot of MetaRibo-Seq RPKM and metaproteomics NSAF both log-scaled (n=497 proteins). Replicates reads were combined and treated as a single sample for this visualization.

(b) For Sample A, pairwise Pearson correlations between log-scaled metatranscriptomics RPKM, MetaRibo-Seq RPKM, and metaproteomics NSAF (n=497). Pearson's r are 0.88, 0.26, and 0.32 for metatranscriptomics vs. MetaRibo-Seq, metatranscriptomics vs. metaproteomics, and MetaRibo-Seq vs. metaproteomics, respectively. All are significant (p value $< 2^{-16}$). MetaRibo-Seq is a significantly better predictor of protein abundance than metatranscriptomics for these proteins in Sample A with a Zou's[54] 95 % confidence interval between -0.1322 and -0.0480 (significance indicated as ***)..

(c) For Sample A, metatranscriptomics and MetaRibo-Seq RPKM for all predicted genes (n= 223,630) compared to those detected by metaproteomics (n=497). For all predicted genes, median, mean, maximum, and minimum RNA-Seq RPKM values are 0, 10.95, 1102090, and 0, respectively. For all predicted genes, median, mean, maximum, and minimum MetaRibo-Seq RPKM values are 0, 10.54, 148426, and 0, respectively. For those genes detected by metaproteomics, median, mean, maximum, and minimum RNA-Seq RPKM values are 2.98, 22.30, 1031.33, and 0, respectively. For those genes detected by metaproteomics, median, mean, maximum, and minimum MetaRibo-Seq RPKM values are 8.41,45.52, 2417.14, and 0, respectively. Both metatranscriptomic and MetaRibo-Seq signal for proteomically-detected proteins are significantly enriched (two-sided Kolmogorov-Smirnov test p value $< 2^{-16}$) compared to all genes.

(d) For Sample B, scatterplot of MetaRibo-Seq RPKM and metaproteomics NSAF both log10-scaled (n = 480 proteins).Replicates reads were combined and treated as a single sample for this visualization.

(e) For Sample B, pairwise Pearson correlations between log-scaled metatranscriptomics RPKM, MetaRibo-Seq RPKM, and metaproteomics NSAF (n=480). Pearson's r are 0.89, 0.36, and 0.34 for metatranscriptomics vs. MetaRibo-Seq, metatranscriptomics vs. metaproteomics, and MetaRibo-Seq vs. metaproteomics, respectively. All are significant (p value $< 2^{-16}$).

(f) For Sample B, metatranscriptomics and MetaRibo-Seq RPKM for all predicted genes (n= 196,683) compared to those detected by metaproteomics (n=480). For all predicted genes, median, mean, maximum, and minimum RNA-Seq RPKM values are 0, 11.13, 82470.7, and 0, respectively. For all predicted genes, median, mean, maximum, and minimum MetaRibo-Seq RPKM values are 0.14, 11.28, 94225, and 0, respectively. For those genes detected by metaproteomics, median, mean, maximum, and minimum RNA-Seq RPKM values are 10.45, 47.6, 1527.54, and 0, respectively. For those genes detected by metaproteomics, median, mean, maximum, and minimum MetaRibo-Seq RPKM values are 15.48, 75.11, 3335.91, and 0, respectively. Both metatranscriptomic and MetaRibo-Seq signal for proteomically-detected proteins are significantly enriched (two-sided Kolmogorov-Smirnov test p value $< 2^{-16}$) compared to all genes.

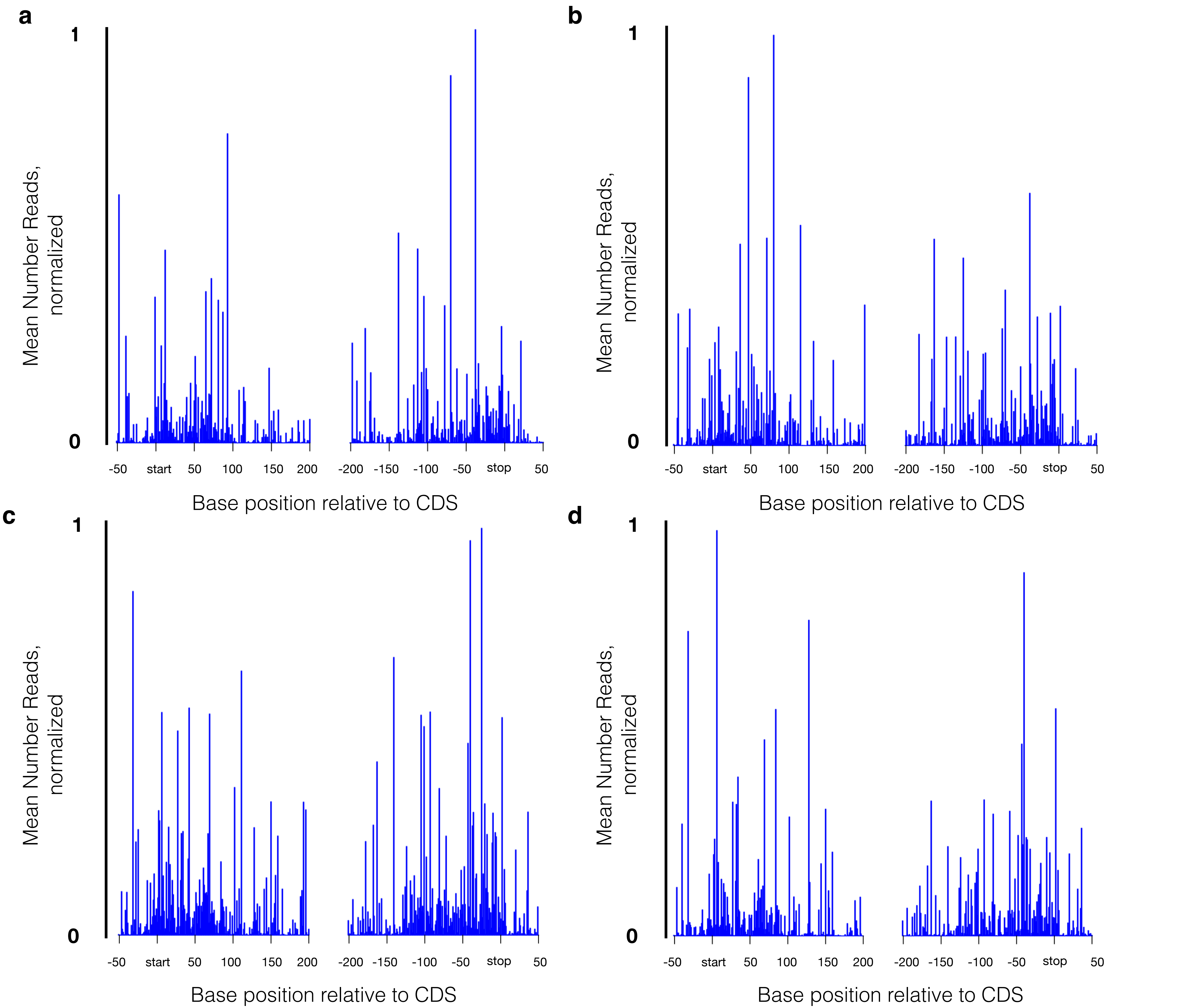
a
b
c
d
1
0
Mean Number Reads, normalized
-50
start
50
100
150
200
-200
-150
-100
-50
stop
50
Base position relative to CDS

Supplementary Fig. 5

RNA-Seq signal in fecal samples.

(a-d) Average RNA-Seq signal across genes and flanking regions for Sample A, B, C, and D, respectively. Every predicted open reading frame containing at least 10 reads is included in the analysis**.** Replicates reads are combined and treated as a single sample for this visualization.

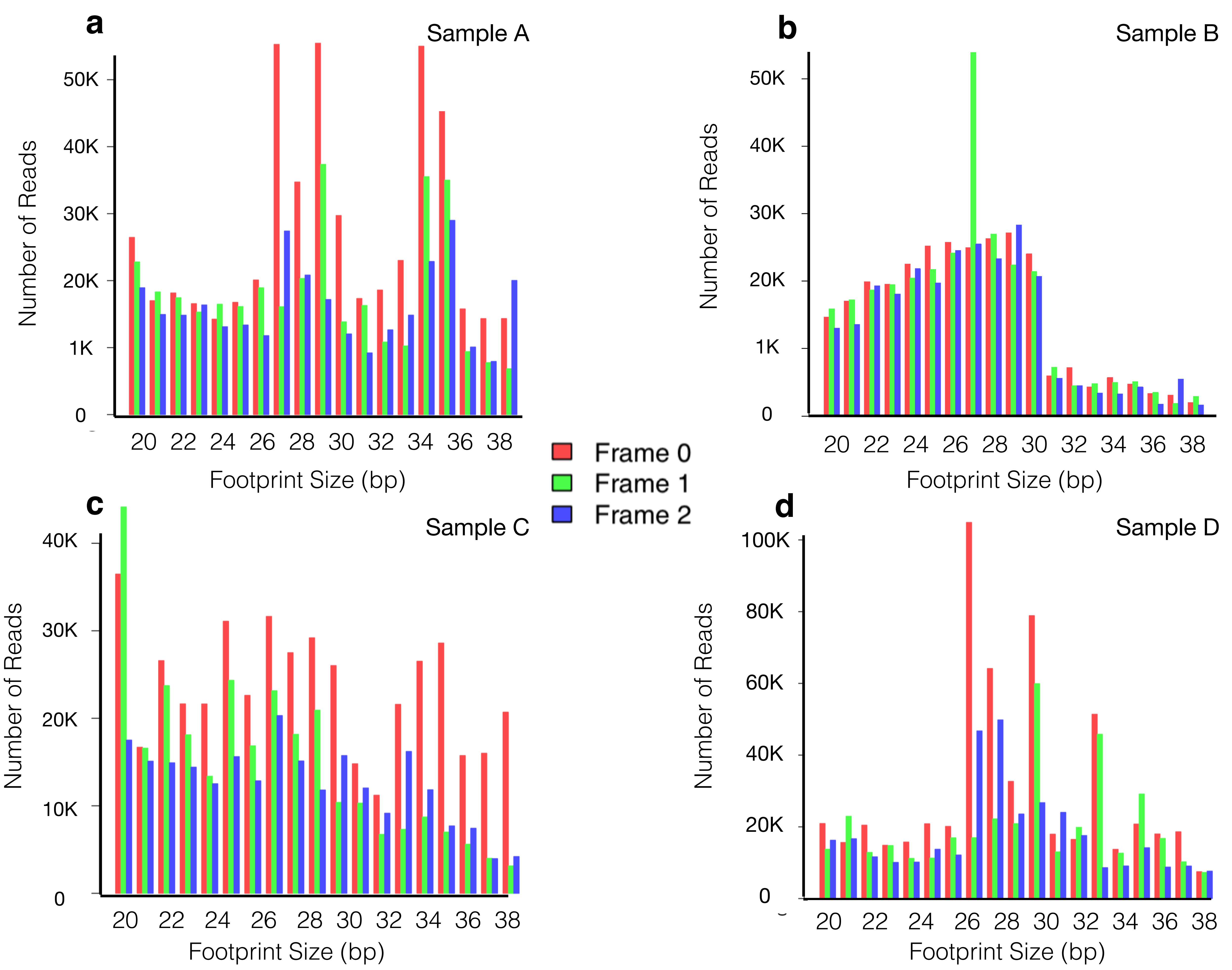

a
Sample A
b
Sample B
c
Sample C
d
Sample D
Number of Reads
Footprint Size (bp)
50K
40K
30K
20K
1K
0
100K
80K
60K
10K
20 22 24 26 28 30 32 34 36 38
Frame 0
Frame 1
Frame 2

Supplementary Fig. 6

MetaRibo-Seq demonstrates some weak signs of overall codon-resolution.

(a-d) Triplet periodicity across footprint lengths for Sample A, B, C, and D, respectively. Colors indicate which frame a read falls within.

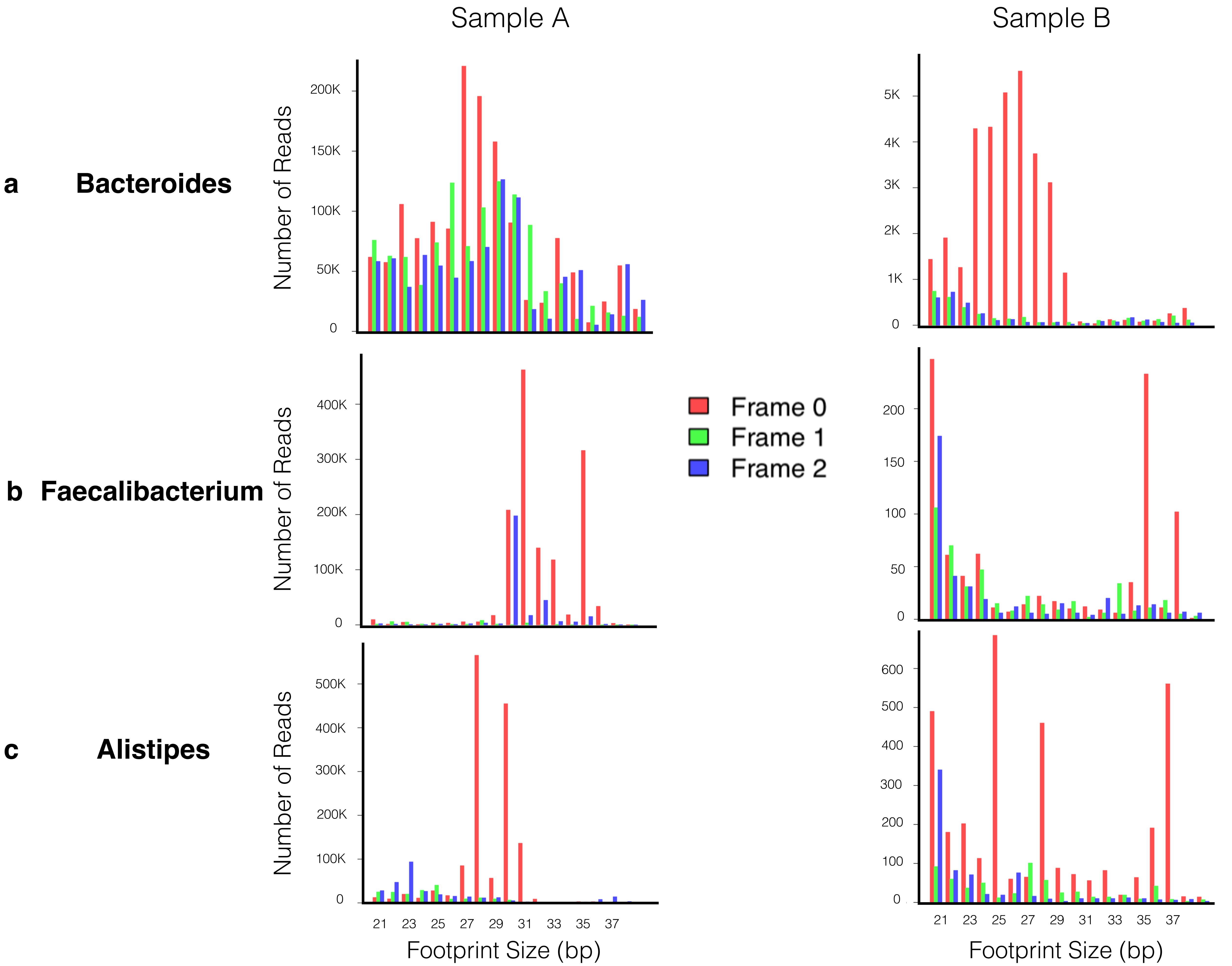

Sample A
Sample B
a Bacteroides
b Faecalibacterium
c Alistipes
Number of Reads
Frame 0
Frame 1
Frame 2
200K
150K
100K
50K
0
5K
4K
3K
2K
1K
400K
300K
200K
100K
200
150
100
50
500K
600
500
400
300
21
23
25
27
29
31
33
35
37
Footprint Size (bp)

Supplementary Fig. 7

MetaRibo-Seq demonstrates stronger codon resolution in taxa-specific analyses.

(a) All contigs assigned to the genera Bacteroides are considered from Sample A and B, respectively. Only these contigs are considered in triplet periodicity analyses.

(b and c) The same triplet periodicity analysis for Faecalibacterium and Alistipes, respectively.

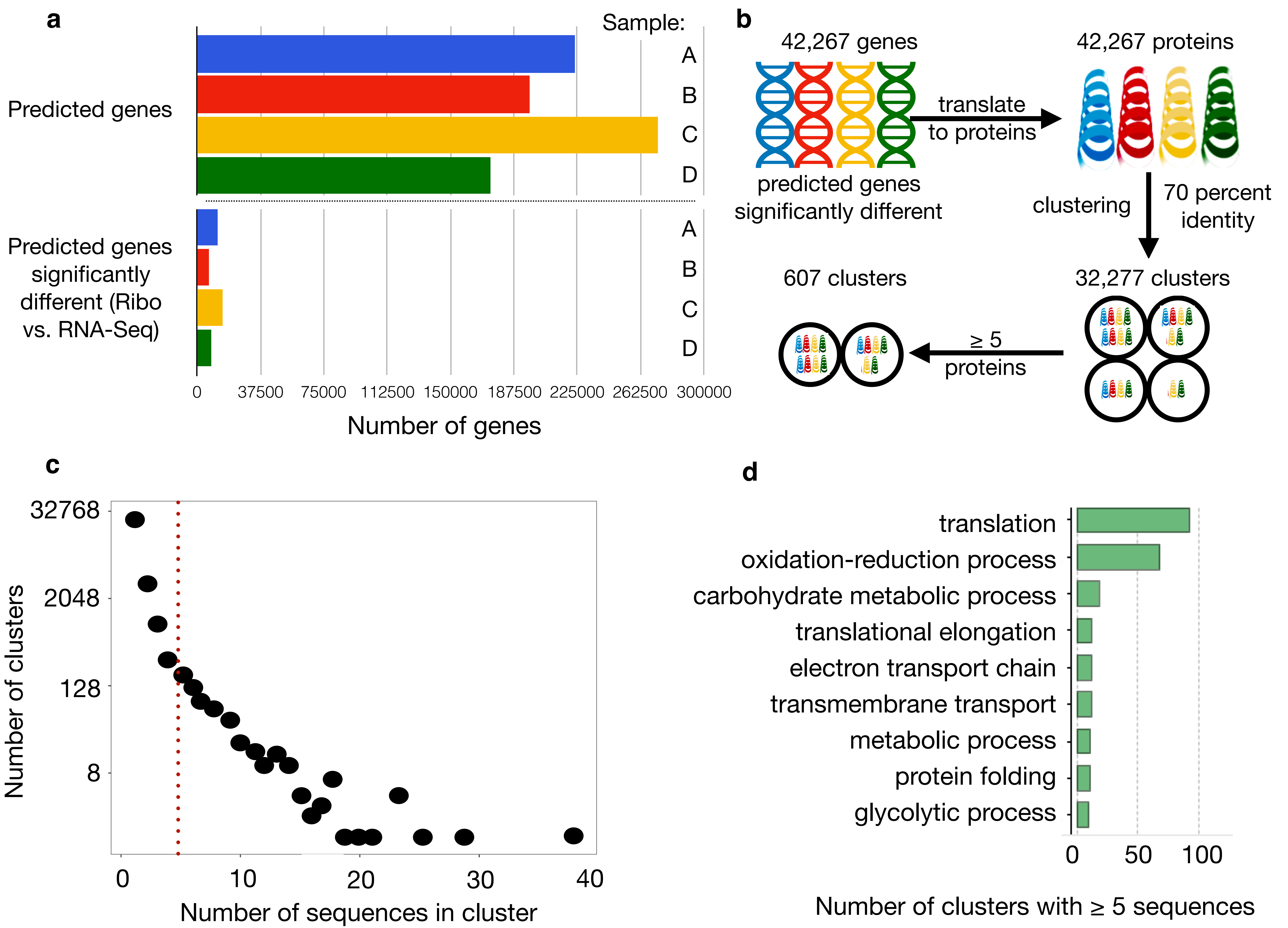
a
Sample:
Predicted genes
Predicted genes significantly different (Ribo vs. RNA-Seq)
A
B
C
D
0 37500 75000 112500 150000 187500 225000 262500 300000
Number of genes
b
42,267 genes
predicted genes significantly different
translate to proteins
42,267 proteins
clustering
70 percent identity
32,277 clusters
≥ 5 proteins
607 clusters
c
Number of clusters
32768
2048
128
8
0 10 20 30 40
Number of sequences in cluster
d
translation
oxidation-reduction process
carbohydrate metabolic process
translational elongation
electron transport chain
transmembrane transport
metabolic process
protein folding
glycolytic process
0 50 100
Number of clusters with ≥ 5 sequences

Supplementary Fig. 8

Genes are reproducibly translated at different levels than transcribed.

(a) For Samples, A, B, C, and D, we show the number of total gene predictions via prokka[36]. We performed differential relative abundance analysis using DESeq2[49] on each of these samples individually, comparing metatranscriptomics to MetaRibo-Seq. We show the number of those genes identified as translated differently than transcribed (absolute value(log2 fold change) > 1 and FDR < 0.05). We predict 223630, 196683, 272895, and 173624 genes from Sample A, B, C, and D, respectively. Among these, 11872, 6580, 15188, and 8647, respectively, are translated at significantly different levels than transcribed.

(b) Genes translated at different levels than transcribed are converted, *in silico*, from nucleic acid sequences to protein sequences and are then clustered at 70 percent amino acid identity.

(c) The number of clusters with specific numbers of sequences combined across Samples A, B, C, and D.

(d) If the combined cluster contained at least 5 sequences, this gene is considered reproducibly translated differently than transcribed. 607 clusters met this requirement. For these clusters, the representative sequence (see Methods) is selected to represent the entire cluster. These representatives were input into Blast2GO to assign GO terms based on protein sequence.

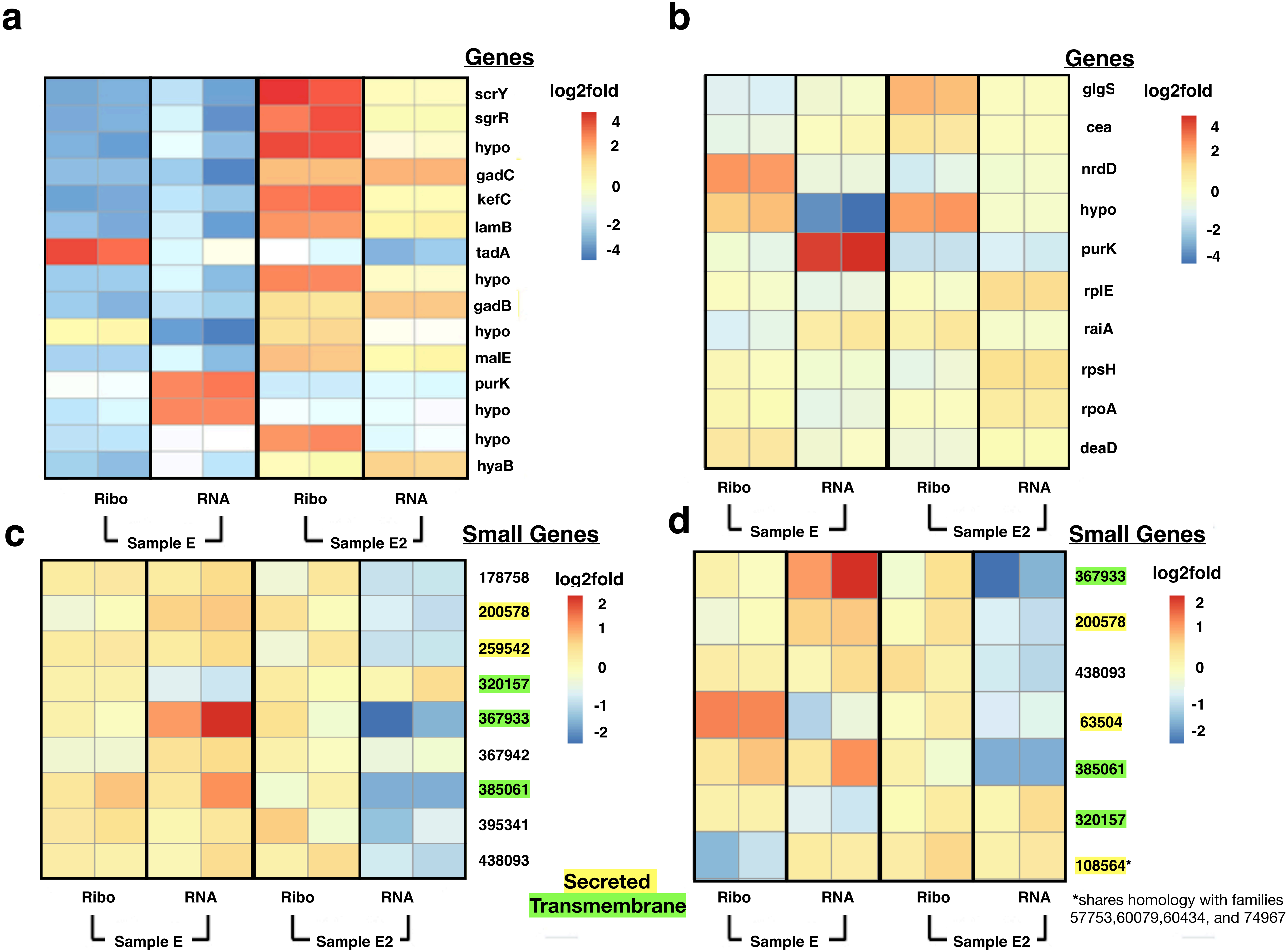
a
Genes
scrY
sgrR
hypo
gadC
kefC
lamB
tadA
hypo
gadB
hypo
malE
purK
hypo
hypo
hyaB
log2fold
4
2
0
-2
-4
Ribo
RNA
Ribo
RNA
Sample E
Sample E2
b
Genes
glgS
cea
nrdD
hypo
purK
rplE
raiA
rpsH
rpoA
deaD
log2fold
4
2
0
-2
-4
Ribo
RNA
Ribo
RNA
Sample E
Sample E2
c
Small Genes
178758
200578
259542
320157
367933
367942
385061
395341
438093
log2fold
2
1
0
-1
-2
Ribo
RNA
Ribo
RNA
Sample E
Sample E2
Secreted
Transmembrane
d
Small Genes
367933
200578
438093
63504
385061
320157
108564*
log2fold
2
1
0
-1
-2
Ribo
RNA
Ribo
RNA
Sample E
Sample E2
*shares homology with families 57753,60079,60434, and 74967

Supplementary Fig. 9

Translational regulation in fecal samples changes over time.

(a) Heatmap of the top 15 most variable genes in Ribo-Seq and small transcriptomics between time points in Sample E. Sample E2 was obtained 6 days after Sample E.

(b) Heatmap of the top 10 most significantly translationally regulated genes between time points in Sample E. Specifically, these are the 10 genes that are most significantly regulated at the translational level, controlling for transcriptional changes.

(c) Heatmap of all small protein families that were significantly different over time in comparing either Ribo-Seq or RNA-Seq.

(d) Heatmap of all translationally regulated small protein families between time points in Sample E, in which transcriptional changes were controlled for.
