# Supplementary material for: MetaRibo-Seq measures translation in microbiomes
Source: Nat Commun. 2020 Jun 29;11:3268. doi: 10.1038/s41467-020-17081-z (PMC7324362; doi:10.1038/s41467-020-17081-z)
Supplement: Supplementary file 3 — Description of Additional Supplementary Files [file 41467_2020_17081_MOESM3_ESM.docx]

**Description of Additional Supplementary Files**

File Name: Supplementary Data 1

Description: Statistics on percentages of reads classified and unclassified at the genera level across all samples, technologies, and replicates.

File Name: Supplementary Data 2

Description: We display all correlations between replicates and technologies for Samples A, B, C, and D. For each sample, we provide correlations between metatranscriptomics replicates, MetaRibo-Seq replicates, metagenomics versus metatrancriptomics, metagenomics versus MetaRibo-Seq, metatranscriptomics versus MetaRibo-Seq, and metagenomics versus translation efficiency (TE – ratio of MetaRibo-Seq and metatranscriptomics).

File Name: Supplementary Data 3

Description: Mapping statistics to *de novo* references. This table shows the number of reads, percentage of reads mapping to the assembly, and percentage of reads overlapping regions annotated as coding.

File Name: Supplementary Data 4

Description: We provide sequences for every protein in Samples A, B, C, and D that is translated at a different level than transcribed in the fecal microbiota. These proteins (.faa) and 70 percent identity clustering (.cltsr) are provided for Samples A, B, C, and D. The representative sequences for clusters (.faa) with more than 5 sequences for samples are also provided. The sequence name itself denotes which sample the sequence is found in. Any sequence that begins with a specific identifier can be linked to a sample: HDALDHFB = Sample A, HENMDNCI =Sample B, PJJNKMKO = Sample C, GPBGFMPE = Sample D. Blast2GO results for the representative sequences of consistent clusters are provided.

File Name: Supplementary Data 5

Description: DESeq2 results of Sample E over time. Pairwise comparison of metatranscriptomics in *E. coli* between Sample E and Sample E2 (transcriptional changes), pairwise comparison of MetaRibo-Seq in *E. coli* between Sample E and Sample E2 (translational changes), and pairwise comparison between MetaRibo-Seq, controlling for metatranscriptomics, in *E. coli* between Sample E and Sample E2 (translational regulation). Negative log2fold change indicate that the levels measured in Sample E2 were lower than in Sample E. These same analyses were performed again, except small proteins were included in the analyses. For all comparisons, p values and Benjamini and Hockberg corrected p values are provided.

File Name: Supplementary Data 6

Description: First, we display MetaRibo-Seq RPKM values for all homologs predicted in Samples A-E corresponding the 623 small protein families, a subset of the initial 4,539 small gene families. Second, we display MetaRibo-Seq RPKM values for all homologs predicted in Samples A-E corresponding to the 2,091 small protein families additionally identified. Third, we show family information for the 623 small protein families as previously predicted^4^. Fourth, we provide additional information pertaining to these 2,091 clusters, including details when applicable such as RNAcode p-values, protein domains, transmembrane predictions, secretion predictions, genomic neighborhood analyses, homology to 4K analyses, and taxonomy (see Methods). Fifth, we provide the blast results to identify clusters within the 2,091 that share homology to the 4K.

File Name: Supplementary Data 7

Description: This file contains the DNA sequences, amino acid sequences, and taxonomy of the 2,091 small protein families, which can each be interactively visualized as Krona plots.
