# Supplementary material for: MetaRibo-Seq measures translation in microbiomes
Source: Nat Commun. 2020 Jun 29;11:3268. doi: 10.1038/s41467-020-17081-z (PMC7324362; doi:10.1038/s41467-020-17081-z)
Supplement: Supplementary file 10 — Supplementary Data 7 [file 41467_2020_17081_MOESM10_ESM.zip › File2/Confidence_VeryHigh_Taxonomy/132858_out.krona.html]

Javascript must be enabled to view this page.

members
magnitude
magnitudeUnassigned
count
unassigned
taxon
rank

132858\_out

12

superkingdom
2
12

1239
12
phylum

class
186801
12

order
186802
12

1897042
4
species

SRS016095\_contig\_number\_contig-100\_6273.111428SRS047014\_contig\_number\_contig-100\_14103.47847SRS047433\_contig\_number\_contig-100\_10084.10085SRS077392\_contig\_number\_contig-100\_12534.45168

family
186803
8

8
1898203

SRS012273\_contig\_number\_contig-100\_2214.123320SRS014613\_contig\_number\_contig-100\_738.42657SRS014923\_contig\_number\_35687SRS014979\_contig\_number\_35136SRS019068\_contig\_number\_71843SRS024663\_contig\_number\_contig-100\_6544.69892SRS143417\_contig\_number\_contig-100\_15668.102711SRS146812\_contig\_number\_29829
species
