# Supplementary material for: MetaRibo-Seq measures translation in microbiomes
Source: Nat Commun. 2020 Jun 29;11:3268. doi: 10.1038/s41467-020-17081-z (PMC7324362; doi:10.1038/s41467-020-17081-z)
Supplement: Supplementary file 10 — Supplementary Data 7 [file 41467_2020_17081_MOESM10_ESM.zip › File2/Confidence_VeryHigh_Taxonomy/278510_out.krona.html]

Javascript must be enabled to view this page.

members
magnitude
magnitudeUnassigned
count
unassigned
taxon
rank

278510\_out

6

superkingdom
5
2

1239
5
phylum

class
186801
5

order
186802
5

1
1897050

SRS019496\_contig\_number\_5579
species

1
216572

SRS971276\_contig\_number\_27153
family

family
1
541000

216851
1
genus

species

SRS015431\_contig\_number\_73543
853
1

2
186803
family

33042
1
genus

1
410072

SRS075773\_contig\_number\_29478
species

841
1
genus

species
1

SRS147377\_contig\_number\_contig-100\_949.63844
301302


SRS1055034\_contig\_number\_contig-100\_13293.53465
1
