# Supplementary material for: MetaRibo-Seq measures translation in microbiomes
Source: Nat Commun. 2020 Jun 29;11:3268. doi: 10.1038/s41467-020-17081-z (PMC7324362; doi:10.1038/s41467-020-17081-z)
Supplement: Supplementary file 10 — Supplementary Data 7 [file 41467_2020_17081_MOESM10_ESM.zip › File2/Confidence_VeryHigh_Taxonomy/51510_out.krona.html]

Javascript must be enabled to view this page.

members
magnitude
magnitudeUnassigned
count
unassigned
taxon
rank

51510\_out

6

superkingdom
2
6

phylum
1239
6

class
186801
5

5
order
186802

5
family
541000

5
genus
1263

4

SRS017521\_contig\_number\_51025SRS049402\_contig\_number\_contig-100\_14366.55522SRS050422\_contig\_number\_contig-100\_1123.198106SRS057478\_contig\_number\_2887
species
2293207

1

SRS023914\_contig\_number\_contig-100\_9348.54998
2293176
species

1

SRS142712\_contig\_number\_1850
2292894
species
