# Supplementary material for: MetaRibo-Seq measures translation in microbiomes
Source: Nat Commun. 2020 Jun 29;11:3268. doi: 10.1038/s41467-020-17081-z (PMC7324362; doi:10.1038/s41467-020-17081-z)
Supplement: Supplementary file 10 — Supplementary Data 7 [file 41467_2020_17081_MOESM10_ESM.zip › File2/Confidence_VeryHigh_Taxonomy/135942_out.krona.html]

Javascript must be enabled to view this page.

members
magnitude
magnitudeUnassigned
count
unassigned
taxon
rank

135942\_out

6

2
superkingdom
6

1239
phylum
6

186801
class
6

6
order
186802

186806
family
3

1730
3
genus

species

SRS015431\_contig\_number\_22409SRS050925\_contig\_number\_contig-100\_1265.198962SRS148159\_contig\_number\_30415
3
142586

family
2
541000

genus
1
1263

46228

SRS020233\_contig\_number\_contig-100\_1393.343740
species
1

2291991
1
species

SRS012273\_contig\_number\_26734

family
1
186803

572511
genus
1

1
species

SRS015217\_contig\_number\_4368
33039
