# Supplementary material for: MetaRibo-Seq measures translation in microbiomes
Source: Nat Commun. 2020 Jun 29;11:3268. doi: 10.1038/s41467-020-17081-z (PMC7324362; doi:10.1038/s41467-020-17081-z)
Supplement: Supplementary file 10 — Supplementary Data 7 [file 41467_2020_17081_MOESM10_ESM.zip › File2/Confidence_VeryHigh_Taxonomy/202721_out.krona.html]

Javascript must be enabled to view this page.

members
magnitude
magnitudeUnassigned
count
unassigned
taxon
rank

202721\_out

5

superkingdom
5
2

201174
phylum
5

1760
class
5

order
4
2037

2049
family
4

1654
4
genus


SRS104519\_contig\_number\_30359SRS104521\_contig\_number\_19101SRS144237\_contig\_number\_17314
1655
3
species

712118

SRS142896\_contig\_number\_261
1
species

order
1
85006

1268
1
family

32207
genus
1

172042

SRS014690\_contig\_number\_contig-100\_8679.67595
1
species
