# Supplementary material for: MetaRibo-Seq measures translation in microbiomes
Source: Nat Commun. 2020 Jun 29;11:3268. doi: 10.1038/s41467-020-17081-z (PMC7324362; doi:10.1038/s41467-020-17081-z)
Supplement: Supplementary file 10 — Supplementary Data 7 [file 41467_2020_17081_MOESM10_ESM.zip › File2/Confidence_VeryHigh_Taxonomy/79328_out.krona.html]

Javascript must be enabled to view this page.

members
magnitude
magnitudeUnassigned
count
unassigned
taxon
rank

79328\_out

4

4
superkingdom
2

2
phylum
201174

2
class
1760

85006
order
2

family
1268
2

2
32207
genus

1

SRS893173\_contig\_number\_16888
43675
species


SRS148414\_contig\_number\_contig-100\_944.38305
1
species
1739462

1
phylum
976

200643
class
1

1
171549
order

1
family
1853231

574697
genus
1

SRS054352\_contig\_number\_contig-100\_35324.73620

1224
phylum
1

1
class
1236

order
135625
1

family
712
1

1
416916
genus

1

SRS893318\_contig\_number\_contig-100\_4598.131023
732
species
