# Supplementary material for: MetaRibo-Seq measures translation in microbiomes
Source: Nat Commun. 2020 Jun 29;11:3268. doi: 10.1038/s41467-020-17081-z (PMC7324362; doi:10.1038/s41467-020-17081-z)
Supplement: Supplementary file 10 — Supplementary Data 7 [file 41467_2020_17081_MOESM10_ESM.zip › File2/Confidence_VeryHigh_Taxonomy/147129_out.krona.html]

Javascript must be enabled to view this page.

members
magnitude
magnitudeUnassigned
count
unassigned
taxon
rank

147129\_out

7

2
superkingdom
7

7
1239
phylum

7
186801
class

2
7

SRS144506\_contig\_number\_57460SRS144537\_contig\_number\_39276
order
186802

541000
family
1

genus
216851
1

1946510
species
1

SRS148196\_contig\_number\_27162

2
family
31979

genus
580596
2

species
2292298

SRS104036\_contig\_number\_4732SRS147271\_contig\_number\_42409
2

2
186803
family

2

SRS018888\_contig\_number\_3435SRS1041138\_contig\_number\_9915
1952134
species
