# Supplementary material for: MetaRibo-Seq measures translation in microbiomes
Source: Nat Commun. 2020 Jun 29;11:3268. doi: 10.1038/s41467-020-17081-z (PMC7324362; doi:10.1038/s41467-020-17081-z)
Supplement: Supplementary file 10 — Supplementary Data 7 [file 41467_2020_17081_MOESM10_ESM.zip › File2/Confidence_VeryHigh_Taxonomy/372662_out.krona.html]

Javascript must be enabled to view this page.

members
magnitude
magnitudeUnassigned
count
unassigned
taxon
rank

372662\_out

39

39
superkingdom
2

phylum
1239
39

2292898
species
8

SRS048060\_contig\_number\_13401SRS097958\_contig\_number\_20103SRS098881\_contig\_number\_28428SRS1054691\_contig\_number\_11144SRS143417\_contig\_number\_contig-100\_2788.185905SRS147139\_contig\_number\_41095SRS148721\_contig\_number\_53001SRS148874\_contig\_number\_16917

186801
class
31

186802
order
31

11
31979
family

genus
1485
9

59620
species
6

SRS054905\_contig\_number\_8443SRS054905\_contig\_number\_26503SRS076976\_contig\_number\_14661SRS077024\_contig\_number\_contig-100\_726.98646SRS104975\_contig\_number\_11706SRS147022\_contig\_number\_contig-100\_314.89035

2

SRS047433\_contig\_number\_contig-100\_58.127340SRS056273\_contig\_number\_contig-100\_60.189000
1262786
species


SRS056273\_contig\_number\_2373
1
species
2293045

species
2292274

SRS098514\_contig\_number\_contig-100\_499.4374
1

1

SRS062654\_contig\_number\_21036
species
2291989

family
186803
20

9
genus
841


SRS097920\_contig\_number\_contig-100\_934.193714
1
species
2049040


SRS024132\_contig\_number\_20736SRS043841\_contig\_number\_contig-100\_92.82817SRS044535\_contig\_number\_13647SRS1055083\_contig\_number\_2500SRS144297\_contig\_number\_13923SRS148159\_contig\_number\_22481SRS149879\_contig\_number\_5467
7
2293144
species


SRS893341\_contig\_number\_contig-100\_1915.95041
1
1262941
species

species
1898203

SRS049446\_contig\_number\_contig-100\_9008.69701SRS053398\_contig\_number\_21440SRS075341\_contig\_number\_23939SRS077194\_contig\_number\_29370SRS098073\_contig\_number\_1189SRS104084\_contig\_number\_11674SRS104197\_contig\_number\_contig-100\_29.29SRS147766\_contig\_number\_16094SRS149325\_contig\_number\_contig-100\_1156.49933SRS893292\_contig\_number\_5289SRS893300\_contig\_number\_8974
11
