# Supplementary material for: MetaRibo-Seq measures translation in microbiomes
Source: Nat Commun. 2020 Jun 29;11:3268. doi: 10.1038/s41467-020-17081-z (PMC7324362; doi:10.1038/s41467-020-17081-z)
Supplement: Supplementary file 10 — Supplementary Data 7 [file 41467_2020_17081_MOESM10_ESM.zip › File2/Confidence_VeryHigh_Taxonomy/93987_out.krona.html]

Javascript must be enabled to view this page.

members
magnitude
magnitudeUnassigned
count
unassigned
taxon
rank

93987\_out

11


SRS022143\_contig\_number\_34834SRS100027\_contig\_number\_contig-100\_14829.14829SRS101439\_contig\_number\_29882
3

superkingdom
2759
1

4751
kingdom
1

451864
subkingdom
1

1
4890
phylum

1
subphylum
147538

147549
class
1

5185
order
1

1
family
40289

genus
36048
1

1
39416
species

SRS019607\_contig\_number\_21300

7
2
superkingdom

phylum

SRS144368\_contig\_number\_10101SRS144376\_contig\_number\_9556
1239
2
7

5
class
909932

1843489
order
3

family
31977
3

906
genus
3

3
187326
species

SRS012916\_contig\_number\_28213SRS048791\_contig\_number\_25575SRS075404\_contig\_number\_22139

order
1843488
2

909930
family
2

33024
genus
2

2
626940
species

SRS048164\_contig\_number\_30104SRS053356\_contig\_number\_12929
