# Supplementary material for: MetaRibo-Seq measures translation in microbiomes
Source: Nat Commun. 2020 Jun 29;11:3268. doi: 10.1038/s41467-020-17081-z (PMC7324362; doi:10.1038/s41467-020-17081-z)
Supplement: Supplementary file 10 — Supplementary Data 7 [file 41467_2020_17081_MOESM10_ESM.zip › File2/Confidence_VeryHigh_Taxonomy/500_out.krona.html]

Javascript must be enabled to view this page.

members
magnitude
magnitudeUnassigned
count
unassigned
taxon
rank

500\_out

10

10
superkingdom
2

10
1239
phylum

91061
class
10

order
186826
10

10
1300
family

10
1301
genus

1

SRS057886\_contig\_number\_contig-100\_7233.156225
1302
species

1303
species
1

1

SRS051941\_contig\_number\_7652
1077464
subspecies

1305
species
7

SRS011126\_contig\_number\_25419SRS012922\_contig\_number\_contig-100\_11902.80463.80463SRS015989\_contig\_number\_contig-100\_3621.89789SRS044448\_contig\_number\_11767SRS049289\_contig\_number\_contig-100\_7902.7903SRS142608\_contig\_number\_contig-100\_6573.110041SRS144621\_contig\_number\_344


SRS098624\_contig\_number\_contig-100\_3436.142916
1
species
469609
