# Supplementary material for: MetaRibo-Seq measures translation in microbiomes
Source: Nat Commun. 2020 Jun 29;11:3268. doi: 10.1038/s41467-020-17081-z (PMC7324362; doi:10.1038/s41467-020-17081-z)
Supplement: Supplementary file 10 — Supplementary Data 7 [file 41467_2020_17081_MOESM10_ESM.zip › File2/Confidence_VeryHigh_Taxonomy/60641_out.krona.html]

Javascript must be enabled to view this page.

members
magnitude
magnitudeUnassigned
count
unassigned
taxon
rank

60641\_out

14

superkingdom
2
14

phylum
14
1239

class
186801
10

10
186802
order

family
10
186806

5

SRS017521\_contig\_number\_contig-100\_506.249444SRS098717\_contig\_number\_contig-100\_978.90780SRS1054691\_contig\_number\_11000SRS142599\_contig\_number\_1307SRS148721\_contig\_number\_35010
genus
1730
10

species

SRS014736\_contig\_number\_7421SRS015217\_contig\_number\_18393
2058290
2

457402
1
species

SRS144506\_contig\_number\_11420


SRS019601\_contig\_number\_30885SRS146888\_contig\_number\_10556
species
2
2302950

class
4
526524

order
4
526525

128827
4
family

1505663
1
genus

species

SRS146888\_contig\_number\_1916
1522
1

552396
1

SRS015217\_contig\_number\_26028
species

genus
2
2057233


SRS057717\_contig\_number\_4490SRS146888\_contig\_number\_13927
species
2
31971
