# Supplementary material for: MetaRibo-Seq measures translation in microbiomes
Source: Nat Commun. 2020 Jun 29;11:3268. doi: 10.1038/s41467-020-17081-z (PMC7324362; doi:10.1038/s41467-020-17081-z)
Supplement: Supplementary file 10 — Supplementary Data 7 [file 41467_2020_17081_MOESM10_ESM.zip › File2/Confidence_VeryHigh_Taxonomy/201325_out.krona.html]

Javascript must be enabled to view this page.

members
magnitude
magnitudeUnassigned
count
unassigned
taxon
rank

201325\_out

5

SRS013521\_contig\_number\_7609
1

2
superkingdom
4

1239
phylum
4

186801
class
4

186802
4
order

family
4
31979

1485
genus
4

species
4
1262813

SRS015782\_contig\_number\_10600SRS016954\_contig\_number\_4031SRS047044\_contig\_number\_16295SRS078176\_contig\_number\_22929
