# Supplementary material for: MetaRibo-Seq measures translation in microbiomes
Source: Nat Commun. 2020 Jun 29;11:3268. doi: 10.1038/s41467-020-17081-z (PMC7324362; doi:10.1038/s41467-020-17081-z)
Supplement: Supplementary file 10 — Supplementary Data 7 [file 41467_2020_17081_MOESM10_ESM.zip › File2/Confidence_VeryHigh_Taxonomy/321384_out.krona.html]

Javascript must be enabled to view this page.

members
magnitude
magnitudeUnassigned
count
unassigned
taxon
rank

321384\_out

8

2
superkingdom
8

8
phylum
1239

class
186801
8

order
186802
8

family
31979
3

1

SRS142781\_contig\_number\_10433
genus
1485
3

species

SRS048164\_contig\_number\_2125SRS075984\_contig\_number\_18463
59620
2

1
541000
family

genus
1263
1


SRS142542\_contig\_number\_16604
species
46228
1

4
family
186803

1
658082

SRS075716\_contig\_number\_4826
species

3
841
genus

species

SRS014287\_contig\_number\_20449SRS043411\_contig\_number\_contig-100\_1412.110324
166486
2

1
species

SRS063040\_contig\_number\_35832
2049040
