# Supplementary material for: MetaRibo-Seq measures translation in microbiomes
Source: Nat Commun. 2020 Jun 29;11:3268. doi: 10.1038/s41467-020-17081-z (PMC7324362; doi:10.1038/s41467-020-17081-z)
Supplement: Supplementary file 10 — Supplementary Data 7 [file 41467_2020_17081_MOESM10_ESM.zip › File2/Confidence_VeryHigh_Taxonomy/2826_out.krona.html]

Javascript must be enabled to view this page.

members
magnitude
magnitudeUnassigned
count
unassigned
taxon
rank

2826\_out

6


SRS103987\_contig\_number\_contig-100\_34664.76364SRS104084\_contig\_number\_contig-100\_19578.19579SRS1041132\_contig\_number\_contig-100\_24732.24733
3

superkingdom
2759
1

4751
1
kingdom

451864
1
subkingdom

phylum
1
4890

subphylum
147538
1

class
1
147550

subclass
222544
1

order
1
5139

family
35718
1

35719
1
genus

1
35720
species

SRS019068\_contig\_number\_contig-100\_19053.197261

superkingdom
2
2

2
1239
phylum

class
2
186801

186802
2
order

family
216572
2

genus
459786
2

species

SRS013687\_contig\_number\_contig-100\_6217.146191
1
1262910

1235797
1

SRS013098\_contig\_number\_48614
species
