# Supplementary material for: MetaRibo-Seq measures translation in microbiomes
Source: Nat Commun. 2020 Jun 29;11:3268. doi: 10.1038/s41467-020-17081-z (PMC7324362; doi:10.1038/s41467-020-17081-z)
Supplement: Supplementary file 10 — Supplementary Data 7 [file 41467_2020_17081_MOESM10_ESM.zip › File2/Confidence_VeryHigh_Taxonomy/5748_out.krona.html]

Javascript must be enabled to view this page.

members
magnitude
magnitudeUnassigned
count
unassigned
taxon
rank

5748\_out

6

2
superkingdom
6

1239
6
phylum

class
6
186801

186802
2

SRS015578\_contig\_number\_14056SRS103987\_contig\_number\_8459
6
order

4
family
186803

4
genus
572511


SRS016267\_contig\_number\_12829SRS057717\_contig\_number\_7464SRS148253\_contig\_number\_8677SRS893259\_contig\_number\_4969
33038
species
4
