# Supplementary material for: MetaRibo-Seq measures translation in microbiomes
Source: Nat Commun. 2020 Jun 29;11:3268. doi: 10.1038/s41467-020-17081-z (PMC7324362; doi:10.1038/s41467-020-17081-z)
Supplement: Supplementary file 10 — Supplementary Data 7 [file 41467_2020_17081_MOESM10_ESM.zip › File2/Confidence_VeryHigh_Taxonomy/210544_out.krona.html]

Javascript must be enabled to view this page.

members
magnitude
magnitudeUnassigned
count
unassigned
taxon
rank

210544\_out

9

2
superkingdom
9

9
phylum
1239

186801
9
class

186802
1

SRS893259\_contig\_number\_contig-100\_15601.15602
9
order

186803
family
5

1898203

SRS014459\_contig\_number\_37598SRS076976\_contig\_number\_10552SRS149181\_contig\_number\_contig-100\_56.180146
3
species

1
genus
572511

765821

SRS055017\_contig\_number\_34333
1
species

1
species
2292903

SRS1041091\_contig\_number\_3795

541000
1
family

genus
1
1263

2292252

SRS143070\_contig\_number\_8578
species
1

family
2
31979

1485
genus
2

species
1

SRS046712\_contig\_number\_1460
1720194

1262824

SRS024549\_contig\_number\_contig-100\_1064.31259
1
species
