# Supplementary material for: MetaRibo-Seq measures translation in microbiomes
Source: Nat Commun. 2020 Jun 29;11:3268. doi: 10.1038/s41467-020-17081-z (PMC7324362; doi:10.1038/s41467-020-17081-z)
Supplement: Supplementary file 10 — Supplementary Data 7 [file 41467_2020_17081_MOESM10_ESM.zip › File2/Confidence_VeryHigh_Taxonomy/221439_out.krona.html]

Javascript must be enabled to view this page.

members
magnitude
magnitudeUnassigned
count
unassigned
taxon
rank

221439\_out

65

2
superkingdom
65

63
1239
phylum

186801
class
63

186802

SRS013965\_contig\_number\_17077SRS018427\_contig\_number\_contig-100\_46143.89081SRS020328\_contig\_number\_contig-100\_8192.142604SRS049995\_contig\_number\_10485SRS050941\_contig\_number\_contig-100\_3216.3217SRS077127\_contig\_number\_contig-100\_6596.6597SRS097920\_contig\_number\_contig-100\_23421.110005SRS143372\_contig\_number\_contig-100\_21337.21338
8
order
63

family
31979
2

2
genus
1485

1

SRS144183\_contig\_number\_4760
2292996
species


SRS051031\_contig\_number\_13467
1262785
species
1

7
186803
family

28050
genus
5

5

SRS014855\_contig\_number\_11675SRS017191\_contig\_number\_contig-100\_6099.99572SRS076804\_contig\_number\_contig-100\_130.131SRS078176\_contig\_number\_17066SRS144135\_contig\_number\_contig-100\_1345.148655
species
28052

1
572511
genus

1

SRS148784\_contig\_number\_contig-100\_62.38430
species
2292986

841
genus
1

166486

SRS077502\_contig\_number\_8030
species
1

46
family
186806

genus
6

SRS011405\_contig\_number\_13206SRS015190\_contig\_number\_12042SRS044535\_contig\_number\_contig-100\_3714.100323SRS063370\_contig\_number\_2677SRS097889\_contig\_number\_contig-100\_3534.202472SRS144297\_contig\_number\_729
46
1730

species

SRS015578\_contig\_number\_33045SRS019267\_contig\_number\_contig-100\_181.52995SRS047044\_contig\_number\_17793SRS050752\_contig\_number\_contig-100\_31476.71641SRS146812\_contig\_number\_50108SRS148159\_contig\_number\_3192
1262892
6

13

SRS013476\_contig\_number\_12762SRS013951\_contig\_number\_26869SRS014979\_contig\_number\_contig-100\_633.94764SRS018984\_contig\_number\_22609SRS019161\_contig\_number\_contig-100\_11.222868SRS052027\_contig\_number\_18727SRS056519\_contig\_number\_contig-100\_622.110844SRS065504\_contig\_number\_19795SRS100021\_contig\_number\_7178SRS143342\_contig\_number\_21194SRS143780\_contig\_number\_27221SRS147919\_contig\_number\_5415SRS148091\_contig\_number\_11083
142586
species

20

SRS014235\_contig\_number\_45309SRS015431\_contig\_number\_7316SRS015663\_contig\_number\_27463SRS019685\_contig\_number\_6431SRS019787\_contig\_number\_5232SRS023346\_contig\_number\_contig-100\_2796.50241SRS023715\_contig\_number\_21600SRS043667\_contig\_number\_contig-100\_4327.29194SRS058723\_contig\_number\_5910SRS075878\_contig\_number\_contig-100\_4344.105782SRS077335\_contig\_number\_1562SRS098827\_contig\_number\_12611SRS104311\_contig\_number\_32788SRS142890\_contig\_number\_contig-100\_25437.79369SRS144537\_contig\_number\_37364SRS145308\_contig\_number\_contig-100\_3588.3589SRS146813\_contig\_number\_18535SRS147271\_contig\_number\_32411SRS147377\_contig\_number\_contig-100\_2560.44013SRS148253\_contig\_number\_8381
1897026
species

species

SRS143070\_contig\_number\_4274
1262889
1

976
phylum
2

200643
class
2

order
171549
2

2
family
815

2
genus
816

1

SRS014736\_contig\_number\_contig-100\_15033.15034
384639
species


SRS063040\_contig\_number\_35945
246787
species
1
