# Supplementary material for: MetaRibo-Seq measures translation in microbiomes
Source: Nat Commun. 2020 Jun 29;11:3268. doi: 10.1038/s41467-020-17081-z (PMC7324362; doi:10.1038/s41467-020-17081-z)
Supplement: Supplementary file 10 — Supplementary Data 7 [file 41467_2020_17081_MOESM10_ESM.zip › File2/Confidence_VeryHigh_Taxonomy/357852_out.krona.html]

Javascript must be enabled to view this page.

members
magnitude
magnitudeUnassigned
count
unassigned
taxon
rank

357852\_out

4

4
2
superkingdom

1
1239
phylum

class
186801
1

order
186802
1

family
31979
1

1485
1
genus

species

SRS052697\_contig\_number\_34533
1
59620

976
3
phylum

class
3
200643

order
3
171549

family
171552
2

genus
2
838


SRS013521\_contig\_number\_286SRS049995\_contig\_number\_2479
species
2292054
2

1
815
family

816
1
genus


SRS142503\_contig\_number\_17923
species
1
329854
