# Supplementary material for: MetaRibo-Seq measures translation in microbiomes
Source: Nat Commun. 2020 Jun 29;11:3268. doi: 10.1038/s41467-020-17081-z (PMC7324362; doi:10.1038/s41467-020-17081-z)
Supplement: Supplementary file 10 — Supplementary Data 7 [file 41467_2020_17081_MOESM10_ESM.zip › File2/Confidence_VeryHigh_Taxonomy/36238_out.krona.html]

Javascript must be enabled to view this page.

members
magnitude
magnitudeUnassigned
count
unassigned
taxon
rank

36238\_out

6

6
superkingdom
2

6
phylum
1239

class
6
186801


SRS016954\_contig\_number\_6088SRS023914\_contig\_number\_987SRS148091\_contig\_number\_12842
order
3
186802
6

172733

SRS143991\_contig\_number\_contig-100\_11271.62595
species
1

family
2
186803

2
genus
572511

species
1
1262756

SRS149879\_contig\_number\_17823


SRS018817\_contig\_number\_4426
418240
1
species
