# Supplementary material for: MetaRibo-Seq measures translation in microbiomes
Source: Nat Commun. 2020 Jun 29;11:3268. doi: 10.1038/s41467-020-17081-z (PMC7324362; doi:10.1038/s41467-020-17081-z)
Supplement: Supplementary file 10 — Supplementary Data 7 [file 41467_2020_17081_MOESM10_ESM.zip › File2/Confidence_VeryHigh_Taxonomy/178573_out.krona.html]

Javascript must be enabled to view this page.

members
magnitude
magnitudeUnassigned
count
unassigned
taxon
rank

178573\_out

57

57
superkingdom
2

57
phylum
976

class
57
200643

order
57
171549

family
57
171550

genus
57
239759

1499682
species
1

SRS077552\_contig\_number\_15516

1
species
28117

SRS045645\_contig\_number\_6711


SRS011302\_contig\_number\_26910SRS012273\_contig\_number\_21472SRS013638\_contig\_number\_contig-100\_8877.44759SRS014683\_contig\_number\_11292SRS015890\_contig\_number\_21408SRS016056\_contig\_number\_7325SRS016335\_contig\_number\_contig-100\_1181.238702SRS016381\_contig\_number\_642SRS016897\_contig\_number\_contig-100\_144.21713SRS018541\_contig\_number\_12767SRS018575\_contig\_number\_5626SRS018656\_contig\_number\_798SRS018836\_contig\_number\_26294SRS018984\_contig\_number\_1850SRS019030\_contig\_number\_6331SRS019178\_contig\_number\_1203SRS022071\_contig\_number\_10349SRS022524\_contig\_number\_7582SRS024388\_contig\_number\_contig-100\_2409.86539SRS046717\_contig\_number\_contig-100\_91.85936SRS049402\_contig\_number\_1699SRS049446\_contig\_number\_12690SRS053649\_contig\_number\_12015SRS078242\_contig\_number\_10704SRS098655\_contig\_number\_9450SRS098717\_contig\_number\_12730SRS101376\_contig\_number\_1273SRS1041132\_contig\_number\_1487SRS1041157\_contig\_number\_5168SRS104400\_contig\_number\_20717SRS104975\_contig\_number\_980SRS1055056\_contig\_number\_3100SRS140492\_contig\_number\_26776SRS140513\_contig\_number\_15660SRS142890\_contig\_number\_36716SRS143895\_contig\_number\_8306SRS143991\_contig\_number\_21616SRS147346\_contig\_number\_contig-100\_2728.327201SRS148196\_contig\_number\_36155SRS892882\_contig\_number\_11721SRS893327\_contig\_number\_5805SRS893341\_contig\_number\_15059SRS893382\_contig\_number\_3809
species
43
214856


SRS014613\_contig\_number\_14529SRS018427\_contig\_number\_32249SRS064276\_contig\_number\_36089SRS077086\_contig\_number\_1523SRS104636\_contig\_number\_contig-100\_2133.119323SRS147139\_contig\_number\_27317SRS149325\_contig\_number\_7719
328813
7
species


SRS017916\_contig\_number\_3167SRS019381\_contig\_number\_19410SRS055017\_contig\_number\_contig-100\_10122.112345SRS105153\_contig\_number\_17937SRS893342\_contig\_number\_2824
5
species
1872444
