# Supplementary material for: MetaRibo-Seq measures translation in microbiomes
Source: Nat Commun. 2020 Jun 29;11:3268. doi: 10.1038/s41467-020-17081-z (PMC7324362; doi:10.1038/s41467-020-17081-z)
Supplement: Supplementary file 10 — Supplementary Data 7 [file 41467_2020_17081_MOESM10_ESM.zip › File2/Confidence_VeryHigh_Taxonomy/333239_out.krona.html]

Javascript must be enabled to view this page.

members
magnitude
magnitudeUnassigned
count
unassigned
taxon
rank

333239\_out

129

2
superkingdom
129

976
129
phylum

200643
class
129

171549
order
129

family
129
815

816
genus
129

1262742

SRS105082\_contig\_number\_1363
1
species

species
1

SRS077552\_contig\_number\_25268
2302926

species
127
46506

SRS011302\_contig\_number\_11701SRS012273\_contig\_number\_9096SRS013382\_contig\_number\_875SRS013638\_contig\_number\_1311SRS013687\_contig\_number\_1722SRS013800\_contig\_number\_467SRS014412\_contig\_number\_14140SRS014683\_contig\_number\_7372SRS014923\_contig\_number\_16453SRS015431\_contig\_number\_19055SRS015579\_contig\_number\_6612SRS015663\_contig\_number\_30123SRS015782\_contig\_number\_15470SRS015794\_contig\_number\_1142SRS016056\_contig\_number\_565SRS016203\_contig\_number\_2126SRS016954\_contig\_number\_22607SRS017247\_contig\_number\_3036SRS017701\_contig\_number\_15309SRS017745\_contig\_number\_2660SRS017821\_contig\_number\_11996SRS017859\_contig\_number\_819SRS018133\_contig\_number\_4885SRS018817\_contig\_number\_1001SRS018888\_contig\_number\_9146SRS018936\_contig\_number\_950SRS019030\_contig\_number\_1691SRS019068\_contig\_number\_67558SRS019161\_contig\_number\_15337SRS019178\_contig\_number\_1047SRS019397\_contig\_number\_5090SRS019968\_contig\_number\_2351SRS020271\_contig\_number\_1539SRS020394\_contig\_number\_2314SRS020869\_contig\_number\_21336SRS021484\_contig\_number\_14573SRS022071\_contig\_number\_16215SRS022137\_contig\_number\_4244SRS022713\_contig\_number\_9SRS023583\_contig\_number\_2590SRS023914\_contig\_number\_13839SRS024075\_contig\_number\_1529SRS024388\_contig\_number\_5331SRS024435\_contig\_number\_27591SRS024625\_contig\_number\_8253SRS043001\_contig\_number\_7036SRS043768\_contig\_number\_14230SRS045826\_contig\_number\_5780SRS046369\_contig\_number\_11817SRS046717\_contig\_number\_1339SRS047044\_contig\_number\_5699SRS048164\_contig\_number\_15234SRS049712\_contig\_number\_23706SRS049773\_contig\_number\_20765SRS049896\_contig\_number\_11646SRS049900\_contig\_number\_14046SRS050925\_contig\_number\_6340SRS050941\_contig\_number\_1558SRS051031\_contig\_number\_14764SRS053649\_contig\_number\_17166SRS055017\_contig\_number\_28008SRS055982\_contig\_number\_11740SRS057478\_contig\_number\_304SRS057480\_contig\_number\_10331SRS058770\_contig\_number\_13994SRS063489\_contig\_number\_1364SRS064645\_contig\_number\_282SRS064757\_contig\_number\_7965SRS065397\_contig\_number\_928SRS075821\_contig\_number\_14878SRS076876\_contig\_number\_802SRS076976\_contig\_number\_4274SRS077454\_contig\_number\_2952SRS077552\_contig\_number\_11807SRS077589\_contig\_number\_514SRS078242\_contig\_number\_12876SRS097920\_contig\_number\_23151SRS098061\_contig\_number\_8231SRS098571\_contig\_number\_31111SRS098881\_contig\_number\_6511SRS100021\_contig\_number\_2846SRS101433\_contig\_number\_7139SRS1041032\_contig\_number\_9367SRS1041036\_contig\_number\_248SRS1041039\_contig\_number\_20014SRS1041095\_contig\_number\_1029SRS1041132\_contig\_number\_741SRS1041133\_contig\_number\_14170SRS1041137\_contig\_number\_14505SRS1041139\_contig\_number\_1084SRS1041143\_contig\_number\_3120SRS1041144\_contig\_number\_522SRS1041147\_contig\_number\_496SRS1041157\_contig\_number\_1410SRS104400\_contig\_number\_30392SRS104912\_contig\_number\_13430SRS1054716\_contig\_number\_16010SRS1055069\_contig\_number\_200SRS1055076\_contig\_number\_12942SRS143070\_contig\_number\_12522SRS143148\_contig\_number\_1184SRS143372\_contig\_number\_7954SRS143417\_contig\_number\_31286SRS143523\_contig\_number\_14725SRS143598\_contig\_number\_12396SRS143722\_contig\_number\_10494SRS143876\_contig\_number\_10387SRS143991\_contig\_number\_25242SRS144135\_contig\_number\_24973SRS144506\_contig\_number\_12908SRS145497\_contig\_number\_2507SRS146813\_contig\_number\_23727SRS147039\_contig\_number\_1996SRS147271\_contig\_number\_3772SRS148511\_contig\_number\_9015SRS148721\_contig\_number\_27340SRS148784\_contig\_number\_21540SRS149075\_contig\_number\_11872SRS149181\_contig\_number\_3031SRS893172\_contig\_number\_1738SRS893187\_contig\_number\_2846SRS893270\_contig\_number\_10412SRS893279\_contig\_number\_4729SRS893327\_contig\_number\_2166SRS893358\_contig\_number\_3864SRS893378\_contig\_number\_19480SRS971276\_contig\_number\_4902
