# Supplementary material for: MetaRibo-Seq measures translation in microbiomes
Source: Nat Commun. 2020 Jun 29;11:3268. doi: 10.1038/s41467-020-17081-z (PMC7324362; doi:10.1038/s41467-020-17081-z)
Supplement: Supplementary file 10 — Supplementary Data 7 [file 41467_2020_17081_MOESM10_ESM.zip › File2/Confidence_VeryHigh_Taxonomy/206652_out.krona.html]

Javascript must be enabled to view this page.

members
magnitude
magnitudeUnassigned
count
unassigned
taxon
rank

206652\_out

5

superkingdom
5
2

phylum
5
1239

class
5
186801

186802
order
5

541000
family
5

genus
4

SRS019161\_contig\_number\_contig-100\_12427.56210SRS023971\_contig\_number\_contig-100\_21875.21875SRS049712\_contig\_number\_contig-100\_6028.123470SRS143148\_contig\_number\_contig-100\_13875.123103
216851
5

853
species
1

SRS142980\_contig\_number\_1856
