# Supplementary material for: MetaRibo-Seq measures translation in microbiomes
Source: Nat Commun. 2020 Jun 29;11:3268. doi: 10.1038/s41467-020-17081-z (PMC7324362; doi:10.1038/s41467-020-17081-z)
Supplement: Supplementary file 10 — Supplementary Data 7 [file 41467_2020_17081_MOESM10_ESM.zip › File2/Confidence_VeryHigh_Taxonomy/93310_out.krona.html]

Javascript must be enabled to view this page.

members
magnitude
magnitudeUnassigned
count
unassigned
taxon
rank

93310\_out

4

2
superkingdom
4

4
phylum
1239

4
186801
class

order
186802
4

family
186803
3

39491
species

SRS043768\_contig\_number\_25991SRS075821\_contig\_number\_2362SRS893369\_contig\_number\_1635
3

31979
family
1

genus
1485
1

species

SRS1055043\_contig\_number\_23051
2292204
1
