# Supplementary material for: MetaRibo-Seq measures translation in microbiomes
Source: Nat Commun. 2020 Jun 29;11:3268. doi: 10.1038/s41467-020-17081-z (PMC7324362; doi:10.1038/s41467-020-17081-z)
Supplement: Supplementary file 10 — Supplementary Data 7 [file 41467_2020_17081_MOESM10_ESM.zip › File2/Confidence_VeryHigh_Taxonomy/392318_out.krona.html]

Javascript must be enabled to view this page.

members
magnitude
magnitudeUnassigned
count
unassigned
taxon
rank

392318\_out

15

superkingdom
2
12

12
1239
phylum

class
10
186801

order
186802
10

family
3
31979

1485
3
genus

59620
3
species

SRS050026\_contig\_number\_contig-100\_710.82241SRS146764\_contig\_number\_contig-100\_52.128810SRS149244\_contig\_number\_9743

family
541000
4

946234
2
genus

1193534
1
species

SRS078665\_contig\_number\_contig-100\_170.77003

species

SRS049446\_contig\_number\_contig-100\_69.93107
292800
1


SRS104400\_contig\_number\_47410SRS105153\_contig\_number\_27496
species
2
1952410

1852363
2
species

SRS042628\_contig\_number\_33716SRS064276\_contig\_number\_contig-100\_3810.173322

186803
1
family

1952152
1
species

SRS014979\_contig\_number\_19029

1263004
2
species

SRS077231\_contig\_number\_contig-100\_242.150277SRS104311\_contig\_number\_34241


SRS023914\_contig\_number\_4041SRS063518\_contig\_number\_contig-100\_17566.138184SRS143466\_contig\_number\_6201
3
