# Supplementary material for: MetaRibo-Seq measures translation in microbiomes
Source: Nat Commun. 2020 Jun 29;11:3268. doi: 10.1038/s41467-020-17081-z (PMC7324362; doi:10.1038/s41467-020-17081-z)
Supplement: Supplementary file 10 — Supplementary Data 7 [file 41467_2020_17081_MOESM10_ESM.zip › File2/Confidence_VeryHigh_Taxonomy/724_out.krona.html]

Javascript must be enabled to view this page.

members
magnitude
magnitudeUnassigned
count
unassigned
taxon
rank

724\_out

201

superkingdom
2
201

27
201

SRS014412\_contig\_number\_3020SRS014948\_contig\_number\_contig-100\_9900.58688SRS015065\_contig\_number\_contig-100\_1085.228325SRS015578\_contig\_number\_2643SRS015890\_contig\_number\_contig-100\_511.163681SRS016954\_contig\_number\_contig-100\_1053.69311SRS017622\_contig\_number\_contig-100\_4567.4568SRS043768\_contig\_number\_contig-100\_2792.184229SRS043841\_contig\_number\_5304SRS046717\_contig\_number\_contig-100\_5841.50531SRS047044\_contig\_number\_contig-100\_2634.99751SRS049712\_contig\_number\_1333SRS049896\_contig\_number\_contig-100\_4634.136884SRS050026\_contig\_number\_contig-100\_1334.67992SRS054905\_contig\_number\_14433SRS058770\_contig\_number\_23563SRS064645\_contig\_number\_contig-100\_15969.15969SRS075716\_contig\_number\_contig-100\_4807.39863SRS077294\_contig\_number\_contig-100\_10085.10085SRS143876\_contig\_number\_37507SRS147139\_contig\_number\_contig-100\_3067.240821SRS147425\_contig\_number\_7480SRS148196\_contig\_number\_contig-100\_11615.124040SRS148253\_contig\_number\_contig-100\_2419.2420SRS148817\_contig\_number\_contig-100\_3741.92348SRS893369\_contig\_number\_2710SRS893383\_contig\_number\_contig-100\_4800.208926
1239
phylum

1897036
species
1

SRS971276\_contig\_number\_21803

169
class
186801

186802
order

SRS016495\_contig\_number\_12994SRS018656\_contig\_number\_contig-100\_4504.42935SRS019445\_contig\_number\_contig-100\_736.201091SRS023583\_contig\_number\_17155SRS024009\_contig\_number\_contig-100\_117.88471SRS048870\_contig\_number\_contig-100\_603.139721SRS063518\_contig\_number\_contig-100\_18926.105200SRS077024\_contig\_number\_8516SRS097889\_contig\_number\_contig-100\_1834.175420SRS1041133\_contig\_number\_14162SRS1041136\_contig\_number\_21006SRS1055038\_contig\_number\_3069
169
12

family
216572
1

1
genus
459786


SRS144362\_contig\_number\_43908
1
species
1945593

74
family
31979

2
73

SRS063040\_contig\_number\_43746SRS075984\_contig\_number\_contig-100\_178.166792
genus
1485

2293011
species

SRS013940\_contig\_number\_20954SRS013951\_contig\_number\_41199SRS014235\_contig\_number\_13913SRS014855\_contig\_number\_12632SRS014923\_contig\_number\_45927SRS015854\_contig\_number\_contig-100\_97.135446SRS016335\_contig\_number\_contig-100\_196.248701SRS017433\_contig\_number\_contig-100\_158.109967SRS018623\_contig\_number\_17086SRS019068\_contig\_number\_115380SRS021948\_contig\_number\_33755SRS022609\_contig\_number\_contig-100\_634.265463SRS023526\_contig\_number\_29172SRS024075\_contig\_number\_9887SRS042284\_contig\_number\_6567SRS043411\_contig\_number\_9074SRS048060\_contig\_number\_12582SRS048164\_contig\_number\_27075SRS050925\_contig\_number\_25612SRS056273\_contig\_number\_33441SRS058070\_contig\_number\_19435SRS075078\_contig\_number\_20987SRS077127\_contig\_number\_14163SRS077194\_contig\_number\_29075SRS098827\_contig\_number\_11577SRS104636\_contig\_number\_25008SRS105153\_contig\_number\_39485SRS143780\_contig\_number\_29747
28

2293015
species
1

SRS098644\_contig\_number\_35706

59620
species

SRS015217\_contig\_number\_3684SRS017191\_contig\_number\_18553SRS017821\_contig\_number\_17282SRS019496\_contig\_number\_2392SRS020233\_contig\_number\_59979SRS043001\_contig\_number\_10403SRS045645\_contig\_number\_contig-100\_2434.185645SRS047014\_contig\_number\_34300SRS051031\_contig\_number\_36114SRS055017\_contig\_number\_16254SRS055982\_contig\_number\_14209SRS062701\_contig\_number\_3447SRS075341\_contig\_number\_22940SRS076976\_contig\_number\_17815SRS078176\_contig\_number\_18059SRS098073\_contig\_number\_1379SRS1041037\_contig\_number\_14057SRS1041142\_contig\_number\_6525SRS104197\_contig\_number\_contig-100\_374.374SRS104693\_contig\_number\_12086SRS1055034\_contig\_number\_12048SRS142781\_contig\_number\_10762SRS142923\_contig\_number\_20973SRS143342\_contig\_number\_17312SRS144506\_contig\_number\_57470SRS144714\_contig\_number\_contig-100\_179.163145SRS147271\_contig\_number\_41854SRS147977\_contig\_number\_7350SRS148424\_contig\_number\_35775SRS893230\_contig\_number\_11176
30

species
1262845

SRS078419\_contig\_number\_contig-100\_267.227314
1


SRS015431\_contig\_number\_87852SRS015782\_contig\_number\_44712SRS142980\_contig\_number\_2842SRS143722\_contig\_number\_contig-100\_9.89320SRS147614\_contig\_number\_2066SRS893378\_contig\_number\_16496
6
2293030
species


SRS012969\_contig\_number\_25309SRS077849\_contig\_number\_contig-100\_122.198372
2
1262816
species

2293014
species
1

SRS019808\_contig\_number\_13965


SRS076804\_contig\_number\_20613
1
2293049
species


SRS049995\_contig\_number\_19268
1
species
1262810

1
1649459
genus

154046
species

SRS893270\_contig\_number\_contig-100\_1080.123400
1

family
186803
72

2
genus
33042

410072
species
1

SRS148159\_contig\_number\_33504

33043
species
1

SRS019693\_contig\_number\_3883

10
genus
698776

29360
species
10

SRS013476\_contig\_number\_39430SRS015694\_contig\_number\_5067SRS015794\_contig\_number\_3572SRS019787\_contig\_number\_9548SRS050998\_contig\_number\_5239SRS051882\_contig\_number\_13855SRS053214\_contig\_number\_14550SRS143148\_contig\_number\_23496SRS146812\_contig\_number\_57241SRS148784\_contig\_number\_26943

1898203
species

SRS011134\_contig\_number\_51864SRS011405\_contig\_number\_contig-100\_233.112775SRS012849\_contig\_number\_32298SRS015663\_contig\_number\_37007SRS016018\_contig\_number\_15747SRS016095\_contig\_number\_30230SRS016989\_contig\_number\_15984SRS017307\_contig\_number\_32387SRS018836\_contig\_number\_contig-100\_121.166804SRS019161\_contig\_number\_43033SRS024132\_contig\_number\_32481SRS024331\_contig\_number\_38171SRS024435\_contig\_number\_43105SRS042628\_contig\_number\_36868SRS043701\_contig\_number\_12137SRS044535\_contig\_number\_8710SRS052697\_contig\_number\_45871SRS053335\_contig\_number\_12404SRS065504\_contig\_number\_34013SRS075821\_contig\_number\_21353SRS076756\_contig\_number\_contig-100\_380.128990SRS077231\_contig\_number\_22647SRS103987\_contig\_number\_37792SRS1041038\_contig\_number\_16241SRS104400\_contig\_number\_59321SRS104485\_contig\_number\_5231SRS104975\_contig\_number\_contig-100\_211.135323SRS1054928\_contig\_number\_5697SRS1055067\_contig\_number\_5204SRS140513\_contig\_number\_4496SRS143598\_contig\_number\_21418SRS144603\_contig\_number\_11370SRS146764\_contig\_number\_34018SRS147766\_contig\_number\_8839SRS149784\_contig\_number\_contig-100\_160.171722SRS893170\_contig\_number\_contig-100\_409.35049SRS893187\_contig\_number\_16057SRS893295\_contig\_number\_9854SRS893300\_contig\_number\_2744
39

4
genus
841

species
166486

SRS097920\_contig\_number\_21764
1


SRS142890\_contig\_number\_41569
1
species
301301

1

SRS893342\_contig\_number\_8802
species
360807


SRS104311\_contig\_number\_45457
1
species
2049040


SRS1055069\_contig\_number\_15282
1
2302969
species

1952105
species
1

SRS054352\_contig\_number\_contig-100\_234.134860

1
830
genus


SRS063190\_contig\_number\_237
1
45851
species


SRS013965\_contig\_number\_15010SRS019601\_contig\_number\_contig-100\_1236.170257SRS020869\_contig\_number\_36981SRS023715\_contig\_number\_1527SRS049959\_contig\_number\_37925SRS050752\_contig\_number\_contig-100\_1710.145753SRS055017\_contig\_number\_contig-100\_2998.211396SRS056273\_contig\_number\_31289SRS075078\_contig\_number\_32134SRS143070\_contig\_number\_18099SRS144537\_contig\_number\_47027SRS147271\_contig\_number\_contig-100\_1471.245590
12
species
2109691

species
39491
2

SRS024625\_contig\_number\_13629SRS148721\_contig\_number\_12344

2
186806
family

2
genus
1730

1262889
species

SRS052027\_contig\_number\_13309
1


SRS147346\_contig\_number\_66668
1
species
1262878

7
541000
family


SRS1041145\_contig\_number\_36463SRS142542\_contig\_number\_11479
2
species
1898205

species
39492

SRS014459\_contig\_number\_41399SRS053398\_contig\_number\_14304
2

genus
1263
3

1160721
species
1

SRS893373\_contig\_number\_21113

species
1637499

SRS050752\_contig\_number\_30430
1

species
41978
1

SRS017103\_contig\_number\_contig-100\_8.207609

1

SRS056259\_contig\_number\_29335
1898207
species

1262994
species
2

SRS045826\_contig\_number\_3754SRS053356\_contig\_number\_20696

species
1879010
1

SRS098571\_contig\_number\_contig-100\_35.353789

1
91061
class

1
186826
order

1
81852
family

genus
1350

SRS019685\_contig\_number\_39141
1
