# Supplementary material for: MetaRibo-Seq measures translation in microbiomes
Source: Nat Commun. 2020 Jun 29;11:3268. doi: 10.1038/s41467-020-17081-z (PMC7324362; doi:10.1038/s41467-020-17081-z)
Supplement: Supplementary file 10 — Supplementary Data 7 [file 41467_2020_17081_MOESM10_ESM.zip › File2/Confidence_VeryHigh_Taxonomy/357920_out.krona.html]

Javascript must be enabled to view this page.

members
magnitude
magnitudeUnassigned
count
unassigned
taxon
rank

357920\_out

8


SRS058145\_contig\_number\_contig-100\_26743.26744
1

superkingdom
7
2

phylum
976
7

7
200643
class

order
7
171549

family
815
4

816
4
genus

820
1

SRS013638\_contig\_number\_19798
species

species

SRS015579\_contig\_number\_17567SRS065397\_contig\_number\_10042SRS146813\_contig\_number\_15135
3
46506

family
171552
3

3
838
genus

60133
2
species

SRS012916\_contig\_number\_7574SRS019219\_contig\_number\_contig-100\_8616.8617

1703337
1
species

SRS023466\_contig\_number\_3185
