# Supplementary material for: MetaRibo-Seq measures translation in microbiomes
Source: Nat Commun. 2020 Jun 29;11:3268. doi: 10.1038/s41467-020-17081-z (PMC7324362; doi:10.1038/s41467-020-17081-z)
Supplement: Supplementary file 10 — Supplementary Data 7 [file 41467_2020_17081_MOESM10_ESM.zip › File2/Confidence_VeryHigh_Taxonomy/16547_out.krona.html]

Javascript must be enabled to view this page.

members
magnitude
magnitudeUnassigned
count
unassigned
taxon
rank

16547\_out

6

6
2
superkingdom

6
phylum
1239

186801
class
6

6
186802
order

family
186803
1

genus
189330
1

1
species
1263073

SRS064276\_contig\_number\_contig-100\_1265.225987

3
31979
family

3
580596
genus

SRS011302\_contig\_number\_contig-100\_9948.93512SRS075773\_contig\_number\_contig-100\_13143.136387SRS077127\_contig\_number\_contig-100\_6230.48740

1
1898207
species

SRS053356\_contig\_number\_45184

1
family
541000

1
216851
genus


SRS015264\_contig\_number\_contig-100\_3313.126038
853
species
1
