# Supplementary material for: MetaRibo-Seq measures translation in microbiomes
Source: Nat Commun. 2020 Jun 29;11:3268. doi: 10.1038/s41467-020-17081-z (PMC7324362; doi:10.1038/s41467-020-17081-z)
Supplement: Supplementary file 10 — Supplementary Data 7 [file 41467_2020_17081_MOESM10_ESM.zip › File2/Confidence_VeryHigh_Taxonomy/59812_out.krona.html]

Javascript must be enabled to view this page.

members
magnitude
magnitudeUnassigned
count
unassigned
taxon
rank

59812\_out

9

2
superkingdom
9

phylum
976
9

9
class
200643

171549
order
9

815
family
9


SRS015578\_contig\_number\_31305SRS098061\_contig\_number\_8990
genus
816
2
9


SRS013965\_contig\_number\_36243SRS024388\_contig\_number\_457SRS076804\_contig\_number\_20222SRS1041132\_contig\_number\_4216SRS104311\_contig\_number\_29320SRS1055095\_contig\_number\_2107SRS149075\_contig\_number\_12819
7
818
species
