# Supplementary material for: MetaRibo-Seq measures translation in microbiomes
Source: Nat Commun. 2020 Jun 29;11:3268. doi: 10.1038/s41467-020-17081-z (PMC7324362; doi:10.1038/s41467-020-17081-z)
Supplement: Supplementary file 10 — Supplementary Data 7 [file 41467_2020_17081_MOESM10_ESM.zip › File2/Confidence_VeryHigh_Taxonomy/380255_out.krona.html]

Javascript must be enabled to view this page.

members
magnitude
magnitudeUnassigned
count
unassigned
taxon
rank

380255\_out

20

2
20
superkingdom

20
1239
phylum

class
20
186801

186802
20
order

family
4
31979

genus
1485
3

2292996

SRS053356\_contig\_number\_43831
1
species

411489

SRS077231\_contig\_number\_23126SRS147346\_contig\_number\_62257
2
species

1898204
1

SRS077194\_contig\_number\_26750
species

1
541000
family

1
216851
genus

853
1

SRS148784\_contig\_number\_contig-100\_430.168518
species

family
15
186806

genus
15
1730

142586

SRS017821\_contig\_number\_16162SRS018623\_contig\_number\_12101SRS019068\_contig\_number\_104650SRS019496\_contig\_number\_8324SRS024663\_contig\_number\_contig-100\_281.98383SRS050925\_contig\_number\_19934SRS050998\_contig\_number\_16563SRS052697\_contig\_number\_contig-100\_671.259501SRS104693\_contig\_number\_12720SRS142923\_contig\_number\_14202SRS143148\_contig\_number\_8541SRS144135\_contig\_number\_36112SRS144362\_contig\_number\_24054SRS148159\_contig\_number\_52166SRS148784\_contig\_number\_28874
15
species
