# Supplementary material for: MetaRibo-Seq measures translation in microbiomes
Source: Nat Commun. 2020 Jun 29;11:3268. doi: 10.1038/s41467-020-17081-z (PMC7324362; doi:10.1038/s41467-020-17081-z)
Supplement: Supplementary file 10 — Supplementary Data 7 [file 41467_2020_17081_MOESM10_ESM.zip › File2/Confidence_VeryHigh_Taxonomy/346897_out.krona.html]

Javascript must be enabled to view this page.

members
magnitude
magnitudeUnassigned
count
unassigned
taxon
rank

346897\_out

7

7
superkingdom
2

7
phylum
1239

4
class
186801

186802
order
4


SRS014235\_contig\_number\_47531
1
172733
species


SRS064276\_contig\_number\_35005
1
1897046
species

541000
family
2

2

SRS014613\_contig\_number\_contig-100\_390.124090SRS144506\_contig\_number\_50178
species
2292180

3
909932
class

909929
order
3

1843491
family
3

genus
970
3

3

SRS022980\_contig\_number\_contig-100\_25492.51068SRS104165\_contig\_number\_contig-100\_1670.206969SRS143216\_contig\_number\_contig-100\_21368.21368
712535
species
