# Supplementary material for: MetaRibo-Seq measures translation in microbiomes
Source: Nat Commun. 2020 Jun 29;11:3268. doi: 10.1038/s41467-020-17081-z (PMC7324362; doi:10.1038/s41467-020-17081-z)
Supplement: Supplementary file 10 — Supplementary Data 7 [file 41467_2020_17081_MOESM10_ESM.zip › File2/Confidence_VeryHigh_Taxonomy/108695_out.krona.html]

Javascript must be enabled to view this page.

members
magnitude
magnitudeUnassigned
count
unassigned
taxon
rank

108695\_out

6

2
superkingdom
6

1239
phylum
6

1262989

SRS012273\_contig\_number\_contig-100\_6797.222178SRS022071\_contig\_number\_contig-100\_1052.1052SRS097889\_contig\_number\_25568SRS104636\_contig\_number\_28519SRS147139\_contig\_number\_42573
species
5

186801
class
1

1
order
186802

541000
family
1

1924105
genus
1


SRS014979\_contig\_number\_contig-100\_8594.102725
1776382
1
species
