# Supplementary material for: MetaRibo-Seq measures translation in microbiomes
Source: Nat Commun. 2020 Jun 29;11:3268. doi: 10.1038/s41467-020-17081-z (PMC7324362; doi:10.1038/s41467-020-17081-z)
Supplement: Supplementary file 10 — Supplementary Data 7 [file 41467_2020_17081_MOESM10_ESM.zip › File2/Confidence_VeryHigh_Taxonomy/357112_out.krona.html]

Javascript must be enabled to view this page.

members
magnitude
magnitudeUnassigned
count
unassigned
taxon
rank

357112\_out

20

superkingdom
20
2

phylum
1239
20

186801
20
class

order
20
186802

1
31979
family

1485
1
genus


SRS011134\_contig\_number\_48132
1
1262770
species

541000
12
family


SRS011239\_contig\_number\_18998SRS013940\_contig\_number\_19103SRS015431\_contig\_number\_84539SRS018984\_contig\_number\_14445SRS019068\_contig\_number\_75537SRS049896\_contig\_number\_32053SRS051031\_contig\_number\_contig-100\_165.199121SRS077294\_contig\_number\_19107SRS098073\_contig\_number\_1814SRS1041137\_contig\_number\_24313SRS144183\_contig\_number\_31620SRS149784\_contig\_number\_15056
1898205
12
species

species

SRS018936\_contig\_number\_160SRS019445\_contig\_number\_4966SRS024132\_contig\_number\_6346SRS049959\_contig\_number\_45027SRS062701\_contig\_number\_5347SRS1041144\_contig\_number\_contig-100\_851.34453
6
1897044

family
1
186803

1
841
genus

species
2293144
1

SRS065504\_contig\_number\_21276
