# Supplementary material for: MetaRibo-Seq measures translation in microbiomes
Source: Nat Commun. 2020 Jun 29;11:3268. doi: 10.1038/s41467-020-17081-z (PMC7324362; doi:10.1038/s41467-020-17081-z)
Supplement: Supplementary file 10 — Supplementary Data 7 [file 41467_2020_17081_MOESM10_ESM.zip › File2/Confidence_VeryHigh_Taxonomy/7174_out.krona.html]

Javascript must be enabled to view this page.

members
magnitude
magnitudeUnassigned
count
unassigned
taxon
rank

7174\_out

4

2
4
superkingdom

4
1239
phylum

class
4
91061

order
4
186826

4
1300
family

genus
4
1301


SRS016665\_contig\_number\_23292SRS019176\_contig\_number\_18666SRS075410\_contig\_number\_contig-100\_583.583SRS104653\_contig\_number\_1121
species
1305
4
