# Supplementary material for: MetaRibo-Seq measures translation in microbiomes
Source: Nat Commun. 2020 Jun 29;11:3268. doi: 10.1038/s41467-020-17081-z (PMC7324362; doi:10.1038/s41467-020-17081-z)
Supplement: Supplementary file 10 — Supplementary Data 7 [file 41467_2020_17081_MOESM10_ESM.zip › File2/Confidence_VeryHigh_Taxonomy/345547_out.krona.html]

Javascript must be enabled to view this page.

members
magnitude
magnitudeUnassigned
count
unassigned
taxon
rank

345547\_out

40

superkingdom
2
40

40
1239
phylum

40
186801
class

order
186802
40

1898207
2
species

SRS064757\_contig\_number\_13619SRS104400\_contig\_number\_43422

family
38
541000

genus
292632
38

5
2053618

SRS016203\_contig\_number\_contig-100\_1067.75489SRS020869\_contig\_number\_31629SRS024009\_contig\_number\_contig-100\_43.85666SRS049995\_contig\_number\_38051SRS971276\_contig\_number\_15394
species

species

SRS011134\_contig\_number\_contig-100\_1142.306979SRS011239\_contig\_number\_contig-100\_1053.173879SRS011405\_contig\_number\_contig-100\_1355.113897SRS012969\_contig\_number\_contig-100\_1185.238632SRS015431\_contig\_number\_82126SRS016335\_contig\_number\_contig-100\_1584.239105SRS019068\_contig\_number\_contig-100\_1540.608058SRS020233\_contig\_number\_contig-100\_1397.323740SRS020394\_contig\_number\_contig-100\_895.115359SRS022071\_contig\_number\_contig-100\_1131.229530SRS022609\_contig\_number\_contig-100\_1249.266078SRS023526\_contig\_number\_contig-100\_31110.114961SRS024132\_contig\_number\_17618SRS024435\_contig\_number\_contig-100\_1924.226424SRS048870\_contig\_number\_21642SRS049959\_contig\_number\_contig-100\_1292.264613SRS063190\_contig\_number\_contig-100\_914.100428SRS065504\_contig\_number\_37874SRS075984\_contig\_number\_24977SRS076804\_contig\_number\_contig-100\_990.132447SRS077024\_contig\_number\_12395SRS077849\_contig\_number\_contig-100\_1137.195059SRS078242\_contig\_number\_26353SRS098571\_contig\_number\_59260SRS098644\_contig\_number\_39916SRS1041039\_contig\_number\_contig-100\_1442.146667SRS1041137\_contig\_number\_contig-100\_1126.176547SRS104311\_contig\_number\_contig-100\_1240.267217SRS142503\_contig\_number\_contig-100\_4444.309216SRS142542\_contig\_number\_22777SRS143895\_contig\_number\_28867SRS144537\_contig\_number\_contig-100\_3629.270125SRS971275\_contig\_number\_contig-100\_827.278486
1262970
33
