# Supplementary material for: MetaRibo-Seq measures translation in microbiomes
Source: Nat Commun. 2020 Jun 29;11:3268. doi: 10.1038/s41467-020-17081-z (PMC7324362; doi:10.1038/s41467-020-17081-z)
Supplement: Supplementary file 10 — Supplementary Data 7 [file 41467_2020_17081_MOESM10_ESM.zip › File2/Confidence_VeryHigh_Taxonomy/155231_out.krona.html]

Javascript must be enabled to view this page.

members
magnitude
magnitudeUnassigned
count
unassigned
taxon
rank

155231\_out

5

5
2
superkingdom

1239
3
phylum

1
91061
class

order
1385
1

1
186817
family

1
1386
genus

86661
1

SRS075773\_contig\_number\_17311
species group

class
186801
2

186802
2
order

species

SRS020233\_contig\_number\_48369
384638
1

1
186803
family

1898203
1
species

SRS011084\_contig\_number\_36197

phylum
2
976

2
200643
class

order
171549
2

family
815
2

816
2
genus

1263051
1

SRS098644\_contig\_number\_contig-100\_460.279897
species

29523
1
species

SRS013098\_contig\_number\_contig-100\_885.292059
