# Supplementary material for: MetaRibo-Seq measures translation in microbiomes
Source: Nat Commun. 2020 Jun 29;11:3268. doi: 10.1038/s41467-020-17081-z (PMC7324362; doi:10.1038/s41467-020-17081-z)
Supplement: Supplementary file 10 — Supplementary Data 7 [file 41467_2020_17081_MOESM10_ESM.zip › File2/Confidence_VeryHigh_Taxonomy/58444_out.krona.html]

Javascript must be enabled to view this page.

members
magnitude
magnitudeUnassigned
count
unassigned
taxon
rank

58444\_out

60

60
2
superkingdom

phylum
1239
60

60
class
186801

60
186802
order

60

SRS012969\_contig\_number\_39071SRS013951\_contig\_number\_16687SRS015890\_contig\_number\_contig-100\_1707.164877SRS017191\_contig\_number\_10372SRS018984\_contig\_number\_32880SRS023526\_contig\_number\_32570SRS043411\_contig\_number\_contig-100\_912.109824SRS045645\_contig\_number\_contig-100\_77.183288SRS048981\_contig\_number\_contig-100\_28499.28500SRS055017\_contig\_number\_contig-100\_752.209150SRS075021\_contig\_number\_contig-100\_2509.34408SRS077849\_contig\_number\_contig-100\_13363.96165SRS097920\_contig\_number\_contig-100\_30137.116721SRS098571\_contig\_number\_contig-100\_13476.225461SRS101376\_contig\_number\_26830SRS104693\_contig\_number\_13362SRS142781\_contig\_number\_contig-100\_1932.92505SRS144135\_contig\_number\_contig-100\_7299.56489SRS144362\_contig\_number\_contig-100\_239.259889SRS144506\_contig\_number\_contig-100\_3218.315940SRS146812\_contig\_number\_64310SRS148091\_contig\_number\_contig-100\_3133.99899SRS149244\_contig\_number\_9568SRS893383\_contig\_number\_contig-100\_1020.193367
24
186803
family

39491
species
23

SRS013476\_contig\_number\_31574SRS015065\_contig\_number\_contig-100\_659.222085SRS015190\_contig\_number\_contig-100\_618.101551SRS015960\_contig\_number\_contig-100\_95.145027SRS018623\_contig\_number\_21108SRS019161\_contig\_number\_32939SRS019381\_contig\_number\_contig-100\_377.135300SRS019496\_contig\_number\_14814SRS020869\_contig\_number\_37639SRS024435\_contig\_number\_44599SRS044535\_contig\_number\_15733SRS050998\_contig\_number\_15618SRS064232\_contig\_number\_contig-100\_161.41961SRS075821\_contig\_number\_20808SRS076756\_contig\_number\_22427SRS077231\_contig\_number\_contig-100\_990.156530SRS104084\_contig\_number\_14759SRS105153\_contig\_number\_32630SRS1055076\_contig\_number\_contig-100\_1010.90299SRS143085\_contig\_number\_4312SRS146764\_contig\_number\_15647SRS147653\_contig\_number\_contig-100\_237.51370SRS148721\_contig\_number\_contig-100\_3175.315811

1

SRS018888\_contig\_number\_contig-100\_10964.10965
1898203
species

1

SRS893378\_contig\_number\_contig-100\_151.143169
12
genus
841

3

SRS046712\_contig\_number\_contig-100\_1341.33297SRS050941\_contig\_number\_17738SRS1055056\_contig\_number\_contig-100\_532.83372
301301
species

4

SRS017247\_contig\_number\_10637SRS017821\_contig\_number\_contig-100\_650.195550SRS053087\_contig\_number\_920SRS098644\_contig\_number\_contig-100\_3590.259800
species
166486


SRS024492\_contig\_number\_10549SRS1041118\_contig\_number\_1725
2
species
360807


SRS054905\_contig\_number\_contig-100\_5287.151739
1
species
2293144

species
1897016
1

SRS024075\_contig\_number\_4586
