# Supplementary material for: MetaRibo-Seq measures translation in microbiomes
Source: Nat Commun. 2020 Jun 29;11:3268. doi: 10.1038/s41467-020-17081-z (PMC7324362; doi:10.1038/s41467-020-17081-z)
Supplement: Supplementary file 10 — Supplementary Data 7 [file 41467_2020_17081_MOESM10_ESM.zip › File2/Confidence_VeryHigh_Taxonomy/138992_out.krona.html]

Javascript must be enabled to view this page.

members
magnitude
magnitudeUnassigned
count
unassigned
taxon
rank

138992\_out

6

6
superkingdom
2

6
phylum
1239

186801
class
6

186802
order
6

6
family
541000

genus
6
216851


SRS019030\_contig\_number\_contig-100\_19354.62961SRS075078\_contig\_number\_26555SRS075773\_contig\_number\_contig-100\_7851.191411SRS1041145\_contig\_number\_58SRS144135\_contig\_number\_30SRS146812\_contig\_number\_11
853
6
species
