# Supplementary material for: MetaRibo-Seq measures translation in microbiomes
Source: Nat Commun. 2020 Jun 29;11:3268. doi: 10.1038/s41467-020-17081-z (PMC7324362; doi:10.1038/s41467-020-17081-z)
Supplement: Supplementary file 10 — Supplementary Data 7 [file 41467_2020_17081_MOESM10_ESM.zip › File2/Confidence_VeryHigh_Taxonomy/203476_out.krona.html]

Javascript must be enabled to view this page.

members
magnitude
magnitudeUnassigned
count
unassigned
taxon
rank

203476\_out

6

2
superkingdom
6

1239
phylum
6

6
class
186801

6
186802
order

1
family
541000

species

SRS148784\_contig\_number\_14121
1898205
1

family
186803
5

genus
841
5

5
166486

SRS015431\_contig\_number\_86741SRS019068\_contig\_number\_contig-100\_3212.268088SRS058070\_contig\_number\_25911SRS075341\_contig\_number\_contig-100\_2108.160073SRS097920\_contig\_number\_contig-100\_732.193512
species
