# Supplementary material for: MetaRibo-Seq measures translation in microbiomes
Source: Nat Commun. 2020 Jun 29;11:3268. doi: 10.1038/s41467-020-17081-z (PMC7324362; doi:10.1038/s41467-020-17081-z)
Supplement: Supplementary file 10 — Supplementary Data 7 [file 41467_2020_17081_MOESM10_ESM.zip › File2/Confidence_VeryHigh_Taxonomy/136569_out.krona.html]

Javascript must be enabled to view this page.

members
magnitude
magnitudeUnassigned
count
unassigned
taxon
rank

136569\_out

6

6
superkingdom
2

976
phylum
6

200643
class
6

1
order
171549

SRS049959\_contig\_number\_contig-100\_27577.116094
6

5
family
171552

5
genus
838


SRS012849\_contig\_number\_33491
1
species
59823


SRS012969\_contig\_number\_1104SRS015794\_contig\_number\_contig-100\_500.86979SRS077849\_contig\_number\_4415
3
165179
species

1

SRS023526\_contig\_number\_12496
2292054
species
