# Supplementary material for: MetaRibo-Seq measures translation in microbiomes
Source: Nat Commun. 2020 Jun 29;11:3268. doi: 10.1038/s41467-020-17081-z (PMC7324362; doi:10.1038/s41467-020-17081-z)
Supplement: Supplementary file 10 — Supplementary Data 7 [file 41467_2020_17081_MOESM10_ESM.zip › File2/Confidence_VeryHigh_Taxonomy/369363_out.krona.html]

Javascript must be enabled to view this page.

members
magnitude
magnitudeUnassigned
count
unassigned
taxon
rank

369363\_out

12

12
superkingdom
2

12
phylum
1239

class
12
186801

186802
12
order

SRS019068\_contig\_number\_68369SRS023715\_contig\_number\_16013
2

family
4
186803

4
genus
572511

765821
species
4

SRS1041033\_contig\_number\_contig-100\_401.110036SRS1041091\_contig\_number\_1576SRS1054691\_contig\_number\_11353SRS148721\_contig\_number\_48283

541000
6
family

1263
genus
6

species
5
1816688

SRS015217\_contig\_number\_31893SRS015578\_contig\_number\_33048SRS017521\_contig\_number\_44444SRS146813\_contig\_number\_26682SRS148159\_contig\_number\_contig-100\_369.338378

species
1
2293174

SRS147766\_contig\_number\_14909
