# Supplementary material for: MetaRibo-Seq measures translation in microbiomes
Source: Nat Commun. 2020 Jun 29;11:3268. doi: 10.1038/s41467-020-17081-z (PMC7324362; doi:10.1038/s41467-020-17081-z)
Supplement: Supplementary file 10 — Supplementary Data 7 [file 41467_2020_17081_MOESM10_ESM.zip › File2/Confidence_VeryHigh_Taxonomy/113027_out.krona.html]

Javascript must be enabled to view this page.

members
magnitude
magnitudeUnassigned
count
unassigned
taxon
rank

113027\_out

8

superkingdom
2
7

7
phylum
1239

7
class
186801

186802
order
7

family
186803
1

33042
genus
1

410072

SRS077454\_contig\_number\_14573
species
1

186806
family
2

2
1730
genus


SRS050422\_contig\_number\_18453SRS1041038\_contig\_number\_2263
species
1262885
2

family
541000
4

4
1898205
species

SRS016381\_contig\_number\_contig-100\_20570.20571SRS077231\_contig\_number\_21815SRS140513\_contig\_number\_contig-100\_3198.3198SRS147346\_contig\_number\_17488

1
superkingdom
2759

4751
kingdom
1

1
subkingdom
451864

1
4890
phylum

147538
subphylum
1

147549
class
1

1
5185
order

1
family
5186

74855
genus
1

82571
species

SRS143722\_contig\_number\_contig-100\_7439.7440
1
