# Supplementary material for: MetaRibo-Seq measures translation in microbiomes
Source: Nat Commun. 2020 Jun 29;11:3268. doi: 10.1038/s41467-020-17081-z (PMC7324362; doi:10.1038/s41467-020-17081-z)
Supplement: Supplementary file 10 — Supplementary Data 7 [file 41467_2020_17081_MOESM10_ESM.zip › File2/Confidence_VeryHigh_Taxonomy/261729_out.krona.html]

Javascript must be enabled to view this page.

members
magnitude
magnitudeUnassigned
count
unassigned
taxon
rank

261729\_out

12

2
12
superkingdom

phylum
12
1239

12
186801
class

186802

SRS142503\_contig\_number\_contig-100\_22414.22414
order
12
1

1898207
1
species

SRS024435\_contig\_number\_31963

family
31979
10

10
1485
genus

1262813
1
species

SRS146812\_contig\_number\_769


SRS019685\_contig\_number\_37574SRS022524\_contig\_number\_3116SRS098571\_contig\_number\_contig-100\_22451.201885
species
3
1262841

1262810
6

SRS014235\_contig\_number\_15888SRS019685\_contig\_number\_42092SRS024132\_contig\_number\_7963SRS049995\_contig\_number\_30940SRS053356\_contig\_number\_37375SRS054059\_contig\_number\_contig-100\_37.45407
species
