# Supplementary material for: MetaRibo-Seq measures translation in microbiomes
Source: Nat Commun. 2020 Jun 29;11:3268. doi: 10.1038/s41467-020-17081-z (PMC7324362; doi:10.1038/s41467-020-17081-z)
Supplement: Supplementary file 10 — Supplementary Data 7 [file 41467_2020_17081_MOESM10_ESM.zip › File2/Confidence_VeryHigh_Taxonomy/180488_out.krona.html]

Javascript must be enabled to view this page.

members
magnitude
magnitudeUnassigned
count
unassigned
taxon
rank

180488\_out

6

6
superkingdom
2

6
phylum
1239

526524
class
6

order
6
526525

128827
6
family

1505663
6
genus

6
species
29348

SRS014313\_contig\_number\_5411SRS015217\_contig\_number\_contig-100\_41743.90216SRS016495\_contig\_number\_5862SRS018313\_contig\_number\_6387SRS1054691\_contig\_number\_contig-100\_31002.31003SRS146888\_contig\_number\_contig-100\_5180.5181
