# Supplementary material for: MetaRibo-Seq measures translation in microbiomes
Source: Nat Commun. 2020 Jun 29;11:3268. doi: 10.1038/s41467-020-17081-z (PMC7324362; doi:10.1038/s41467-020-17081-z)
Supplement: Supplementary file 10 — Supplementary Data 7 [file 41467_2020_17081_MOESM10_ESM.zip › File2/Confidence_VeryHigh_Taxonomy/200920_out.krona.html]

Javascript must be enabled to view this page.

members
magnitude
magnitudeUnassigned
count
unassigned
taxon
rank

200920\_out

16

superkingdom
2
16

phylum
1239
1
16

SRS012969\_contig\_number\_contig-100\_3622.245169

186801
15
class

15
186802
order

family
6
31979

6
1485
genus


SRS015890\_contig\_number\_22599SRS144362\_contig\_number\_43371
2
2293052
species

species

SRS043001\_contig\_number\_10142SRS050998\_contig\_number\_contig-100\_1438.109519
59620
2

species
1
1896990

SRS054905\_contig\_number\_4125

1
2293014

SRS098073\_contig\_number\_5843
species

family
2
216572

genus
2
459786

species

SRS013098\_contig\_number\_6898SRS1041037\_contig\_number\_17832
1945593
2

family
424536
1

1
270497
genus

species

SRS076804\_contig\_number\_contig-100\_2832.54029
1946247
1

186803
5
family


SRS019808\_contig\_number\_19386SRS098644\_contig\_number\_39154SRS1041133\_contig\_number\_contig-100\_1266.144904
3
1898203
species


SRS013940\_contig\_number\_22510
1
2109691
species

genus
841
1


SRS062654\_contig\_number\_contig-100\_6167.161127
1
360807
species

family
541000
1

1
946234
genus

species
1
1193534

SRS064232\_contig\_number\_contig-100\_269.38461
