# Supplementary material for: MetaRibo-Seq measures translation in microbiomes
Source: Nat Commun. 2020 Jun 29;11:3268. doi: 10.1038/s41467-020-17081-z (PMC7324362; doi:10.1038/s41467-020-17081-z)
Supplement: Supplementary file 10 — Supplementary Data 7 [file 41467_2020_17081_MOESM10_ESM.zip › File2/Confidence_VeryHigh_Taxonomy/333510_out.krona.html]

Javascript must be enabled to view this page.

members
magnitude
magnitudeUnassigned
count
unassigned
taxon
rank

333510\_out

6

2
superkingdom
6

phylum
6
976

class
6
200643

6
order
171549

171552
family
6

6

SRS012849\_contig\_number\_contig-100\_87.87
1
838
genus

species
1

SRS013521\_contig\_number\_1558
1262932

165179

SRS049959\_contig\_number\_41152
1
species

1
species

SRS012849\_contig\_number\_11727
1262930

2

SRS013521\_contig\_number\_321SRS049995\_contig\_number\_contig-100\_2449.206568
species
2293125
