# Supplementary material for: MetaRibo-Seq measures translation in microbiomes
Source: Nat Commun. 2020 Jun 29;11:3268. doi: 10.1038/s41467-020-17081-z (PMC7324362; doi:10.1038/s41467-020-17081-z)
Supplement: Supplementary file 10 — Supplementary Data 7 [file 41467_2020_17081_MOESM10_ESM.zip › File2/Confidence_VeryHigh_Taxonomy/96914_out.krona.html]

Javascript must be enabled to view this page.

members
magnitude
magnitudeUnassigned
count
unassigned
taxon
rank

96914\_out

4

superkingdom
2
4

phylum
1239
4

class
186801
4

4
order
186802

1
family
186806

genus
1730
1

39485
species
1

SRS149181\_contig\_number\_30137

1
family
186803

841
genus
1


SRS147766\_contig\_number\_19230
1
species
301301

31979
family
2

genus
1
2

SRS077194\_contig\_number\_contig-100\_18116.128270
1485

species
2293006

SRS076804\_contig\_number\_12688
1
