# Supplementary material for: MetaRibo-Seq measures translation in microbiomes
Source: Nat Commun. 2020 Jun 29;11:3268. doi: 10.1038/s41467-020-17081-z (PMC7324362; doi:10.1038/s41467-020-17081-z)
Supplement: Supplementary file 10 — Supplementary Data 7 [file 41467_2020_17081_MOESM10_ESM.zip › File2/Confidence_VeryHigh_Taxonomy/201563_out.krona.html]

Javascript must be enabled to view this page.

members
magnitude
magnitudeUnassigned
count
unassigned
taxon
rank

201563\_out

11

1
superkingdom
2

phylum
1224
1

28211
class
1

order
204457
1

1
family
41297

13687
genus
1

1

SRS075341\_contig\_number\_contig-100\_5653.130073
1380389
species

10
2759
superkingdom

10
4751
kingdom

10
451864
subkingdom

8
phylum
5204

subphylum
5302
8

8
class
155619

order
5303
7

7
2028212
family

7
5629
genus

7

SRS013687\_contig\_number\_25980SRS023914\_contig\_number\_contig-100\_656.165302SRS051031\_contig\_number\_37437SRS053356\_contig\_number\_contig-100\_2397.239103SRS076929\_contig\_number\_27037SRS077392\_contig\_number\_13226SRS1041145\_contig\_number\_15321
5630
species

452333
subclass
1

1
5338
order

930979
family
1

genus
71927
1

71929
species

SRS058070\_contig\_number\_contig-100\_1511.177241
1

phylum
4890

SRS015190\_contig\_number\_contig-100\_1184.102117SRS019685\_contig\_number\_11937
2
