# Supplementary material for: MetaRibo-Seq measures translation in microbiomes
Source: Nat Commun. 2020 Jun 29;11:3268. doi: 10.1038/s41467-020-17081-z (PMC7324362; doi:10.1038/s41467-020-17081-z)
Supplement: Supplementary file 10 — Supplementary Data 7 [file 41467_2020_17081_MOESM10_ESM.zip › File2/Confidence_VeryHigh_Taxonomy/279489_out.krona.html]

Javascript must be enabled to view this page.

members
magnitude
magnitudeUnassigned
count
unassigned
taxon
rank

279489\_out

2
11

SRS016740\_contig\_number\_contig-100\_3.153678SRS103971\_contig\_number\_77802

superkingdom
2759
2

kingdom
2
33208

phylum
7711
2

subphylum
89593
2

2
8287
superclass

class
40674
2

314146
2
superorder

2
9443
order

suborder
376913
2

2
314293
infraorder

2
9526
parvorder

314295
2
superfamily

2
9604
family

2
207598
subfamily

genus
2
9605


SRS019126\_contig\_number\_15390SRS019894\_contig\_number\_10606
species
9606
2

1
10239
superkingdom

family
1
549779

1
1977630
subfamily

genus
1
1977639


SRS143062\_contig\_number\_contig-100\_14.107541
species
2024608
1


SRS148150\_contig\_number\_19580
1

2
1

SRS019381\_contig\_number\_14838
superkingdom
5

phylum
1239
3

class
186801
3

1
186802
order

SRS019122\_contig\_number\_20688
3

family
1
424536

genus
1
270497

species

SRS105115\_contig\_number\_contig-100\_5.104766
1
1946256

186803
1
family

1
1952098
species

SRS013638\_contig\_number\_17916

1
976
phylum

SRS024441\_contig\_number\_18647
