# Supplementary material for: MetaRibo-Seq measures translation in microbiomes
Source: Nat Commun. 2020 Jun 29;11:3268. doi: 10.1038/s41467-020-17081-z (PMC7324362; doi:10.1038/s41467-020-17081-z)
Supplement: Supplementary file 10 — Supplementary Data 7 [file 41467_2020_17081_MOESM10_ESM.zip › File2/Confidence_VeryHigh_Taxonomy/134722_out.krona.html]

Javascript must be enabled to view this page.

members
magnitude
magnitudeUnassigned
count
unassigned
taxon
rank

134722\_out

34

34
superkingdom
2

1239
34
phylum

91061
class
34

186826
order
34

1300
34
family


SRS077216\_contig\_number\_21031
1301
1
genus
34

119603
1
species group

1334
species
1

1

SRS049988\_contig\_number\_contig-100\_5.60851
subspecies
99822

1263109

SRS100027\_contig\_number\_contig-100\_1687.110774
1
species

1739371

SRS015686\_contig\_number\_19776
1
species

1302

SRS047824\_contig\_number\_510
1
species

1739381
species
15

SRS014470\_contig\_number\_contig-100\_1218.162804SRS017279\_contig\_number\_contig-100\_301.69410SRS019270\_contig\_number\_contig-100\_236.92171SRS024081\_contig\_number\_14320SRS053603\_contig\_number\_contig-100\_332.132634SRS075404\_contig\_number\_38176SRS075765\_contig\_number\_contig-100\_1547.102278SRS076815\_contig\_number\_11499SRS077508\_contig\_number\_27201SRS098612\_contig\_number\_26122SRS098620\_contig\_number\_13934SRS104279\_contig\_number\_2726SRS149671\_contig\_number\_685SRS893324\_contig\_number\_12917SRS893348\_contig\_number\_contig-100\_1199.114802

species
13

SRS014404\_contig\_number\_1668SRS017324\_contig\_number\_17404SRS018591\_contig\_number\_contig-100\_1504.78925SRS018610\_contig\_number\_2892SRS024015\_contig\_number\_30059SRS075813\_contig\_number\_489SRS075870\_contig\_number\_35339SRS104087\_contig\_number\_18749SRS143062\_contig\_number\_11196SRS143231\_contig\_number\_11061SRS143354\_contig\_number\_10744SRS147541\_contig\_number\_contig-100\_381.154807SRS149078\_contig\_number\_16806
1304

species
1

SRS893335\_contig\_number\_10855
1310
