# Supplementary material for: MetaRibo-Seq measures translation in microbiomes
Source: Nat Commun. 2020 Jun 29;11:3268. doi: 10.1038/s41467-020-17081-z (PMC7324362; doi:10.1038/s41467-020-17081-z)
Supplement: Supplementary file 10 — Supplementary Data 7 [file 41467_2020_17081_MOESM10_ESM.zip › File2/Confidence_VeryHigh_Taxonomy/114680_out.krona.html]

Javascript must be enabled to view this page.

members
magnitude
magnitudeUnassigned
count
unassigned
taxon
rank

114680\_out

4

4
superkingdom
2

4
phylum
1224

4
28216
class

4
206351
order

4
family
481

genus
482
4

267212

SRS147128\_contig\_number\_13402
species
1

species
495
3

3
88719
subspecies

SRS018826\_contig\_number\_6890SRS063246\_contig\_number\_11376SRS147106\_contig\_number\_14315
