# Supplementary material for: MetaRibo-Seq measures translation in microbiomes
Source: Nat Commun. 2020 Jun 29;11:3268. doi: 10.1038/s41467-020-17081-z (PMC7324362; doi:10.1038/s41467-020-17081-z)
Supplement: Supplementary file 10 — Supplementary Data 7 [file 41467_2020_17081_MOESM10_ESM.zip › File2/Confidence_VeryHigh_Taxonomy/261758_out.krona.html]

Javascript must be enabled to view this page.

members
magnitude
magnitudeUnassigned
count
unassigned
taxon
rank

261758\_out

4

superkingdom
4
2157

phylum
4
28890

4
183925
class

4
2158
order

4
2159
family

4
2172
genus

4
2173

SRS014235\_contig\_number\_contig-100\_1585.187596SRS014923\_contig\_number\_43213SRS014979\_contig\_number\_30271SRS019068\_contig\_number\_7375
species
