# Supplementary material for: MetaRibo-Seq measures translation in microbiomes
Source: Nat Commun. 2020 Jun 29;11:3268. doi: 10.1038/s41467-020-17081-z (PMC7324362; doi:10.1038/s41467-020-17081-z)
Supplement: Supplementary file 10 — Supplementary Data 7 [file 41467_2020_17081_MOESM10_ESM.zip › File2/Confidence_VeryHigh_Taxonomy/200578_out.krona.html]

Javascript must be enabled to view this page.

members
magnitude
magnitudeUnassigned
count
unassigned
taxon
rank

200578\_out

34

34
superkingdom
2

1224
phylum
34

class
1236
34

order
91347
34

543
family
34

547
genus
2

539813
species
1

SRS1055050\_contig\_number\_contig-100\_792.793

1

SRS012849\_contig\_number\_27704
354276
species group

544
genus
1

1

SRS971427\_contig\_number\_4280
545
species

30
561
genus

species
562

SRS012273\_contig\_number\_42378SRS012902\_contig\_number\_contig-100\_2221.2221SRS016495\_contig\_number\_contig-100\_16353.89671SRS017521\_contig\_number\_31670SRS019068\_contig\_number\_28851SRS046502\_contig\_number\_contig-100\_15831.15832SRS049402\_contig\_number\_11763SRS050752\_contig\_number\_2291SRS057478\_contig\_number\_3964SRS064276\_contig\_number\_33693SRS075947\_contig\_number\_contig-100\_1145.71754SRS076876\_contig\_number\_contig-100\_958.959SRS077454\_contig\_number\_10256SRS098717\_contig\_number\_contig-100\_3232.3232SRS1041031\_contig\_number\_5942SRS1055043\_contig\_number\_contig-100\_9102.45795SRS1055050\_contig\_number\_contig-100\_1908.1909SRS140492\_contig\_number\_21026SRS142781\_contig\_number\_3735SRS142890\_contig\_number\_11187SRS143014\_contig\_number\_contig-100\_1491.1492SRS144219\_contig\_number\_contig-100\_869.870SRS144362\_contig\_number\_34676SRS146888\_contig\_number\_13112SRS146996\_contig\_number\_2134SRS147022\_contig\_number\_14089SRS147151\_contig\_number\_26646SRS147557\_contig\_number\_contig-100\_17686.115730SRS148159\_contig\_number\_contig-100\_1341.151973SRS971275\_contig\_number\_contig-100\_5041.149669
30

1
genus
570


SRS019068\_contig\_number\_contig-100\_17255.450503
1
species
548
