# Supplementary material for: MetaRibo-Seq measures translation in microbiomes
Source: Nat Commun. 2020 Jun 29;11:3268. doi: 10.1038/s41467-020-17081-z (PMC7324362; doi:10.1038/s41467-020-17081-z)
Supplement: Supplementary file 10 — Supplementary Data 7 [file 41467_2020_17081_MOESM10_ESM.zip › File2/Confidence_VeryHigh_Taxonomy/57330_out.krona.html]

Javascript must be enabled to view this page.

members
magnitude
magnitudeUnassigned
count
unassigned
taxon
rank

57330\_out

42

6
37
2
superkingdom

SRS011140\_contig\_number\_24962SRS019071\_contig\_number\_23265SRS048317\_contig\_number\_contig-100\_1445.83233SRS048791\_contig\_number\_contig-100\_2947.74693SRS053603\_contig\_number\_9675SRS058693\_contig\_number\_contig-100\_5160.38681

5
1224
phylum

class
5
28216

5
206351
order

family
481
5

482

SRS021897\_contig\_number\_contig-100\_3925.114711SRS143423\_contig\_number\_contig-100\_6457.100296SRS144512\_contig\_number\_contig-100\_715.276366SRS148877\_contig\_number\_13365SRS149671\_contig\_number\_contig-100\_3210.35099
5
genus

4
1752722
phylum

1953387

SRS011306\_contig\_number\_contig-100\_366.227927SRS018739\_contig\_number\_contig-100\_1866.1866.1866SRS019894\_contig\_number\_7762SRS142787\_contig\_number\_contig-100\_314.295232
4
species

phylum
22
1239

1
1737404
class

1
1737405
order

1
1737406
family

1
41273
genus

species
43131
1

SRS147281\_contig\_number\_5285

186801
21
class


SRS011115\_contig\_number\_38841SRS063288\_contig\_number\_contig-100\_290.140560
order
186802
21
2

17
186803
family

43996
8
genus

species

SRS015644\_contig\_number\_23467SRS019327\_contig\_number\_34762SRS022149\_contig\_number\_24403SRS022621\_contig\_number\_27771SRS024081\_contig\_number\_22802SRS104830\_contig\_number\_16692SRS143509\_contig\_number\_2573SRS149961\_contig\_number\_14918
8
43997

species

SRS013705\_contig\_number\_24634SRS016002\_contig\_number\_contig-100\_1331.177022SRS021496\_contig\_number\_contig-100\_417.98783.98783SRS021954\_contig\_number\_5860SRS022530\_contig\_number\_contig-100\_1212.132005.132005SRS042131\_contig\_number\_27478SRS057539\_contig\_number\_26269SRS063373\_contig\_number\_7827SRS147380\_contig\_number\_contig-100\_1022.150401
9
712991


SRS046496\_contig\_number\_3627SRS075947\_contig\_number\_18394
2
1897042
species


SRS017227\_contig\_number\_contig-100\_6974.184879SRS020226\_contig\_number\_9230SRS047113\_contig\_number\_contig-100\_320.90116SRS144621\_contig\_number\_55475SRS149286\_contig\_number\_contig-100\_9466.9467
5
