# Supplementary material for: MetaRibo-Seq measures translation in microbiomes
Source: Nat Commun. 2020 Jun 29;11:3268. doi: 10.1038/s41467-020-17081-z (PMC7324362; doi:10.1038/s41467-020-17081-z)
Supplement: Supplementary file 10 — Supplementary Data 7 [file 41467_2020_17081_MOESM10_ESM.zip › File2/Confidence_VeryHigh_Taxonomy/84182_out.krona.html]

Javascript must be enabled to view this page.

members
magnitude
magnitudeUnassigned
count
unassigned
taxon
rank

84182\_out

8

8
2
superkingdom

4
phylum
1239

4
186801
class

order
186802
4

4
family
186803

2039240
genus
4

160404
species

SRS015217\_contig\_number\_14340SRS015264\_contig\_number\_contig-100\_1567.96352SRS098717\_contig\_number\_15588SRS148721\_contig\_number\_40261
4

976
phylum
4

200643
class
4

4
171549
order

family
815
4

4
genus
816

species
29523
4

SRS013098\_contig\_number\_48414SRS020233\_contig\_number\_24067SRS062654\_contig\_number\_19536SRS098644\_contig\_number\_17908
