# Supplementary material for: MetaRibo-Seq measures translation in microbiomes
Source: Nat Commun. 2020 Jun 29;11:3268. doi: 10.1038/s41467-020-17081-z (PMC7324362; doi:10.1038/s41467-020-17081-z)
Supplement: Supplementary file 10 — Supplementary Data 7 [file 41467_2020_17081_MOESM10_ESM.zip › File2/Confidence_VeryHigh_Taxonomy/367933_out.krona.html]

Javascript must be enabled to view this page.

members
magnitude
magnitudeUnassigned
count
unassigned
taxon
rank

367933\_out

11

11
superkingdom
2

phylum
1224
11

1236
class
11

order
91347
11

11

SRS017191\_contig\_number\_contig-100\_1673.105197SRS971427\_contig\_number\_25433
2
543
family

genus
561
9

9

SRS012273\_contig\_number\_14207SRS017521\_contig\_number\_50827SRS019068\_contig\_number\_62939SRS049402\_contig\_number\_6365SRS1041031\_contig\_number\_2242SRS140492\_contig\_number\_28401SRS144362\_contig\_number\_43903SRS146888\_contig\_number\_15466SRS147022\_contig\_number\_14039
562
species
