# Supplementary material for: MetaRibo-Seq measures translation in microbiomes
Source: Nat Commun. 2020 Jun 29;11:3268. doi: 10.1038/s41467-020-17081-z (PMC7324362; doi:10.1038/s41467-020-17081-z)
Supplement: Supplementary file 10 — Supplementary Data 7 [file 41467_2020_17081_MOESM10_ESM.zip › File2/Confidence_VeryHigh_Taxonomy/211558_out.krona.html]

Javascript must be enabled to view this page.

members
magnitude
magnitudeUnassigned
count
unassigned
taxon
rank

211558\_out

4
1

SRS043667\_contig\_number\_contig-100\_189.35718

superkingdom
2
3

phylum
3
976

200643
3
class

3
171549
order

3
171552
family

genus
838
3

3
165179
species

SRS053356\_contig\_number\_43089SRS064757\_contig\_number\_14624SRS077641\_contig\_number\_5495
