# Supplementary material for: MetaRibo-Seq measures translation in microbiomes
Source: Nat Commun. 2020 Jun 29;11:3268. doi: 10.1038/s41467-020-17081-z (PMC7324362; doi:10.1038/s41467-020-17081-z)
Supplement: Supplementary file 10 — Supplementary Data 7 [file 41467_2020_17081_MOESM10_ESM.zip › File2/Confidence_VeryHigh_Taxonomy/10360_out.krona.html]

Javascript must be enabled to view this page.

members
magnitude
magnitudeUnassigned
count
unassigned
taxon
rank

10360\_out

29

superkingdom
2
29

1239
29
phylum

class
29
186801

order
186802
7
29

SRS013215\_contig\_number\_contig-100\_2671.2671SRS014287\_contig\_number\_contig-100\_3137.3137SRS015217\_contig\_number\_5394SRS024549\_contig\_number\_3600SRS104485\_contig\_number\_contig-100\_7643.7643SRS140645\_contig\_number\_contig-100\_3131.3131SRS149244\_contig\_number\_5676

541000
3
family

1263
2
genus

species
2
165186

SRS1041095\_contig\_number\_678SRS1041147\_contig\_number\_906

species

SRS148511\_contig\_number\_7922
1898205
1

186803
family
19

SRS015578\_contig\_number\_25100SRS098655\_contig\_number\_contig-100\_16322.16323
2

species

SRS023715\_contig\_number\_11126
658086
1

14
572511
genus

species

SRS012273\_contig\_number\_27836SRS012902\_contig\_number\_464SRS016267\_contig\_number\_3144SRS017521\_contig\_number\_28357SRS042284\_contig\_number\_3008SRS063518\_contig\_number\_23859SRS075398\_contig\_number\_contig-100\_3373.24134SRS077502\_contig\_number\_11978SRS097889\_contig\_number\_contig-100\_799.188697SRS1055043\_contig\_number\_24991SRS143780\_contig\_number\_14602SRS893253\_contig\_number\_534SRS893256\_contig\_number\_759SRS893259\_contig\_number\_1135
14
33038

1407607
1
genus


SRS019601\_contig\_number\_15862
1806509
1
species

1506577
1
genus

1
29361

SRS057717\_contig\_number\_6423
species
