# Supplementary material for: MetaRibo-Seq measures translation in microbiomes
Source: Nat Commun. 2020 Jun 29;11:3268. doi: 10.1038/s41467-020-17081-z (PMC7324362; doi:10.1038/s41467-020-17081-z)
Supplement: Supplementary file 10 — Supplementary Data 7 [file 41467_2020_17081_MOESM10_ESM.zip › File2/Confidence_VeryHigh_Taxonomy/46866_out.krona.html]

Javascript must be enabled to view this page.

members
magnitude
magnitudeUnassigned
count
unassigned
taxon
rank

46866\_out

4

2
superkingdom
4

phylum
1239
4

1
526524
class

order
526525
1

family
128827
1

1472649
genus
1

1

SRS023715\_contig\_number\_28236
species
1034346

3
186801
class

order
186802
3

3
186803

SRS148721\_contig\_number\_29484
family
1

572511
genus
1

species
2292985

SRS098644\_contig\_number\_19107
1

841
genus
1

species
2292368

SRS893279\_contig\_number\_contig-100\_3586.105641
1
