# Supplementary material for: MetaRibo-Seq measures translation in microbiomes
Source: Nat Commun. 2020 Jun 29;11:3268. doi: 10.1038/s41467-020-17081-z (PMC7324362; doi:10.1038/s41467-020-17081-z)
Supplement: Supplementary file 10 — Supplementary Data 7 [file 41467_2020_17081_MOESM10_ESM.zip › File2/Confidence_VeryHigh_Taxonomy/177882_out.krona.html]

Javascript must be enabled to view this page.

members
magnitude
magnitudeUnassigned
count
unassigned
taxon
rank

177882\_out

14

superkingdom
2
8

phylum
1239
8


SRS064276\_contig\_number\_34715
1263001
species
1

class
186801
3

order
186802
3


SRS023914\_contig\_number\_contig-100\_94.138090
1674842
species
1

1
species
1898207

SRS142503\_contig\_number\_37875

186803
family
1

1

SRS049959\_contig\_number\_42863
species
1952137

3
526524
class

order
526525
3

128827
family
3

3
1573534
genus

3

SRS016335\_contig\_number\_contig-100\_555.166600SRS019496\_contig\_number\_11489SRS058070\_contig\_number\_contig-100\_3349.150025
species
2015901

1879010
species

SRS011084\_contig\_number\_34581
1


SRS015190\_contig\_number\_7185SRS021484\_contig\_number\_contig-100\_7863.87929SRS022071\_contig\_number\_contig-100\_4885.128215SRS047014\_contig\_number\_4046SRS078242\_contig\_number\_contig-100\_33785.71406SRS098571\_contig\_number\_contig-100\_27447.68179
6
