# Supplementary material for: MetaRibo-Seq measures translation in microbiomes
Source: Nat Commun. 2020 Jun 29;11:3268. doi: 10.1038/s41467-020-17081-z (PMC7324362; doi:10.1038/s41467-020-17081-z)
Supplement: Supplementary file 10 — Supplementary Data 7 [file 41467_2020_17081_MOESM10_ESM.zip › File2/Confidence_VeryHigh_Taxonomy/108613_out.krona.html]

Javascript must be enabled to view this page.

members
magnitude
magnitudeUnassigned
count
unassigned
taxon
rank

108613\_out

20

20
superkingdom
2

1
1239

SRS022071\_contig\_number\_36759
phylum
19

1737404
class
1

1737405
order
1

1
family
1570339

genus
1
165779

1870984

SRS017916\_contig\_number\_7645
species
1

class
15
186801

15
order
186802

186803
1
family

1
genus
1407607


SRS012273\_contig\_number\_16160
1
species
1806509

family
14
31979

genus
14
1485

14
species

SRS012273\_contig\_number\_37345SRS012969\_contig\_number\_contig-100\_21280.148108SRS016335\_contig\_number\_3654SRS019968\_contig\_number\_16598SRS020328\_contig\_number\_9931SRS045645\_contig\_number\_31262SRS047014\_contig\_number\_8525SRS049446\_contig\_number\_12885SRS049995\_contig\_number\_23020SRS054352\_contig\_number\_23071SRS104197\_contig\_number\_4SRS147557\_contig\_number\_contig-100\_2011.49149SRS148196\_contig\_number\_5823SRS149325\_contig\_number\_8366
1262804

class
2
91061

1385
order
2

186817
1
family

1386
genus
1

1262708

SRS893300\_contig\_number\_9926
species
1

1
species

SRS013951\_contig\_number\_15623
1950268

1
phylum
201174

1760
class
1

2037
1
order

family
1
2049

1654
1
genus

712117

SRS018157\_contig\_number\_53708
species
1
