# Supplementary material for: MetaRibo-Seq measures translation in microbiomes
Source: Nat Commun. 2020 Jun 29;11:3268. doi: 10.1038/s41467-020-17081-z (PMC7324362; doi:10.1038/s41467-020-17081-z)
Supplement: Supplementary file 10 — Supplementary Data 7 [file 41467_2020_17081_MOESM10_ESM.zip › File2/Confidence_VeryHigh_Taxonomy/268540_out.krona.html]

Javascript must be enabled to view this page.

members
magnitude
magnitudeUnassigned
count
unassigned
taxon
rank

268540\_out

6

2
6
superkingdom

6
phylum
976

200643
class
6

171549
6
order

171552
family
6

genus
6
838

species
3

SRS044535\_contig\_number\_98SRS077641\_contig\_number\_1960SRS146764\_contig\_number\_93
1262937

1262930

SRS055017\_contig\_number\_3206SRS143070\_contig\_number\_11615SRS147271\_contig\_number\_12990
species
3
