# Supplementary material for: MetaRibo-Seq measures translation in microbiomes
Source: Nat Commun. 2020 Jun 29;11:3268. doi: 10.1038/s41467-020-17081-z (PMC7324362; doi:10.1038/s41467-020-17081-z)
Supplement: Supplementary file 10 — Supplementary Data 7 [file 41467_2020_17081_MOESM10_ESM.zip › File2/Confidence_VeryHigh_Taxonomy/2591_out.krona.html]

Javascript must be enabled to view this page.

members
magnitude
magnitudeUnassigned
count
unassigned
taxon
rank

2591\_out

15

15
2
superkingdom

1239
phylum
15

186801
class
15

15
order
186802

family
186803
15

15
841
genus

2
species

SRS054956\_contig\_number\_20SRS142542\_contig\_number\_12946
2049040

13
species
1897017

SRS013638\_contig\_number\_contig-100\_18342.54224SRS014923\_contig\_number\_4874SRS015578\_contig\_number\_contig-100\_1745.204681SRS016989\_contig\_number\_6401SRS019397\_contig\_number\_contig-100\_9173.44922SRS048060\_contig\_number\_8294SRS049900\_contig\_number\_14622SRS050925\_contig\_number\_contig-100\_2054.199751SRS058145\_contig\_number\_contig-100\_1559.1560SRS062701\_contig\_number\_contig-100\_3251.3252SRS144135\_contig\_number\_26798SRS147919\_contig\_number\_14114SRS148253\_contig\_number\_9956
