# Supplementary material for: MetaRibo-Seq measures translation in microbiomes
Source: Nat Commun. 2020 Jun 29;11:3268. doi: 10.1038/s41467-020-17081-z (PMC7324362; doi:10.1038/s41467-020-17081-z)
Supplement: Supplementary file 10 — Supplementary Data 7 [file 41467_2020_17081_MOESM10_ESM.zip › File2/Confidence_VeryHigh_Taxonomy/333407_out.krona.html]

Javascript must be enabled to view this page.

members
magnitude
magnitudeUnassigned
count
unassigned
taxon
rank

333407\_out

43

superkingdom
2
43

43
976
phylum

43
200643
class

order
43
171549

2005525
26
family

26

SRS023715\_contig\_number\_21004SRS024388\_contig\_number\_13502SRS101376\_contig\_number\_18260
3
375288
genus

species

SRS045004\_contig\_number\_31132SRS045645\_contig\_number\_34406SRS077194\_contig\_number\_71SRS1041132\_contig\_number\_2754
823
4

species
10
1262912

SRS012273\_contig\_number\_48758SRS012969\_contig\_number\_38305SRS018623\_contig\_number\_1881SRS020233\_contig\_number\_35225SRS022071\_contig\_number\_37415SRS023526\_contig\_number\_10944SRS023971\_contig\_number\_contig-100\_218.43980SRS057478\_contig\_number\_8262SRS144362\_contig\_number\_7991SRS148817\_contig\_number\_13090

9
469591

SRS022713\_contig\_number\_6380SRS075984\_contig\_number\_22104SRS077849\_contig\_number\_contig-100\_18.185787SRS1041144\_contig\_number\_4929SRS104327\_contig\_number\_contig-100\_4.50423SRS142542\_contig\_number\_16130SRS148253\_contig\_number\_12427SRS893230\_contig\_number\_2460SRS893383\_contig\_number\_1144
species

17
815
family

17
816
genus

2
1739298

SRS013638\_contig\_number\_contig-100\_39.127773SRS893341\_contig\_number\_3022
species

species
2302925
6

SRS018313\_contig\_number\_3649SRS053573\_contig\_number\_1230SRS056537\_contig\_number\_32250SRS074964\_contig\_number\_3725SRS143085\_contig\_number\_5385SRS148970\_contig\_number\_4164


SRS013687\_contig\_number\_13966SRS014235\_contig\_number\_43803SRS016203\_contig\_number\_10478SRS016541\_contig\_number\_contig-100\_603.90261SRS019685\_contig\_number\_28899SRS023914\_contig\_number\_contig-100\_68.156529SRS104400\_contig\_number\_11122SRS893366\_contig\_number\_17917
8
47678
species

1
329854

SRS016629\_contig\_number\_682
species
