# Supplementary material for: MetaRibo-Seq measures translation in microbiomes
Source: Nat Commun. 2020 Jun 29;11:3268. doi: 10.1038/s41467-020-17081-z (PMC7324362; doi:10.1038/s41467-020-17081-z)
Supplement: Supplementary file 10 — Supplementary Data 7 [file 41467_2020_17081_MOESM10_ESM.zip › File2/Confidence_VeryHigh_Taxonomy/2820_out.krona.html]

Javascript must be enabled to view this page.

members
magnitude
magnitudeUnassigned
count
unassigned
taxon
rank

2820\_out

5

2
superkingdom
5

1239
5
phylum

5
class
186801

order
5
186802

1
family
186806


SRS013687\_contig\_number\_contig-100\_9850.130563
1730
1
genus

186803
4
family

species
1
1952169

SRS104636\_contig\_number\_11586


SRS019161\_contig\_number\_contig-100\_770.228997
1408321
1
species

1952128

SRS098881\_contig\_number\_contig-100\_3223.3224
1
species


SRS015217\_contig\_number\_17666
1952109
species
1
