# Supplementary material for: MetaRibo-Seq measures translation in microbiomes
Source: Nat Commun. 2020 Jun 29;11:3268. doi: 10.1038/s41467-020-17081-z (PMC7324362; doi:10.1038/s41467-020-17081-z)
Supplement: Supplementary file 10 — Supplementary Data 7 [file 41467_2020_17081_MOESM10_ESM.zip › File2/Confidence_VeryHigh_Taxonomy/320767_out.krona.html]

Javascript must be enabled to view this page.

members
magnitude
magnitudeUnassigned
count
unassigned
taxon
rank

320767\_out

13

superkingdom
12
2

phylum
12
976

class
12
200643

171549
12
order

family
2005525
11

375288
11
genus


SRS013638\_contig\_number\_4856SRS075821\_contig\_number\_6102SRS147271\_contig\_number\_35518
3
2293117
species


SRS893253\_contig\_number\_contig-100\_57.25721
species
1262912
1


SRS016267\_contig\_number\_contig-100\_973.973SRS058145\_contig\_number\_3820SRS063489\_contig\_number\_contig-100\_15867.15867SRS1055069\_contig\_number\_contig-100\_847.85688SRS144714\_contig\_number\_25677SRS149244\_contig\_number\_9828SRS893256\_contig\_number\_2968
7
823
species

815
1
family

genus
816
1

SRS098571\_contig\_number\_24245

superkingdom
2759
1

kingdom
4751
1

subkingdom
451864
1

phylum
1
5204

subphylum
1
5302

1
155616
class

1051672
1
order

genus
1
71781

71782
1
species

SRS016629\_contig\_number\_contig-100\_5200.33069
