# Supplementary material for: MetaRibo-Seq measures translation in microbiomes
Source: Nat Commun. 2020 Jun 29;11:3268. doi: 10.1038/s41467-020-17081-z (PMC7324362; doi:10.1038/s41467-020-17081-z)
Supplement: Supplementary file 10 — Supplementary Data 7 [file 41467_2020_17081_MOESM10_ESM.zip › File2/Confidence_VeryHigh_Taxonomy/112780_out.krona.html]

Javascript must be enabled to view this page.

members
magnitude
magnitudeUnassigned
count
unassigned
taxon
rank

112780\_out

10

2

SRS014235\_contig\_number\_contig-100\_7270.171120SRS053214\_contig\_number\_contig-100\_9694.105912

superkingdom
8
2

1239
7
phylum

class
186801
7

order
186802
7

family
541000
7

1

SRS062701\_contig\_number\_contig-100\_9062.49062
genus
1263
7


SRS049995\_contig\_number\_contig-100\_16589.130947SRS148159\_contig\_number\_46188
species
2
165186

1947404
4
species

SRS016095\_contig\_number\_23240SRS049959\_contig\_number\_36881SRS143466\_contig\_number\_21917SRS144537\_contig\_number\_50357

phylum
1
57723

1
332159
class

order
1
332160


SRS142503\_contig\_number\_46971
1
2026791
species
