# Supplementary material for: MetaRibo-Seq measures translation in microbiomes
Source: Nat Commun. 2020 Jun 29;11:3268. doi: 10.1038/s41467-020-17081-z (PMC7324362; doi:10.1038/s41467-020-17081-z)
Supplement: Supplementary file 10 — Supplementary Data 7 [file 41467_2020_17081_MOESM10_ESM.zip › File2/Confidence_VeryHigh_Taxonomy/320913_out.krona.html]

Javascript must be enabled to view this page.

members
magnitude
magnitudeUnassigned
count
unassigned
taxon
rank

320913\_out

11

11
2
superkingdom

11
phylum
976

117743
class
11

11
order
200644

49546
family
11

11
genus
1016


SRS013711\_contig\_number\_contig-100\_20.20
1
species
327575


SRS015762\_contig\_number\_42748
1
species
1017

7

SRS013705\_contig\_number\_23253SRS018300\_contig\_number\_4634SRS020628\_contig\_number\_28344SRS023926\_contig\_number\_35583SRS049253\_contig\_number\_22116SRS097905\_contig\_number\_21163SRS143310\_contig\_number\_40920
45242
species


SRS893379\_contig\_number\_contig-100\_12775.50209
1
1227265
species


SRS1055073\_contig\_number\_16052
1
species
209053
