# Supplementary material for: MetaRibo-Seq measures translation in microbiomes
Source: Nat Commun. 2020 Jun 29;11:3268. doi: 10.1038/s41467-020-17081-z (PMC7324362; doi:10.1038/s41467-020-17081-z)
Supplement: Supplementary file 10 — Supplementary Data 7 [file 41467_2020_17081_MOESM10_ESM.zip › File2/Confidence_VeryHigh_Taxonomy/88376_out.krona.html]

Javascript must be enabled to view this page.

members
magnitude
magnitudeUnassigned
count
unassigned
taxon
rank

88376\_out

4

4
2
superkingdom

4
1239
phylum

91061
class
4

order
186826
4

family
1300
4

1301
genus
4

1
species
1306

SRS143450\_contig\_number\_16254

1
species
1335

SRS142787\_contig\_number\_contig-100\_15355.83407

1

SRS016742\_contig\_number\_contig-100\_29549.29549
1739371
species

1
671232
species group

1
species
1338

SRS043772\_contig\_number\_contig-100\_4903.32879
