# Supplementary material for: MetaRibo-Seq measures translation in microbiomes
Source: Nat Commun. 2020 Jun 29;11:3268. doi: 10.1038/s41467-020-17081-z (PMC7324362; doi:10.1038/s41467-020-17081-z)
Supplement: Supplementary file 10 — Supplementary Data 7 [file 41467_2020_17081_MOESM10_ESM.zip › File2/Confidence_VeryHigh_Taxonomy/61327_out.krona.html]

Javascript must be enabled to view this page.

members
magnitude
magnitudeUnassigned
count
unassigned
taxon
rank

61327\_out

16

16
superkingdom
2

16
phylum
1239

class
186801
16

16
order
186802


SRS074964\_contig\_number\_contig-100\_7898.7898SRS893230\_contig\_number\_5114
2
family
186803
16

genus
572511
12

33038

SRS015264\_contig\_number\_11723SRS018313\_contig\_number\_contig-100\_1778.76342SRS018623\_contig\_number\_19005SRS019286\_contig\_number\_contig-100\_1400.1401SRS024132\_contig\_number\_contig-100\_7664.212240SRS048164\_contig\_number\_16778SRS050998\_contig\_number\_contig-100\_582.91625SRS057717\_contig\_number\_7395SRS077552\_contig\_number\_25267SRS1041091\_contig\_number\_contig-100\_567.125594SRS1055076\_contig\_number\_contig-100\_11240.46943SRS144362\_contig\_number\_34038
species
12

39491
species

SRS104636\_contig\_number\_contig-100\_122.173483SRS147139\_contig\_number\_44611
2
