# Supplementary material for: MetaRibo-Seq measures translation in microbiomes
Source: Nat Commun. 2020 Jun 29;11:3268. doi: 10.1038/s41467-020-17081-z (PMC7324362; doi:10.1038/s41467-020-17081-z)
Supplement: Supplementary file 10 — Supplementary Data 7 [file 41467_2020_17081_MOESM10_ESM.zip › File2/Confidence_VeryHigh_Taxonomy/109127_out.krona.html]

Javascript must be enabled to view this page.

members
magnitude
magnitudeUnassigned
count
unassigned
taxon
rank

109127\_out

5

2
5
superkingdom

1239
phylum
5

186801
class
5

186802
order
5

541000
3
family

1
genus
1263

species
1

SRS047014\_contig\_number\_20443
41978

2
species
1898205

SRS013098\_contig\_number\_contig-100\_1614.111389SRS050752\_contig\_number\_3998

1897044

SRS024663\_contig\_number\_6913SRS098073\_contig\_number\_3145
2
species
