# Supplementary material for: MetaRibo-Seq measures translation in microbiomes
Source: Nat Commun. 2020 Jun 29;11:3268. doi: 10.1038/s41467-020-17081-z (PMC7324362; doi:10.1038/s41467-020-17081-z)
Supplement: Supplementary file 10 — Supplementary Data 7 [file 41467_2020_17081_MOESM10_ESM.zip › File2/Confidence_VeryHigh_Taxonomy/161937_out.krona.html]

Javascript must be enabled to view this page.

members
magnitude
magnitudeUnassigned
count
unassigned
taxon
rank

161937\_out

4

4
superkingdom
2

4
phylum
976

3
200643
class

3
171549
order

3
family
171552


SRS147386\_contig\_number\_15949
370804
1
species

838
1
genus

1
species
470565

SRS893365\_contig\_number\_2286

species
1
1852368

SRS148724\_contig\_number\_16291

1
species
652708

SRS018826\_contig\_number\_46479
