# Supplementary material for: MetaRibo-Seq measures translation in microbiomes
Source: Nat Commun. 2020 Jun 29;11:3268. doi: 10.1038/s41467-020-17081-z (PMC7324362; doi:10.1038/s41467-020-17081-z)
Supplement: Supplementary file 10 — Supplementary Data 7 [file 41467_2020_17081_MOESM10_ESM.zip › File2/Confidence_VeryHigh_Taxonomy/331460_out.krona.html]

Javascript must be enabled to view this page.

members
magnitude
magnitudeUnassigned
count
unassigned
taxon
rank

331460\_out

9

2
8
superkingdom

phylum
7
1239

class
7
186801

order
186802
7

5
186803
family

5

SRS020328\_contig\_number\_33069SRS058070\_contig\_number\_contig-100\_32113.70236SRS1055022\_contig\_number\_20026SRS149784\_contig\_number\_contig-100\_427.143588SRS893279\_contig\_number\_2424
39491
species

family
541000
1

genus
1
1263

species
1

SRS075773\_contig\_number\_34262
1262956

family
1
31979

2305245

SRS1041118\_contig\_number\_4856
1
species

phylum
1
201174

1
84998
class

84999
1
order

family
1
84107

102106
1
genus

species
2292320

SRS147557\_contig\_number\_contig-100\_34637.81775
1


SRS019068\_contig\_number\_34377
1
