# Supplementary material for: MetaRibo-Seq measures translation in microbiomes
Source: Nat Commun. 2020 Jun 29;11:3268. doi: 10.1038/s41467-020-17081-z (PMC7324362; doi:10.1038/s41467-020-17081-z)
Supplement: Supplementary file 10 — Supplementary Data 7 [file 41467_2020_17081_MOESM10_ESM.zip › File2/Confidence_VeryHigh_Taxonomy/38681_out.krona.html]

Javascript must be enabled to view this page.

members
magnitude
magnitudeUnassigned
count
unassigned
taxon
rank

38681\_out


SRS1055099\_contig\_number\_10639
1
13


SRS014287\_contig\_number\_contig-100\_23310.23310SRS019285\_contig\_number\_contig-100\_3791.3792SRS1055043\_contig\_number\_4222
3

2
superkingdom
9

1239
phylum
9


SRS893358\_contig\_number\_contig-100\_1126.78073
1262992
1
species

8
class
186801

186802
order
8

family
8
541000

1263
genus
5

species
3

SRS049773\_contig\_number\_11601SRS104084\_contig\_number\_contig-100\_7138.7139SRS104400\_contig\_number\_28597
40518


SRS143417\_contig\_number\_17664
2293183
1
species

species
1
165186

SRS1041145\_contig\_number\_contig-100\_596.204382

species
3
1898205

SRS049712\_contig\_number\_21827SRS098881\_contig\_number\_contig-100\_2396.159035SRS148721\_contig\_number\_54385
