# Supplementary material for: MetaRibo-Seq measures translation in microbiomes
Source: Nat Commun. 2020 Jun 29;11:3268. doi: 10.1038/s41467-020-17081-z (PMC7324362; doi:10.1038/s41467-020-17081-z)
Supplement: Supplementary file 10 — Supplementary Data 7 [file 41467_2020_17081_MOESM10_ESM.zip › File2/Confidence_VeryHigh_Taxonomy/124606_out.krona.html]

Javascript must be enabled to view this page.

members
magnitude
magnitudeUnassigned
count
unassigned
taxon
rank

124606\_out

6

superkingdom
6
2

phylum
1239
6

species

SRS064757\_contig\_number\_22631
1
1263002

5
186801
class

186802
5
order

31979
1
family

genus
1
1485

species

SRS076929\_contig\_number\_22299
1492
1

1
186803
family

genus
1
572511

1
418240

SRS016095\_contig\_number\_21102
species

family
216572
1

genus
1
459786

1945593
1
species

SRS148196\_contig\_number\_contig-100\_5628.218996

family
541000
1

genus
1
2048137

species

SRS104636\_contig\_number\_19756
39484
1

species

SRS146812\_contig\_number\_15984
1898207
1
