# Supplementary material for: MetaRibo-Seq measures translation in microbiomes
Source: Nat Commun. 2020 Jun 29;11:3268. doi: 10.1038/s41467-020-17081-z (PMC7324362; doi:10.1038/s41467-020-17081-z)
Supplement: Supplementary file 10 — Supplementary Data 7 [file 41467_2020_17081_MOESM10_ESM.zip › File2/Confidence_VeryHigh_Taxonomy/350357_out.krona.html]

Javascript must be enabled to view this page.

members
magnitude
magnitudeUnassigned
count
unassigned
taxon
rank

350357\_out

5

5
superkingdom
2

5
976
phylum

200643
class
5

order
171549
5

5
171552
family

5
838
genus

2

SRS022980\_contig\_number\_49310SRS023595\_contig\_number\_34241
species
189722

species
633701

SRS018665\_contig\_number\_15241SRS077922\_contig\_number\_contig-100\_919.110872SRS104539\_contig\_number\_26216
3
