# Supplementary material for: MetaRibo-Seq measures translation in microbiomes
Source: Nat Commun. 2020 Jun 29;11:3268. doi: 10.1038/s41467-020-17081-z (PMC7324362; doi:10.1038/s41467-020-17081-z)
Supplement: Supplementary file 10 — Supplementary Data 7 [file 41467_2020_17081_MOESM10_ESM.zip › File2/Confidence_VeryHigh_Taxonomy/6087_out.krona.html]

Javascript must be enabled to view this page.

members
magnitude
magnitudeUnassigned
count
unassigned
taxon
rank

6087\_out

13

superkingdom
2
13

phylum
1239
13

13
186801
class

13
186802
order

family
31979
13

13
genus
1485

3

SRS015694\_contig\_number\_24852SRS047014\_contig\_number\_contig-100\_1819.182019SRS098644\_contig\_number\_40251
species
2293015

1262777
species
1

SRS075773\_contig\_number\_37978

species
2293035
2

SRS075078\_contig\_number\_contig-100\_7.201775SRS075773\_contig\_number\_26807

1896990
species

SRS076756\_contig\_number\_21874
1

species
1896983

SRS024435\_contig\_number\_10163
1

1

SRS016954\_contig\_number\_11112
species
1262828


SRS015854\_contig\_number\_25041SRS016095\_contig\_number\_contig-100\_2282.148891
2
1262836
species

species
2293016

SRS097889\_contig\_number\_39943SRS143780\_contig\_number\_7502
2
