# Supplementary material for: MetaRibo-Seq measures translation in microbiomes
Source: Nat Commun. 2020 Jun 29;11:3268. doi: 10.1038/s41467-020-17081-z (PMC7324362; doi:10.1038/s41467-020-17081-z)
Supplement: Supplementary file 10 — Supplementary Data 7 [file 41467_2020_17081_MOESM10_ESM.zip › File2/Confidence_VeryHigh_Taxonomy/1404_out.krona.html]

Javascript must be enabled to view this page.

members
magnitude
magnitudeUnassigned
count
unassigned
taxon
rank

1404\_out

7

7
superkingdom
2

7
1239
phylum

186801
class
7

186802

SRS1054691\_contig\_number\_contig-100\_3435.99731
order
1
7

6
family
186803

genus
1407607
3

3
species

SRS012273\_contig\_number\_42872SRS098717\_contig\_number\_21218SRS148511\_contig\_number\_contig-100\_344.134097
1806509


SRS143991\_contig\_number\_contig-100\_2980.134211
658086
species
1

2
genus
1432051

species

SRS143780\_contig\_number\_12966SRS146888\_contig\_number\_10593
1720294
2
