# Supplementary material for: MetaRibo-Seq measures translation in microbiomes
Source: Nat Commun. 2020 Jun 29;11:3268. doi: 10.1038/s41467-020-17081-z (PMC7324362; doi:10.1038/s41467-020-17081-z)
Supplement: Supplementary file 10 — Supplementary Data 7 [file 41467_2020_17081_MOESM10_ESM.zip › File2/Confidence_VeryHigh_Taxonomy/59266_out.krona.html]

Javascript must be enabled to view this page.

members
magnitude
magnitudeUnassigned
count
unassigned
taxon
rank

59266\_out

1

SRS015190\_contig\_number\_2325
8

2
superkingdom
5

1
phylum
1239

186801
class
1

1
order
186802

family
1
186806

genus
1
1730

species
1

SRS013687\_contig\_number\_contig-100\_2499.2499
1897026

phylum
4
1224

1236
4
class

72273
order
4

4
family
135617

45247
genus
4

species
4

SRS023914\_contig\_number\_contig-100\_963.157424SRS051031\_contig\_number\_35679SRS053356\_contig\_number\_44354SRS1041145\_contig\_number\_contig-100\_674.190723
1247513

superkingdom
2
2759

4751
2
kingdom

451864

SRS076929\_contig\_number\_contig-100\_120.152123SRS1041145\_contig\_number\_contig-100\_376.196394
2
subkingdom
