# Supplementary material for: MetaRibo-Seq measures translation in microbiomes
Source: Nat Commun. 2020 Jun 29;11:3268. doi: 10.1038/s41467-020-17081-z (PMC7324362; doi:10.1038/s41467-020-17081-z)
Supplement: Supplementary file 10 — Supplementary Data 7 [file 41467_2020_17081_MOESM10_ESM.zip › File2/Confidence_VeryHigh_Taxonomy/329588_out.krona.html]

Javascript must be enabled to view this page.

members
magnitude
magnitudeUnassigned
count
unassigned
taxon
rank

329588\_out

24

superkingdom
24
2

1239
2

SRS013687\_contig\_number\_contig-100\_16214.16214SRS016954\_contig\_number\_contig-100\_14026.14026
phylum
24

9
species

SRS011084\_contig\_number\_2106SRS011239\_contig\_number\_21739SRS013476\_contig\_number\_40780SRS014459\_contig\_number\_29828SRS016495\_contig\_number\_contig-100\_1423.107610SRS017191\_contig\_number\_20661SRS017701\_contig\_number\_contig-100\_6120.6120SRS018656\_contig\_number\_contig-100\_4986.43417SRS018817\_contig\_number\_4527
1897036

1262994

SRS012969\_contig\_number\_25376SRS015065\_contig\_number\_30634SRS019068\_contig\_number\_60273
species
3


SRS017307\_contig\_number\_contig-100\_188.176524
2292899
1
species

9
class
186801

order
9
186802

family
5
186803

189330
genus
1

1262873

SRS043768\_contig\_number\_contig-100\_35508.86510
species
1

species
1
1952105

SRS104165\_contig\_number\_31928


SRS016018\_contig\_number\_17235SRS017916\_contig\_number\_11511
1898203
2
species

genus
1
33042

species
1
410072

SRS015782\_contig\_number\_41366

family
1
541000

1898205

SRS015431\_contig\_number\_16127
species
1

3
family
31979

genus
3
1485


SRS013951\_contig\_number\_34047SRS018836\_contig\_number\_15737SRS019161\_contig\_number\_contig-100\_2792.170752
59620
3
species
