# Supplementary material for: MetaRibo-Seq measures translation in microbiomes
Source: Nat Commun. 2020 Jun 29;11:3268. doi: 10.1038/s41467-020-17081-z (PMC7324362; doi:10.1038/s41467-020-17081-z)
Supplement: Supplementary file 10 — Supplementary Data 7 [file 41467_2020_17081_MOESM10_ESM.zip › File2/Confidence_VeryHigh_Taxonomy/30841_out.krona.html]

Javascript must be enabled to view this page.

members
magnitude
magnitudeUnassigned
count
unassigned
taxon
rank

30841\_out

48

superkingdom
2
48

976
48
phylum

class
48
200643

order
48
171549

family
48
815

816
genus

SRS019286\_contig\_number\_contig-100\_11229.11230SRS019808\_contig\_number\_contig-100\_31250.74964SRS049959\_contig\_number\_contig-100\_34711.34711SRS1055067\_contig\_number\_6329
48
4


SRS012273\_contig\_number\_11581
species
2292002
1


SRS016629\_contig\_number\_1360SRS019445\_contig\_number\_17304SRS147139\_contig\_number\_10341
species
1263049
3

329854
40
species

SRS011405\_contig\_number\_1361SRS013940\_contig\_number\_16254SRS014948\_contig\_number\_22575SRS014948\_contig\_number\_22575SRS015960\_contig\_number\_12628SRS015960\_contig\_number\_contig-100\_799.137106SRS018623\_contig\_number\_16627SRS018623\_contig\_number\_contig-100\_893.126394SRS018836\_contig\_number\_26695SRS024009\_contig\_number\_contig-100\_848.848SRS024435\_contig\_number\_contig-100\_11278.11278SRS045004\_contig\_number\_30004SRS045645\_contig\_number\_25637SRS045645\_contig\_number\_34166SRS049959\_contig\_number\_2791SRS049995\_contig\_number\_37685SRS049995\_contig\_number\_contig-100\_831.217897SRS050422\_contig\_number\_34928SRS050422\_contig\_number\_37054SRS056259\_contig\_number\_5307SRS056259\_contig\_number\_31346SRS056273\_contig\_number\_contig-100\_769.199773SRS075078\_contig\_number\_4366SRS076756\_contig\_number\_20815SRS076756\_contig\_number\_contig-100\_822.129432SRS076976\_contig\_number\_15605SRS078176\_contig\_number\_14553SRS101376\_contig\_number\_contig-100\_480.158958SRS104636\_contig\_number\_380SRS1055043\_contig\_number\_11473SRS1055099\_contig\_number\_2731SRS142599\_contig\_number\_429SRS144362\_contig\_number\_46055SRS144362\_contig\_number\_46055SRS144506\_contig\_number\_52838SRS144537\_contig\_number\_contig-100\_920.245389SRS147346\_contig\_number\_4950SRS148196\_contig\_number\_31928SRS148319\_contig\_number\_15053SRS893369\_contig\_number\_contig-100\_275.56309
