# Supplementary material for: MetaRibo-Seq measures translation in microbiomes
Source: Nat Commun. 2020 Jun 29;11:3268. doi: 10.1038/s41467-020-17081-z (PMC7324362; doi:10.1038/s41467-020-17081-z)
Supplement: Supplementary file 10 — Supplementary Data 7 [file 41467_2020_17081_MOESM10_ESM.zip › File2/Confidence_VeryHigh_Taxonomy/127672_out.krona.html]

Javascript must be enabled to view this page.

members
magnitude
magnitudeUnassigned
count
unassigned
taxon
rank

127672\_out

22

2759
2
superkingdom

kingdom
2
4751

451864
2
subkingdom

2
4890
phylum

147538
2
subphylum

class
147541
2

subclass
2
451867

134362
2
order

family
2
93133

genus
2
29002


SRS050801\_contig\_number\_contig-100\_3143.31190SRS893369\_contig\_number\_2264
species
29003
2

superkingdom
2
20

phylum
976
20

20
200643
class

order
20
171549

family
815
20

20
816
genus

18
817

SRS144135\_contig\_number\_contig-100\_269.191224SRS144362\_contig\_number\_10282SRS144506\_contig\_number\_55811SRS146812\_contig\_number\_60276SRS147022\_contig\_number\_126SRS147139\_contig\_number\_30548SRS147272\_contig\_number\_331SRS147377\_contig\_number\_11534SRS147653\_contig\_number\_1513SRS148091\_contig\_number\_6139SRS148159\_contig\_number\_37155SRS148784\_contig\_number\_contig-100\_5142.113454SRS149075\_contig\_number\_5392SRS893170\_contig\_number\_1715SRS893279\_contig\_number\_1912SRS893288\_contig\_number\_contig-100\_5157.34115SRS893342\_contig\_number\_4832SRS971275\_contig\_number\_25470
species

species

SRS147557\_contig\_number\_28885SRS148970\_contig\_number\_contig-100\_2007.2008
457392
2
