# Supplementary material for: MetaRibo-Seq measures translation in microbiomes
Source: Nat Commun. 2020 Jun 29;11:3268. doi: 10.1038/s41467-020-17081-z (PMC7324362; doi:10.1038/s41467-020-17081-z)
Supplement: Supplementary file 10 — Supplementary Data 7 [file 41467_2020_17081_MOESM10_ESM.zip › File2/Confidence_VeryHigh_Taxonomy/259696_out.krona.html]

Javascript must be enabled to view this page.

members
magnitude
magnitudeUnassigned
count
unassigned
taxon
rank

259696\_out

10

10
superkingdom
2

1239
10
phylum

186801
10
class

10
order
186802

31979
1
family

1649459
genus
1


SRS146888\_contig\_number\_15483
1
species
154046

family

SRS013158\_contig\_number\_4776
9
1
186803


SRS014979\_contig\_number\_32386
1
species
39491

1
genus
572511

2292983
species

SRS012273\_contig\_number\_29147
1

species
1

SRS014235\_contig\_number\_12839
1898203

4
genus
841

species
1

SRS011084\_contig\_number\_24948
301302

166486
3

SRS012273\_contig\_number\_48520SRS014287\_contig\_number\_3894SRS014855\_contig\_number\_750
species

1952153
species
1

SRS011084\_contig\_number\_18795
