# Supplementary material for: MetaRibo-Seq measures translation in microbiomes
Source: Nat Commun. 2020 Jun 29;11:3268. doi: 10.1038/s41467-020-17081-z (PMC7324362; doi:10.1038/s41467-020-17081-z)
Supplement: Supplementary file 10 — Supplementary Data 7 [file 41467_2020_17081_MOESM10_ESM.zip › File2/Confidence_VeryHigh_Taxonomy/139874_out.krona.html]

Javascript must be enabled to view this page.

members
magnitude
magnitudeUnassigned
count
unassigned
taxon
rank

139874\_out

30

30
superkingdom
2

976
phylum
30

class
200643
30

30
171549
order

171552
family
30

838
genus
30

28
species

SRS013521\_contig\_number\_543SRS013687\_contig\_number\_29946SRS014415\_contig\_number\_1563SRS015794\_contig\_number\_190SRS017307\_contig\_number\_13034SRS019285\_contig\_number\_195SRS023914\_contig\_number\_14151SRS024132\_contig\_number\_13174SRS043667\_contig\_number\_contig-100\_1460.42894SRS043841\_contig\_number\_2783SRS044535\_contig\_number\_contig-100\_4524.122890SRS045713\_contig\_number\_19129SRS046502\_contig\_number\_contig-100\_26262.26263SRS047433\_contig\_number\_14289SRS049896\_contig\_number\_5008SRS049995\_contig\_number\_8497SRS053356\_contig\_number\_31814SRS053398\_contig\_number\_contig-100\_1800.131111SRS054059\_contig\_number\_contig-100\_32244.32245SRS065397\_contig\_number\_8266SRS077641\_contig\_number\_21855SRS078176\_contig\_number\_contig-100\_1175.131053SRS078419\_contig\_number\_27593SRS103987\_contig\_number\_6930SRS1041116\_contig\_number\_7512SRS143181\_contig\_number\_10811SRS144603\_contig\_number\_contig-100\_1848.83270SRS146764\_contig\_number\_6725
165179

species

SRS104912\_contig\_number\_2128
1262926
1


SRS019808\_contig\_number\_17339
2292365
species
1
