# Supplementary material for: MetaRibo-Seq measures translation in microbiomes
Source: Nat Commun. 2020 Jun 29;11:3268. doi: 10.1038/s41467-020-17081-z (PMC7324362; doi:10.1038/s41467-020-17081-z)
Supplement: Supplementary file 10 — Supplementary Data 7 [file 41467_2020_17081_MOESM10_ESM.zip › File2/Confidence_VeryHigh_Taxonomy/357177_out.krona.html]

Javascript must be enabled to view this page.

members
magnitude
magnitudeUnassigned
count
unassigned
taxon
rank

357177\_out


SRS148777\_contig\_number\_12138
12
1

superkingdom
2
11

phylum
11
1224

1236
3
class

3
135615
order

family
868
3

genus
2717
3

species

SRS104968\_contig\_number\_12257SRS1055065\_contig\_number\_11634SRS143141\_contig\_number\_2894
2718
3

subphylum
68525
7

class
28221
7

7
213118
order

213121
7
family

genus
893
7

species
7
1986146

SRS011255\_contig\_number\_12396SRS013252\_contig\_number\_contig-100\_949.161777SRS014578\_contig\_number\_22808SRS016575\_contig\_number\_37576SRS022980\_contig\_number\_64993SRS1055048\_contig\_number\_14338SRS893328\_contig\_number\_contig-100\_3823.53765

class
1
28216

order
1
206351

481
1
family

genus
1
32257


SRS016746\_contig\_number\_1072
1
505
species
