# Supplementary material for: MetaRibo-Seq measures translation in microbiomes
Source: Nat Commun. 2020 Jun 29;11:3268. doi: 10.1038/s41467-020-17081-z (PMC7324362; doi:10.1038/s41467-020-17081-z)
Supplement: Supplementary file 10 — Supplementary Data 7 [file 41467_2020_17081_MOESM10_ESM.zip › File2/Confidence_VeryHigh_Taxonomy/177874_out.krona.html]

Javascript must be enabled to view this page.

members
magnitude
magnitudeUnassigned
count
unassigned
taxon
rank

177874\_out

18

2
superkingdom
18

1239
18
phylum

186801
18
class

186802
18
order

541000
family
18

genus
18
1263

1262955
5

SRS011084\_contig\_number\_32778SRS013476\_contig\_number\_contig-100\_3246.201382SRS020869\_contig\_number\_21953SRS052697\_contig\_number\_17614SRS1041145\_contig\_number\_26265
species

165186
species

SRS011134\_contig\_number\_28767SRS016335\_contig\_number\_41540SRS019496\_contig\_number\_16430SRS021484\_contig\_number\_27083SRS022137\_contig\_number\_contig-100\_604.128977SRS022609\_contig\_number\_contig-100\_2297.234168SRS046369\_contig\_number\_13243SRS075341\_contig\_number\_19490SRS075821\_contig\_number\_20775SRS075984\_contig\_number\_19168SRS098571\_contig\_number\_38433SRS1041092\_contig\_number\_2986SRS1055022\_contig\_number\_9243
13
