# Supplementary material for: MetaRibo-Seq measures translation in microbiomes
Source: Nat Commun. 2020 Jun 29;11:3268. doi: 10.1038/s41467-020-17081-z (PMC7324362; doi:10.1038/s41467-020-17081-z)
Supplement: Supplementary file 10 — Supplementary Data 7 [file 41467_2020_17081_MOESM10_ESM.zip › File2/Confidence_VeryHigh_Taxonomy/221023_out.krona.html]

Javascript must be enabled to view this page.

members
magnitude
magnitudeUnassigned
count
unassigned
taxon
rank

221023\_out

51

51
2
superkingdom

phylum
51
1239

class
186801
51

order
51
186802

family
541000
47

292632
44
genus

1262970
40
species

SRS011134\_contig\_number\_34799SRS011239\_contig\_number\_18322SRS012273\_contig\_number\_contig-100\_29837.150943SRS012969\_contig\_number\_2837SRS013965\_contig\_number\_33633SRS015431\_contig\_number\_69308SRS016203\_contig\_number\_9082SRS016335\_contig\_number\_28431SRS018817\_contig\_number\_198SRS018836\_contig\_number\_2256SRS019068\_contig\_number\_49682SRS019496\_contig\_number\_15244SRS020233\_contig\_number\_6823SRS020394\_contig\_number\_10113SRS020869\_contig\_number\_35083SRS022609\_contig\_number\_33956SRS048060\_contig\_number\_9519SRS048870\_contig\_number\_contig-100\_41.135983SRS049959\_contig\_number\_31175SRS049995\_contig\_number\_9597SRS052697\_contig\_number\_41893SRS063190\_contig\_number\_14681SRS064757\_contig\_number\_21816SRS075984\_contig\_number\_8227SRS076804\_contig\_number\_19430SRS077024\_contig\_number\_contig-100\_158.45867SRS077849\_contig\_number\_24399SRS078242\_contig\_number\_13528SRS098571\_contig\_number\_50992SRS1041137\_contig\_number\_3675SRS104311\_contig\_number\_29591SRS104400\_contig\_number\_13447SRS142503\_contig\_number\_15937SRS142542\_contig\_number\_22946SRS143895\_contig\_number\_22045SRS144537\_contig\_number\_22367SRS147557\_contig\_number\_18179SRS148817\_contig\_number\_13SRS971275\_contig\_number\_45734SRS971276\_contig\_number\_25282

2053618
4
species

SRS065504\_contig\_number\_21068SRS1041039\_contig\_number\_5621SRS104693\_contig\_number\_contig-100\_1603.1604SRS144183\_contig\_number\_19102

3
1263
genus

species

SRS022071\_contig\_number\_37334
2293149
1

species

SRS146812\_contig\_number\_49221
2293203
1


SRS098644\_contig\_number\_25728
species
1
41978

species

SRS011405\_contig\_number\_17644SRS024009\_contig\_number\_contig-100\_1161.73282SRS024435\_contig\_number\_contig-100\_952.203952SRS144135\_contig\_number\_23673
1898207
4
