# Supplementary material for: MetaRibo-Seq measures translation in microbiomes
Source: Nat Commun. 2020 Jun 29;11:3268. doi: 10.1038/s41467-020-17081-z (PMC7324362; doi:10.1038/s41467-020-17081-z)
Supplement: Supplementary file 10 — Supplementary Data 7 [file 41467_2020_17081_MOESM10_ESM.zip › File2/Confidence_VeryHigh_Taxonomy/165487_out.krona.html]

Javascript must be enabled to view this page.

members
magnitude
magnitudeUnassigned
count
unassigned
taxon
rank

165487\_out

5

5
2
superkingdom

32066
phylum
1

203490
class
1

1
order
203491

1
family
203492

848
genus
1

1
860
species

SRS052095\_contig\_number\_12603

976
phylum
3

class
200643
3

3
order
171549

1
2005525
2
family

SRS049318\_contig\_number\_contig-100\_9277.9277

375288
genus

SRS075821\_contig\_number\_757
1

171552
family
1

838
genus
1

1
species

SRS047113\_contig\_number\_58243
76123

1
phylum
1239

1
909932
class

1843488
order
1

1
family
909930

genus
33024
1

1
1262915

SRS1041145\_contig\_number\_contig-100\_44890.78855
species
