# Supplementary material for: MetaRibo-Seq measures translation in microbiomes
Source: Nat Commun. 2020 Jun 29;11:3268. doi: 10.1038/s41467-020-17081-z (PMC7324362; doi:10.1038/s41467-020-17081-z)
Supplement: Supplementary file 10 — Supplementary Data 7 [file 41467_2020_17081_MOESM10_ESM.zip › File2/Confidence_VeryHigh_Taxonomy/306641_out.krona.html]

Javascript must be enabled to view this page.

members
magnitude
magnitudeUnassigned
count
unassigned
taxon
rank

306641\_out

5

5
2
superkingdom

976
5
phylum

200643
5
class

171549
5
order

5
815
family

genus
5
816


SRS013382\_contig\_number\_contig-100\_1168.28525
species
29523
1


SRS017433\_contig\_number\_10383SRS017849\_contig\_number\_contig-100\_5614.5614SRS019178\_contig\_number\_5637SRS143070\_contig\_number\_4983
species
4
310297
