# Supplementary material for: MetaRibo-Seq measures translation in microbiomes
Source: Nat Commun. 2020 Jun 29;11:3268. doi: 10.1038/s41467-020-17081-z (PMC7324362; doi:10.1038/s41467-020-17081-z)
Supplement: Supplementary file 10 — Supplementary Data 7 [file 41467_2020_17081_MOESM10_ESM.zip › File2/Confidence_VeryHigh_Taxonomy/132820_out.krona.html]

Javascript must be enabled to view this page.

members
magnitude
magnitudeUnassigned
count
unassigned
taxon
rank

132820\_out

65


SRS017916\_contig\_number\_contig-100\_29136.29136
1

64
superkingdom
2

64
1239
phylum

186801
class
64

64
order
186802

1
family
216572

1
459786
genus

1945593
species

SRS147425\_contig\_number\_7251
1


SRS015217\_contig\_number\_contig-100\_2232.175910SRS1055043\_contig\_number\_contig-100\_7316.134514
2
1898207
species


SRS053335\_contig\_number\_contig-100\_5398.43961
1
1950906
species

31979
family
4

1485
genus
4


SRS022713\_contig\_number\_7288SRS058770\_contig\_number\_contig-100\_9105.86809SRS146888\_contig\_number\_3251SRS147377\_contig\_number\_contig-100\_15770.57223
4
1776383
species

541000
9
family

SRS018656\_contig\_number\_contig-100\_27342.27342SRS022713\_contig\_number\_contig-100\_14612.36247SRS049900\_contig\_number\_contig-100\_6084.32770SRS058770\_contig\_number\_contig-100\_1142.140541SRS077730\_contig\_number\_13236SRS078665\_contig\_number\_contig-100\_18605.18605SRS104400\_contig\_number\_14047SRS149244\_contig\_number\_contig-100\_17150.17151SRS893358\_contig\_number\_4203
55

2
genus
244127

2292191
species

SRS013098\_contig\_number\_917
1


SRS149879\_contig\_number\_27397
1
1262703
species


SRS013965\_contig\_number\_9291SRS018936\_contig\_number\_234SRS098644\_contig\_number\_contig-100\_6419.180017SRS146764\_contig\_number\_contig-100\_19453.73554
17
genus
292632
4

2302962
species

SRS049773\_contig\_number\_22557
1


SRS012273\_contig\_number\_41997SRS098717\_contig\_number\_20744
2
2302963
species

3

SRS053214\_contig\_number\_contig-100\_1045.1046SRS057478\_contig\_number\_8994SRS1041036\_contig\_number\_977
species
2302960

2302959
species

SRS017521\_contig\_number\_38088SRS143991\_contig\_number\_23612
2

species
665956
5

SRS013940\_contig\_number\_4370SRS016132\_contig\_number\_contig-100\_6585.6586SRS1041147\_contig\_number\_contig-100\_1819.1820SRS1054691\_contig\_number\_13144SRS143466\_contig\_number\_24576

1
genus
216851

1

SRS023715\_contig\_number\_17674
species
1946504

11
1905344
genus

11

SRS013687\_contig\_number\_5637SRS019601\_contig\_number\_contig-100\_7212.130505SRS020328\_contig\_number\_23796SRS064276\_contig\_number\_contig-100\_10081.105759SRS104636\_contig\_number\_15303SRS143598\_contig\_number\_contig-100\_3657.3657SRS143876\_contig\_number\_16300SRS147977\_contig\_number\_contig-100\_14881.54937SRS148196\_contig\_number\_contig-100\_4598.197215SRS893256\_contig\_number\_contig-100\_5596.5597SRS893279\_contig\_number\_10811
species
1550024


SRS013951\_contig\_number\_contig-100\_6965.6965SRS014736\_contig\_number\_5071SRS015217\_contig\_number\_27349SRS016495\_contig\_number\_contig-100\_2241.75559SRS051610\_contig\_number\_contig-100\_3002.41095SRS056519\_contig\_number\_5443SRS077294\_contig\_number\_contig-100\_28284.78708SRS097889\_contig\_number\_17689SRS098655\_contig\_number\_10407SRS1055099\_contig\_number\_contig-100\_2365.70732SRS142599\_contig\_number\_contig-100\_897.94681SRS143342\_contig\_number\_12784SRS144362\_contig\_number\_27597SRS147022\_contig\_number\_contig-100\_31074.31074
14
1627893
species

1

SRS014736\_contig\_number\_contig-100\_16896.16897
species
1898205

family
186806
1

1
genus
1730


SRS149879\_contig\_number\_24507
1
1638785
species
