# Supplementary material for: MetaRibo-Seq measures translation in microbiomes
Source: Nat Commun. 2020 Jun 29;11:3268. doi: 10.1038/s41467-020-17081-z (PMC7324362; doi:10.1038/s41467-020-17081-z)
Supplement: Supplementary file 10 — Supplementary Data 7 [file 41467_2020_17081_MOESM10_ESM.zip › File2/Confidence_VeryHigh_Taxonomy/20875_out.krona.html]

Javascript must be enabled to view this page.

members
magnitude
magnitudeUnassigned
count
unassigned
taxon
rank

20875\_out

4

superkingdom
4
2

1224
1
phylum

class
28211
1

order
204458
1

family
1
76892

genus
41275
1


SRS104400\_contig\_number\_46860
species
1
1946138

phylum
32066
1

class
203490
1

order
1
203491

1
1129771
family

32067
1
genus

712359
1
species

SRS104704\_contig\_number\_11768

phylum
1239
2

species
1
1897035

SRS893366\_contig\_number\_12959

186801
1
class

1
186802
order

1898207
1
species

SRS104400\_contig\_number\_34224
