# Supplementary material for: MetaRibo-Seq measures translation in microbiomes
Source: Nat Commun. 2020 Jun 29;11:3268. doi: 10.1038/s41467-020-17081-z (PMC7324362; doi:10.1038/s41467-020-17081-z)
Supplement: Supplementary file 10 — Supplementary Data 7 [file 41467_2020_17081_MOESM10_ESM.zip › File2/Confidence_VeryHigh_Taxonomy/309430_out.krona.html]

Javascript must be enabled to view this page.

members
magnitude
magnitudeUnassigned
count
unassigned
taxon
rank

309430\_out

7

7
superkingdom
2

7
phylum
1239

3
class
909932

order
3
909929

1843491
family
3

genus
3
970

species
3

SRS017227\_contig\_number\_38399SRS143762\_contig\_number\_10337SRS148472\_contig\_number\_44560
69823

4
class
186801

186802
4
order

family
4
541000

216851
4
genus

species
4

SRS023914\_contig\_number\_19278SRS1041037\_contig\_number\_12241SRS147977\_contig\_number\_9492SRS148874\_contig\_number\_4415
853
