# Supplementary material for: MetaRibo-Seq measures translation in microbiomes
Source: Nat Commun. 2020 Jun 29;11:3268. doi: 10.1038/s41467-020-17081-z (PMC7324362; doi:10.1038/s41467-020-17081-z)
Supplement: Supplementary file 10 — Supplementary Data 7 [file 41467_2020_17081_MOESM10_ESM.zip › File2/Confidence_VeryHigh_Taxonomy/42869_out.krona.html]

Javascript must be enabled to view this page.

members
magnitude
magnitudeUnassigned
count
unassigned
taxon
rank

42869\_out

6

1
2759
superkingdom

4751
kingdom
1

subkingdom
451864
1

1
phylum
5204

452284
subphylum
1

class
1538075
1

order
162474
1

family
742845
1

55193
genus
1

55194
species
1

SRS058070\_contig\_number\_19479


SRS053356\_contig\_number\_23921SRS075341\_contig\_number\_14495
2

superkingdom
2
3

3
1224
phylum

3
class
28211

1
1895711
species

SRS098571\_contig\_number\_49651

2
356
order

SRS051031\_contig\_number\_31611SRS1041145\_contig\_number\_24409
