# Supplementary material for: MetaRibo-Seq measures translation in microbiomes
Source: Nat Commun. 2020 Jun 29;11:3268. doi: 10.1038/s41467-020-17081-z (PMC7324362; doi:10.1038/s41467-020-17081-z)
Supplement: Supplementary file 10 — Supplementary Data 7 [file 41467_2020_17081_MOESM10_ESM.zip › File2/Confidence_VeryHigh_Taxonomy/117282_out.krona.html]

Javascript must be enabled to view this page.

members
magnitude
magnitudeUnassigned
count
unassigned
taxon
rank

117282\_out

5

2
superkingdom
5

976
phylum
1

1
200643
class

171549
order
1

family
815
1

genus
816
1

1

SRS024388\_contig\_number\_3760
246787
species

2
phylum
1224

1
1236
class

order
135625
1

1
712
family

genus
724
1


SRS057022\_contig\_number\_10829
729
species
1

28216
class
1

1
order
80840

1
80864
family

219181
genus
1

1
2045302
species

SRS015803\_contig\_number\_6653

1
95818
phylum


SRS022536\_contig\_number\_5904
2080848
species
1

1
phylum
1239

186801
class
1

186802
order
1

186803
family
1

genus
43996
1

1

SRS047113\_contig\_number\_contig-100\_4500.4500
species
43997
