# Supplementary material for: MetaRibo-Seq measures translation in microbiomes
Source: Nat Commun. 2020 Jun 29;11:3268. doi: 10.1038/s41467-020-17081-z (PMC7324362; doi:10.1038/s41467-020-17081-z)
Supplement: Supplementary file 10 — Supplementary Data 7 [file 41467_2020_17081_MOESM10_ESM.zip › File2/Confidence_VeryHigh_Taxonomy/369026_out.krona.html]

Javascript must be enabled to view this page.

members
magnitude
magnitudeUnassigned
count
unassigned
taxon
rank

369026\_out

5

superkingdom
2
3

phylum
2
1239

2
186801
class

2
186802
order

family
541000
2

genus
2
1263


SRS014613\_contig\_number\_3949SRS019161\_contig\_number\_39697
species
2
1637499

phylum
1
1224

subphylum
1
68525

class
28221
1

1
29
order

suborder
80812
1

1055686
1
family

1
1055688
genus

species

SRS105153\_contig\_number\_contig-100\_536.178092
927083
1

2

SRS143417\_contig\_number\_contig-100\_19093.62081SRS147425\_contig\_number\_contig-100\_6655.6656
