# Supplementary material for: MetaRibo-Seq measures translation in microbiomes
Source: Nat Commun. 2020 Jun 29;11:3268. doi: 10.1038/s41467-020-17081-z (PMC7324362; doi:10.1038/s41467-020-17081-z)
Supplement: Supplementary file 10 — Supplementary Data 7 [file 41467_2020_17081_MOESM10_ESM.zip › File2/Confidence_VeryHigh_Taxonomy/36465_out.krona.html]

Javascript must be enabled to view this page.

members
magnitude
magnitudeUnassigned
count
unassigned
taxon
rank

36465\_out

13


SRS017381\_contig\_number\_7922SRS1055023\_contig\_number\_9143
2


SRS057244\_contig\_number\_11856
10
1
2
superkingdom

phylum
7
1239

class
6
186801

6
order
186802

family
5
186803

265975
2
genus

237576

SRS024081\_contig\_number\_18985
1
species


SRS058336\_contig\_number\_1018
species
1
1501332

1792306

SRS017209\_contig\_number\_11807SRS050669\_contig\_number\_20560SRS062714\_contig\_number\_8523
3
species

543314
1
family

143393
1
species

SRS044373\_contig\_number\_17081

91061
class
1

1
order
186826

33958
family
1

1578

SRS146767\_contig\_number\_33158
1
genus

1948807

SRS144014\_contig\_number\_10737
1
species

2044938

SRS016575\_contig\_number\_47176
1
species

2759
1
superkingdom

1
kingdom
4751

451864
subkingdom
1

phylum
1
4890

1
subphylum
147537

4891
1
class

4892
order
1

34353
family
1

1
genus
4951

4952

SRS056622\_contig\_number\_contig-100\_6674.6674
1
species
