# Supplementary material for: MetaRibo-Seq measures translation in microbiomes
Source: Nat Commun. 2020 Jun 29;11:3268. doi: 10.1038/s41467-020-17081-z (PMC7324362; doi:10.1038/s41467-020-17081-z)
Supplement: Supplementary file 10 — Supplementary Data 7 [file 41467_2020_17081_MOESM10_ESM.zip › File2/Confidence_VeryHigh_Taxonomy/61351_out.krona.html]

Javascript must be enabled to view this page.

members
magnitude
magnitudeUnassigned
count
unassigned
taxon
rank

61351\_out

15

2
9
superkingdom

phylum

SRS147346\_contig\_number\_68291
1239
1
7


SRS049712\_contig\_number\_18617
1263021
species
1

186801
2
class

order
2
186802

31979
family
1

1485
1
genus

59620

SRS016095\_contig\_number\_2196
1
species

1898207

SRS076929\_contig\_number\_contig-100\_0.147826
1
species

2292889

SRS053214\_contig\_number\_22525SRS077730\_contig\_number\_contig-100\_17.118122
species
2

species
1
2292896

SRS047014\_contig\_number\_35330

1
phylum
976

class
1
200643

171549
1
order

171551
1
family

1952611

SRS049896\_contig\_number\_contig-100\_19414.95231
1
species

1224

SRS147766\_contig\_number\_23277
phylum
1

6

SRS015264\_contig\_number\_contig-100\_9806.46213SRS019397\_contig\_number\_2145SRS043411\_contig\_number\_6162SRS077194\_contig\_number\_8050SRS104036\_contig\_number\_2599SRS143181\_contig\_number\_contig-100\_18953.63682
