# Supplementary material for: MetaRibo-Seq measures translation in microbiomes
Source: Nat Commun. 2020 Jun 29;11:3268. doi: 10.1038/s41467-020-17081-z (PMC7324362; doi:10.1038/s41467-020-17081-z)
Supplement: Supplementary file 10 — Supplementary Data 7 [file 41467_2020_17081_MOESM10_ESM.zip › File2/Confidence_VeryHigh_Taxonomy/111318_out.krona.html]

Javascript must be enabled to view this page.

members
magnitude
magnitudeUnassigned
count
unassigned
taxon
rank

111318\_out

27

superkingdom
2
27

1239
27
phylum

class
186801
27

order
186802
27

family
186806
27

genus
27
1730

142586

SRS015217\_contig\_number\_4151SRS017307\_contig\_number\_7544SRS075773\_contig\_number\_contig-100\_10945.161845SRS148721\_contig\_number\_24249
4
species

23

SRS013476\_contig\_number\_36711SRS013687\_contig\_number\_contig-100\_6118.146092SRS014459\_contig\_number\_20719SRS014979\_contig\_number\_34489SRS014979\_contig\_number\_35401SRS015065\_contig\_number\_13391SRS015578\_contig\_number\_35192SRS017191\_contig\_number\_3604SRS023914\_contig\_number\_24836SRS024132\_contig\_number\_45005SRS053398\_contig\_number\_9911SRS054956\_contig\_number\_2861SRS058723\_contig\_number\_3735SRS062654\_contig\_number\_contig-100\_19876.100347SRS063040\_contig\_number\_33076SRS077335\_contig\_number\_1028SRS077641\_contig\_number\_7706SRS098571\_contig\_number\_43709SRS098644\_contig\_number\_40824SRS143417\_contig\_number\_26682SRS144714\_contig\_number\_18162SRS147919\_contig\_number\_20059SRS147977\_contig\_number\_10657
39485
species
