# Supplementary material for: MetaRibo-Seq measures translation in microbiomes
Source: Nat Commun. 2020 Jun 29;11:3268. doi: 10.1038/s41467-020-17081-z (PMC7324362; doi:10.1038/s41467-020-17081-z)
Supplement: Supplementary file 10 — Supplementary Data 7 [file 41467_2020_17081_MOESM10_ESM.zip › File2/Confidence_VeryHigh_Taxonomy/226394_out.krona.html]

Javascript must be enabled to view this page.

members
magnitude
magnitudeUnassigned
count
unassigned
taxon
rank

226394\_out

19

2
19
superkingdom

1239
2
phylum

2
186801
class

order
2
186802

541000
1
family

species
1898205
1

SRS077194\_contig\_number\_contig-100\_1995.146931

species

SRS019068\_contig\_number\_8874
1
1950842

phylum
1224
17

28216
17
class

17
80840
order

family
17
995019

2
40544

SRS075716\_contig\_number\_contig-100\_1211.36267SRS142781\_contig\_number\_7053
16
genus

species
8

SRS018313\_contig\_number\_contig-100\_4221.66980SRS074964\_contig\_number\_2975SRS077454\_contig\_number\_contig-100\_3128.94678SRS1041038\_contig\_number\_8668SRS1041136\_contig\_number\_24578SRS143722\_contig\_number\_contig-100\_1071.77104SRS147039\_contig\_number\_contig-100\_2959.2960SRS893253\_contig\_number\_contig-100\_25.25689
1262975


SRS012849\_contig\_number\_5960SRS013521\_contig\_number\_12679SRS048164\_contig\_number\_30679SRS049896\_contig\_number\_19318SRS078176\_contig\_number\_20664
5
1262976
species

1

SRS1041037\_contig\_number\_13740
1981025
species

1
1980697
genus

1
1852381

SRS049995\_contig\_number\_13112
species
