# Supplementary material for: MetaRibo-Seq measures translation in microbiomes
Source: Nat Commun. 2020 Jun 29;11:3268. doi: 10.1038/s41467-020-17081-z (PMC7324362; doi:10.1038/s41467-020-17081-z)
Supplement: Supplementary file 10 — Supplementary Data 7 [file 41467_2020_17081_MOESM10_ESM.zip › File2/Confidence_VeryHigh_Taxonomy/228830_out.krona.html]

Javascript must be enabled to view this page.

members
magnitude
magnitudeUnassigned
count
unassigned
taxon
rank

228830\_out

5

superkingdom
5

SRS053356\_contig\_number\_9570SRS1041145\_contig\_number\_31671
2
2

976
phylum
1

class
200643
1

1
171549
order

1
171552
family

838
genus
1

1

SRS019685\_contig\_number\_33911
species
1384078

1224
phylum
2

1
1236
2

SRS098571\_contig\_number\_contig-100\_1342.360538
class

1
order
135614

1

SRS023914\_contig\_number\_23472
family
32033
