# Supplementary material for: MetaRibo-Seq measures translation in microbiomes
Source: Nat Commun. 2020 Jun 29;11:3268. doi: 10.1038/s41467-020-17081-z (PMC7324362; doi:10.1038/s41467-020-17081-z)
Supplement: Supplementary file 10 — Supplementary Data 7 [file 41467_2020_17081_MOESM10_ESM.zip › File2/Confidence_VeryHigh_Taxonomy/283193_out.krona.html]

Javascript must be enabled to view this page.

members
magnitude
magnitudeUnassigned
count
unassigned
taxon
rank

283193\_out

13

superkingdom
2
13

1239
phylum
13

186801
class
13

186802
order
13

family
186803
13

11
genus
572511

species
2292970

SRS143342\_contig\_number\_12158
1

species
33038

SRS048870\_contig\_number\_22688SRS048870\_contig\_number\_22688SRS055982\_contig\_number\_13533SRS076804\_contig\_number\_18896SRS076804\_contig\_number\_18896SRS076804\_contig\_number\_18896SRS104485\_contig\_number\_4467SRS104485\_contig\_number\_4467SRS104485\_contig\_number\_4467SRS147346\_contig\_number\_43033
10

39491
species
2

SRS104311\_contig\_number\_20815SRS147614\_contig\_number\_4774
