# Supplementary material for: MetaRibo-Seq measures translation in microbiomes
Source: Nat Commun. 2020 Jun 29;11:3268. doi: 10.1038/s41467-020-17081-z (PMC7324362; doi:10.1038/s41467-020-17081-z)
Supplement: Supplementary file 10 — Supplementary Data 7 [file 41467_2020_17081_MOESM10_ESM.zip › File2/Confidence_VeryHigh_Taxonomy/290893_out.krona.html]

Javascript must be enabled to view this page.

members
magnitude
magnitudeUnassigned
count
unassigned
taxon
rank

290893\_out

8

7
2
superkingdom

976
phylum
7

200643
class
7

7
171549
order

7
2005525
family

7
195950
genus

species
712710

SRS011126\_contig\_number\_51715SRS022077\_contig\_number\_29195SRS022143\_contig\_number\_45448SRS049318\_contig\_number\_71631SRS063603\_contig\_number\_contig-100\_761.233447SRS077245\_contig\_number\_36145SRS143885\_contig\_number\_52531
7

1

SRS052876\_contig\_number\_20875
