# Supplementary material for: MetaRibo-Seq measures translation in microbiomes
Source: Nat Commun. 2020 Jun 29;11:3268. doi: 10.1038/s41467-020-17081-z (PMC7324362; doi:10.1038/s41467-020-17081-z)
Supplement: Supplementary file 10 — Supplementary Data 7 [file 41467_2020_17081_MOESM10_ESM.zip › File2/Confidence_VeryHigh_Taxonomy/179940_out.krona.html]

Javascript must be enabled to view this page.

members
magnitude
magnitudeUnassigned
count
unassigned
taxon
rank

179940\_out

6

6
2
superkingdom

phylum
976
6

200643
6
class

6
171549
order

family
171552
6

838
6
genus

5
165179

SRS013800\_contig\_number\_11250SRS017307\_contig\_number\_31716SRS024435\_contig\_number\_contig-100\_561.248452SRS054059\_contig\_number\_1730SRS075773\_contig\_number\_31541
species


SRS077024\_contig\_number\_3584
species
1
59823
