# Supplementary material for: MetaRibo-Seq measures translation in microbiomes
Source: Nat Commun. 2020 Jun 29;11:3268. doi: 10.1038/s41467-020-17081-z (PMC7324362; doi:10.1038/s41467-020-17081-z)
Supplement: Supplementary file 10 — Supplementary Data 7 [file 41467_2020_17081_MOESM10_ESM.zip › File2/Confidence_VeryHigh_Taxonomy/144698_out.krona.html]

Javascript must be enabled to view this page.

members
magnitude
magnitudeUnassigned
count
unassigned
taxon
rank

144698\_out

5

2
superkingdom
5

phylum
5
74201

203494
class
5

48461
5
order

1647988
family
5

1

SRS1055069\_contig\_number\_4359
239934
genus
5

species
4

SRS064276\_contig\_number\_672SRS104636\_contig\_number\_310SRS147139\_contig\_number\_41737SRS893373\_contig\_number\_20243
239935
