# Supplementary material for: MetaRibo-Seq measures translation in microbiomes
Source: Nat Commun. 2020 Jun 29;11:3268. doi: 10.1038/s41467-020-17081-z (PMC7324362; doi:10.1038/s41467-020-17081-z)
Supplement: Supplementary file 10 — Supplementary Data 7 [file 41467_2020_17081_MOESM10_ESM.zip › File2/Confidence_VeryHigh_Taxonomy/7470_out.krona.html]

Javascript must be enabled to view this page.

members
magnitude
magnitudeUnassigned
count
unassigned
taxon
rank

7470\_out

15

superkingdom
2
15

15
1239
phylum

526524
6
class

526525
6
order

128827
6
family

genus
6
135858

1
1946309

SRS019068\_contig\_number\_contig-100\_169.655540
species

1262767
5
species

SRS011134\_contig\_number\_48143SRS014683\_contig\_number\_18396SRS049712\_contig\_number\_18612SRS143876\_contig\_number\_19276SRS148253\_contig\_number\_13458

9
186801
class

order
9
186802

2
186803
family

genus
2
207244

2
649756

SRS077552\_contig\_number\_contig-100\_6277.120377SRS148196\_contig\_number\_39753
species

family
186806
7

1730
7
genus

165185
7
species

SRS017103\_contig\_number\_12043SRS049900\_contig\_number\_5580SRS077454\_contig\_number\_contig-100\_8977.82410SRS1041033\_contig\_number\_29833SRS104636\_contig\_number\_contig-100\_2373.94227SRS144183\_contig\_number\_contig-100\_29115.29116SRS148721\_contig\_number\_contig-100\_9009.266653
