# Supplementary material for: MetaRibo-Seq measures translation in microbiomes
Source: Nat Commun. 2020 Jun 29;11:3268. doi: 10.1038/s41467-020-17081-z (PMC7324362; doi:10.1038/s41467-020-17081-z)
Supplement: Supplementary file 10 — Supplementary Data 7 [file 41467_2020_17081_MOESM10_ESM.zip › File2/Confidence_VeryHigh_Taxonomy/135068_out.krona.html]

Javascript must be enabled to view this page.

members
magnitude
magnitudeUnassigned
count
unassigned
taxon
rank

135068\_out

8

7
2
superkingdom

1239
7
phylum

186801
7
class

186802
order

SRS014613\_contig\_number\_22281
1
7

3
31979
family

3
1485
genus


SRS043701\_contig\_number\_1175
species
1
59620

2
1263063

SRS044535\_contig\_number\_3609SRS147272\_contig\_number\_contig-100\_5565.5566
species

1
1950887

SRS015694\_contig\_number\_contig-100\_1.137914
species

186804
2
family

1501226
2
genus

species

SRS015854\_contig\_number\_20943SRS042284\_contig\_number\_contig-100\_2459.50433
2
1776391


SRS017821\_contig\_number\_673
1
