# Supplementary material for: MetaRibo-Seq measures translation in microbiomes
Source: Nat Commun. 2020 Jun 29;11:3268. doi: 10.1038/s41467-020-17081-z (PMC7324362; doi:10.1038/s41467-020-17081-z)
Supplement: Supplementary file 10 — Supplementary Data 7 [file 41467_2020_17081_MOESM10_ESM.zip › File2/Confidence_VeryHigh_Taxonomy/355655_out.krona.html]

Javascript must be enabled to view this page.

members
magnitude
magnitudeUnassigned
count
unassigned
taxon
rank

355655\_out

5

superkingdom
2
5

5
1239
phylum

class
186801
5

186802
order
5

5
541000
family

216851
genus
5

5

SRS147346\_contig\_number\_60154SRS147919\_contig\_number\_979SRS148721\_contig\_number\_1966SRS149075\_contig\_number\_5246SRS893378\_contig\_number\_11908
853
species
