# Supplementary material for: MetaRibo-Seq measures translation in microbiomes
Source: Nat Commun. 2020 Jun 29;11:3268. doi: 10.1038/s41467-020-17081-z (PMC7324362; doi:10.1038/s41467-020-17081-z)
Supplement: Supplementary file 10 — Supplementary Data 7 [file 41467_2020_17081_MOESM10_ESM.zip › File2/Confidence_VeryHigh_Taxonomy/165856_out.krona.html]

Javascript must be enabled to view this page.

members
magnitude
magnitudeUnassigned
count
unassigned
taxon
rank

165856\_out

6

superkingdom
2
4

4
phylum
1239

4
class
186801

order
186802
4

family
541000
1

1

SRS023583\_contig\_number\_14459
1898205
species

1897045
species

SRS049446\_contig\_number\_8159SRS098073\_contig\_number\_13870SRS149325\_contig\_number\_contig-100\_118.110252
3


SRS014459\_contig\_number\_contig-100\_31800.31801SRS045739\_contig\_number\_contig-100\_27872.27872
2
