# Supplementary material for: MetaRibo-Seq measures translation in microbiomes
Source: Nat Commun. 2020 Jun 29;11:3268. doi: 10.1038/s41467-020-17081-z (PMC7324362; doi:10.1038/s41467-020-17081-z)
Supplement: Supplementary file 10 — Supplementary Data 7 [file 41467_2020_17081_MOESM10_ESM.zip › File2/Confidence_VeryHigh_Taxonomy/415302_out.krona.html]

Javascript must be enabled to view this page.

members
magnitude
magnitudeUnassigned
count
unassigned
taxon
rank

415302\_out

4

4
2
superkingdom

1239
phylum
4


SRS893373\_contig\_number\_14275SRS893383\_contig\_number\_28805SRS971276\_contig\_number\_6521
3
1263007
species

1

SRS077392\_contig\_number\_21479
species
1897031
