# Supplementary material for: MetaRibo-Seq measures translation in microbiomes
Source: Nat Commun. 2020 Jun 29;11:3268. doi: 10.1038/s41467-020-17081-z (PMC7324362; doi:10.1038/s41467-020-17081-z)
Supplement: Supplementary file 10 — Supplementary Data 7 [file 41467_2020_17081_MOESM10_ESM.zip › File2/Confidence_VeryHigh_Taxonomy/135418_out.krona.html]

Javascript must be enabled to view this page.

members
magnitude
magnitudeUnassigned
count
unassigned
taxon
rank

135418\_out

34

2
34
superkingdom

phylum
34
1239

class
186801
34

34
186802
order

186803
34
family

species
39491
34

SRS014979\_contig\_number\_29727SRS015782\_contig\_number\_47520SRS016095\_contig\_number\_contig-100\_19086.98589SRS016495\_contig\_number\_contig-100\_6876.101484SRS017433\_contig\_number\_11074SRS018313\_contig\_number\_contig-100\_302.86456SRS018575\_contig\_number\_contig-100\_18798.47821SRS018984\_contig\_number\_contig-80\_119.244985SRS019068\_contig\_number\_1781SRS022524\_contig\_number\_contig-100\_62.86081SRS049446\_contig\_number\_14320SRS050026\_contig\_number\_contig-100\_100.72757SRS050752\_contig\_number\_19582SRS062701\_contig\_number\_contig-100\_2526.63896SRS063040\_contig\_number\_49103SRS064276\_contig\_number\_42927SRS065504\_contig\_number\_contig-100\_373.226592SRS074964\_contig\_number\_contig-100\_923.55325SRS076929\_contig\_number\_contig-100\_42246.88242SRS078242\_contig\_number\_contig-100\_44664.82285SRS078665\_contig\_number\_contig-100\_2337.2337SRS097920\_contig\_number\_21967SRS098717\_contig\_number\_21313SRS1041157\_contig\_number\_contig-100\_12887.12888SRS1054716\_contig\_number\_14790SRS1055034\_contig\_number\_9270SRS1055069\_contig\_number\_9612SRS143722\_contig\_number\_contig-100\_9438.70610SRS144183\_contig\_number\_contig-100\_633.219594SRS147022\_contig\_number\_contig-100\_331.93865SRS148784\_contig\_number\_contig-100\_8847.8848SRS149181\_contig\_number\_31889SRS149325\_contig\_number\_16720SRS893373\_contig\_number\_contig-100\_4579.4580
