# Supplementary material for: MetaRibo-Seq measures translation in microbiomes
Source: Nat Commun. 2020 Jun 29;11:3268. doi: 10.1038/s41467-020-17081-z (PMC7324362; doi:10.1038/s41467-020-17081-z)
Supplement: Supplementary file 10 — Supplementary Data 7 [file 41467_2020_17081_MOESM10_ESM.zip › File2/Confidence_VeryHigh_Taxonomy/241531_out.krona.html]

Javascript must be enabled to view this page.

members
magnitude
magnitudeUnassigned
count
unassigned
taxon
rank

241531\_out

15

2
superkingdom
15

phylum
15
1239

15
class
186801

order
15
186802

family
1
541000

1263
genus
1

1263106
species

SRS063518\_contig\_number\_38267
1

186803
family
14

14
genus
572511

species

SRS012273\_contig\_number\_34161SRS012902\_contig\_number\_contig-100\_804.804SRS016267\_contig\_number\_11850SRS042284\_contig\_number\_6556SRS057717\_contig\_number\_7797SRS075398\_contig\_number\_1077SRS077502\_contig\_number\_16279SRS097889\_contig\_number\_26809SRS1041095\_contig\_number\_78SRS1055043\_contig\_number\_26099SRS140645\_contig\_number\_1551SRS143780\_contig\_number\_22264SRS148511\_contig\_number\_21359SRS893259\_contig\_number\_2114
14
33038
