# Supplementary material for: MetaRibo-Seq measures translation in microbiomes
Source: Nat Commun. 2020 Jun 29;11:3268. doi: 10.1038/s41467-020-17081-z (PMC7324362; doi:10.1038/s41467-020-17081-z)
Supplement: Supplementary file 10 — Supplementary Data 7 [file 41467_2020_17081_MOESM10_ESM.zip › File2/Confidence_VeryHigh_Taxonomy/133321_out.krona.html]

Javascript must be enabled to view this page.

members
magnitude
magnitudeUnassigned
count
unassigned
taxon
rank

133321\_out

8


SRS021484\_contig\_number\_contig-100\_2883.43654
1

2759
1
superkingdom

1
4751
kingdom

subkingdom
1
451864

1
4890
phylum

subphylum
147537
1

class
1
4891

order
1
4892

1
1156497
family

genus
4919
1

53655
1

SRS014979\_contig\_number\_12753
species

superkingdom
6
2

phylum
1239
6

186801
5
class

5
186802
order

family
541000
4

946234
1
genus


SRS143417\_contig\_number\_16293
species
1
292800

3
1898205
species

SRS019445\_contig\_number\_19574SRS104975\_contig\_number\_19522SRS147346\_contig\_number\_23100

family
216572
1

1
459786
genus

876091
1

SRS013098\_contig\_number\_39746
species

class
91061
1

186826
1
order

family
33958
1

genus

SRS053214\_contig\_number\_10389
1578
1
