# Supplementary material for: MetaRibo-Seq measures translation in microbiomes
Source: Nat Commun. 2020 Jun 29;11:3268. doi: 10.1038/s41467-020-17081-z (PMC7324362; doi:10.1038/s41467-020-17081-z)
Supplement: Supplementary file 10 — Supplementary Data 7 [file 41467_2020_17081_MOESM10_ESM.zip › File2/Confidence_VeryHigh_Taxonomy/203573_out.krona.html]

Javascript must be enabled to view this page.

members
magnitude
magnitudeUnassigned
count
unassigned
taxon
rank

203573\_out

21

21
superkingdom
2

phylum
1239
20

class
526524
1

order
526525
1

family
128827
1

135858
genus
1

species

SRS018836\_contig\_number\_contig-100\_2.163083
1946309
1

class
186801
19

19
order
186802

1
186803
family


SRS015486\_contig\_number\_contig-100\_135.136
genus
572511
1

18
541000
family

genus
946234
18

18

SRS014613\_contig\_number\_contig-100\_9483.9483SRS014948\_contig\_number\_contig-100\_8621.95181SRS015431\_contig\_number\_80505SRS015486\_contig\_number\_1822SRS015578\_contig\_number\_contig-100\_19889.112814SRS015579\_contig\_number\_11075SRS015890\_contig\_number\_24353SRS017521\_contig\_number\_46932SRS017916\_contig\_number\_18331SRS018351\_contig\_number\_contig-100\_8594.71876SRS018984\_contig\_number\_25827SRS019381\_contig\_number\_18309SRS019601\_contig\_number\_contig-100\_5876.150445SRS019693\_contig\_number\_9748SRS021219\_contig\_number\_contig-100\_119.83216SRS022524\_contig\_number\_contig-100\_1112.32819SRS023526\_contig\_number\_6610SRS971276\_contig\_number\_contig-100\_29335.98704
species
1193534

1
phylum
203691

class
203692
1

1
order
136


SRS148196\_contig\_number\_34758
species
1951286
1
