# Supplementary material for: MetaRibo-Seq measures translation in microbiomes
Source: Nat Commun. 2020 Jun 29;11:3268. doi: 10.1038/s41467-020-17081-z (PMC7324362; doi:10.1038/s41467-020-17081-z)
Supplement: Supplementary file 10 — Supplementary Data 7 [file 41467_2020_17081_MOESM10_ESM.zip › File2/Confidence_VeryHigh_Taxonomy/157236_out.krona.html]

Javascript must be enabled to view this page.

members
magnitude
magnitudeUnassigned
count
unassigned
taxon
rank

157236\_out

8

2
6
superkingdom

6
phylum

SRS014235\_contig\_number\_33719SRS024435\_contig\_number\_32427SRS053356\_contig\_number\_46583
1239
3

class
3
186801

186802
3
order

186803
1
family

genus
572511
1

species
33038

SRS104400\_contig\_number\_51802
1

541000
1
family

genus
1263
1

2293177

SRS054905\_contig\_number\_20759
1
species

31979
1
family

genus
1
1485

1

SRS013687\_contig\_number\_27960
1519
species


SRS098571\_contig\_number\_contig-100\_765.238794SRS1041132\_contig\_number\_contig-100\_2027.53608
2
