# Supplementary material for: MetaRibo-Seq measures translation in microbiomes
Source: Nat Commun. 2020 Jun 29;11:3268. doi: 10.1038/s41467-020-17081-z (PMC7324362; doi:10.1038/s41467-020-17081-z)
Supplement: Supplementary file 10 — Supplementary Data 7 [file 41467_2020_17081_MOESM10_ESM.zip › File2/Confidence_VeryHigh_Taxonomy/88615_out.krona.html]

Javascript must be enabled to view this page.

members
magnitude
magnitudeUnassigned
count
unassigned
taxon
rank

88615\_out

10

superkingdom
10
2

1239
10
phylum

186801
class
10

order
10
186802

186806
family
10

1730
10
genus

species
10
142586

SRS017103\_contig\_number\_41541SRS017191\_contig\_number\_contig-100\_4113.80962SRS019161\_contig\_number\_contig-100\_9185.100328SRS050941\_contig\_number\_contig-100\_7882.58698SRS050998\_contig\_number\_15010SRS052697\_contig\_number\_12027SRS1041136\_contig\_number\_23289SRS142923\_contig\_number\_contig-100\_10551.60002SRS144362\_contig\_number\_27269SRS893383\_contig\_number\_30749
