# Supplementary material for: MetaRibo-Seq measures translation in microbiomes
Source: Nat Commun. 2020 Jun 29;11:3268. doi: 10.1038/s41467-020-17081-z (PMC7324362; doi:10.1038/s41467-020-17081-z)
Supplement: Supplementary file 10 — Supplementary Data 7 [file 41467_2020_17081_MOESM10_ESM.zip › File2/Confidence_VeryHigh_Taxonomy/30239_out.krona.html]

Javascript must be enabled to view this page.

members
magnitude
magnitudeUnassigned
count
unassigned
taxon
rank

30239\_out

26

26
superkingdom
2

1239
phylum
26

class
909932
1

1
1843488
order

1
909930
family

1
904
genus

species
1203555

SRS023715\_contig\_number\_2175
1

25
186801
class

25
order
186802

family
541000
17

17
species
1898205

SRS011134\_contig\_number\_28510SRS015431\_contig\_number\_86271SRS015663\_contig\_number\_9815SRS018541\_contig\_number\_12425SRS019068\_contig\_number\_100218SRS019161\_contig\_number\_contig-100\_1796.169756SRS020233\_contig\_number\_23068SRS020328\_contig\_number\_contig-100\_115.217741SRS045004\_contig\_number\_contig-100\_380.187157SRS045645\_contig\_number\_4431SRS049896\_contig\_number\_contig-100\_1626.184265SRS064757\_contig\_number\_18004SRS103987\_contig\_number\_12212SRS104693\_contig\_number\_contig-100\_19871.19872SRS104975\_contig\_number\_contig-100\_3384.3385SRS144603\_contig\_number\_contig-100\_9641.53287SRS893383\_contig\_number\_7770

family
186806
8

8
genus
1730

8
species
1262882

SRS013521\_contig\_number\_13757SRS013951\_contig\_number\_35836SRS045713\_contig\_number\_7098SRS053356\_contig\_number\_38168SRS075078\_contig\_number\_29972SRS144537\_contig\_number\_2095SRS147346\_contig\_number\_57125SRS148319\_contig\_number\_16906
