# Supplementary material for: MetaRibo-Seq measures translation in microbiomes
Source: Nat Commun. 2020 Jun 29;11:3268. doi: 10.1038/s41467-020-17081-z (PMC7324362; doi:10.1038/s41467-020-17081-z)
Supplement: Supplementary file 10 — Supplementary Data 7 [file 41467_2020_17081_MOESM10_ESM.zip › File2/Confidence_VeryHigh_Taxonomy/378562_out.krona.html]

Javascript must be enabled to view this page.

members
magnitude
magnitudeUnassigned
count
unassigned
taxon
rank

378562\_out

10

superkingdom
10
2

1239
phylum
10

class
10
186801

10
order
186802

family
3
541000

genus
3
1926663


SRS021153\_contig\_number\_4354SRS046712\_contig\_number\_contig-100\_118.119SRS055966\_contig\_number\_contig-100\_167.168
3
species
1841867

family
7
31979

genus
7

SRS015264\_contig\_number\_contig-100\_10668.10668
1
580596


SRS013951\_contig\_number\_contig-100\_44394.44394
1
species
2292297

1828554
species
1

SRS149879\_contig\_number\_1328

2292004

SRS893270\_contig\_number\_10195
1
species

1

SRS016989\_contig\_number\_1117
species
2292005

species
2

SRS1041033\_contig\_number\_9847SRS148721\_contig\_number\_3964
1828555
