# Supplementary material for: MetaRibo-Seq measures translation in microbiomes
Source: Nat Commun. 2020 Jun 29;11:3268. doi: 10.1038/s41467-020-17081-z (PMC7324362; doi:10.1038/s41467-020-17081-z)
Supplement: Supplementary file 10 — Supplementary Data 7 [file 41467_2020_17081_MOESM10_ESM.zip › File2/Confidence_VeryHigh_Taxonomy/203341_out.krona.html]

Javascript must be enabled to view this page.

members
magnitude
magnitudeUnassigned
count
unassigned
taxon
rank

203341\_out

10

2
superkingdom
9

1239
phylum
8

3
class
186801

186802
order
3

1
family
541000

244127
genus
1

species

SRS065176\_contig\_number\_6511
169435
1

186803
family
2

2
572511
genus

33039
species

SRS015264\_contig\_number\_contig-100\_26165.26165SRS023914\_contig\_number\_contig-100\_17460.63110
2

species

SRS015782\_contig\_number\_43494SRS019068\_contig\_number\_119211SRS049995\_contig\_number\_39078SRS142503\_contig\_number\_28213SRS143895\_contig\_number\_46325
1263006
5

1
201174
phylum

class
1760
1

order
85006
1

1
family
85023

1
33882
genus

1
species

SRS042628\_contig\_number\_21108
2014534


SRS012969\_contig\_number\_contig-100\_6553.43093
1
