# Supplementary material for: MetaRibo-Seq measures translation in microbiomes
Source: Nat Commun. 2020 Jun 29;11:3268. doi: 10.1038/s41467-020-17081-z (PMC7324362; doi:10.1038/s41467-020-17081-z)
Supplement: Supplementary file 10 — Supplementary Data 7 [file 41467_2020_17081_MOESM10_ESM.zip › File2/Confidence_VeryHigh_Taxonomy/33628_out.krona.html]

Javascript must be enabled to view this page.

members
magnitude
magnitudeUnassigned
count
unassigned
taxon
rank

33628\_out

8

2
superkingdom
8

8
phylum
1239

8
class
186801


SRS014683\_contig\_number\_contig-100\_43327.43327SRS017191\_contig\_number\_contig-100\_3889.124278SRS019030\_contig\_number\_19193SRS019030\_contig\_number\_19509SRS043701\_contig\_number\_17620SRS053398\_contig\_number\_contig-100\_37969.76844SRS078242\_contig\_number\_25844SRS103987\_contig\_number\_contig-100\_426.209030
8
species
2044939
