# Supplementary material for: MetaRibo-Seq measures translation in microbiomes
Source: Nat Commun. 2020 Jun 29;11:3268. doi: 10.1038/s41467-020-17081-z (PMC7324362; doi:10.1038/s41467-020-17081-z)
Supplement: Supplementary file 10 — Supplementary Data 7 [file 41467_2020_17081_MOESM10_ESM.zip › File2/Confidence_VeryHigh_Taxonomy/290681_out.krona.html]

Javascript must be enabled to view this page.

members
magnitude
magnitudeUnassigned
count
unassigned
taxon
rank

290681\_out

97

superkingdom
2
97

97
976
phylum

class
97
200643

97
171549
order

family
815
97

genus
816
97

species

SRS023346\_contig\_number\_528
1
818

species

SRS147377\_contig\_number\_149
1
821


SRS011084\_contig\_number\_contig-100\_984.208149SRS012902\_contig\_number\_91SRS013098\_contig\_number\_contig-100\_962.263767SRS013158\_contig\_number\_224SRS013215\_contig\_number\_823SRS014459\_contig\_number\_328SRS014613\_contig\_number\_14108SRS014736\_contig\_number\_2109SRS015190\_contig\_number\_14768SRS015190\_contig\_number\_contig-100\_1507.90780SRS015264\_contig\_number\_3755SRS015578\_contig\_number\_4159SRS015890\_contig\_number\_1573SRS015960\_contig\_number\_1294SRS016095\_contig\_number\_31SRS016132\_contig\_number\_contig-100\_509.36483SRS016267\_contig\_number\_11646SRS017247\_contig\_number\_4404SRS017433\_contig\_number\_1056SRS017521\_contig\_number\_16292SRS017622\_contig\_number\_353SRS018313\_contig\_number\_5223SRS018541\_contig\_number\_371SRS018656\_contig\_number\_8240SRS018817\_contig\_number\_11882SRS018888\_contig\_number\_6424SRS018984\_contig\_number\_47SRS019601\_contig\_number\_29SRS019787\_contig\_number\_contig-100\_1696.74039SRS020869\_contig\_number\_5987SRS023829\_contig\_number\_501SRS023971\_contig\_number\_680SRS024075\_contig\_number\_98SRS024132\_contig\_number\_28345SRS024388\_contig\_number\_3024SRS024549\_contig\_number\_41SRS043411\_contig\_number\_663SRS043411\_contig\_number\_11067SRS043701\_contig\_number\_87SRS046502\_contig\_number\_9449SRS046712\_contig\_number\_786SRS048981\_contig\_number\_506SRS049402\_contig\_number\_13860SRS050299\_contig\_number\_1028SRS051610\_contig\_number\_2383SRS053214\_contig\_number\_18953SRS053573\_contig\_number\_contig-100\_1581.36285SRS055966\_contig\_number\_870SRS056259\_contig\_number\_1965SRS056519\_contig\_number\_1532SRS057717\_contig\_number\_2248SRS058723\_contig\_number\_654SRS063040\_contig\_number\_23516SRS063190\_contig\_number\_6649SRS063985\_contig\_number\_15818SRS064276\_contig\_number\_contig-100\_702.207266SRS074964\_contig\_number\_1477SRS075021\_contig\_number\_379SRS075398\_contig\_number\_contig-100\_1200.27152SRS075773\_contig\_number\_32957SRS075878\_contig\_number\_9791SRS076804\_contig\_number\_9140SRS077194\_contig\_number\_1265SRS077335\_contig\_number\_2987SRS077730\_contig\_number\_11071SRS098655\_contig\_number\_7643SRS098717\_contig\_number\_17471SRS098881\_contig\_number\_1535SRS104036\_contig\_number\_6645SRS1041129\_contig\_number\_452SRS104311\_contig\_number\_30853SRS104485\_contig\_number\_186SRS104636\_contig\_number\_16560SRS1055022\_contig\_number\_2352SRS1055043\_contig\_number\_contig-100\_2305.147179SRS1055069\_contig\_number\_15553SRS1055099\_contig\_number\_2624SRS140492\_contig\_number\_321SRS140513\_contig\_number\_9351SRS142599\_contig\_number\_contig-100\_1118.176089SRS143342\_contig\_number\_239SRS143417\_contig\_number\_14669SRS144135\_contig\_number\_19964SRS144362\_contig\_number\_4701SRS144506\_contig\_number\_22592SRS146812\_contig\_number\_35801SRS147022\_contig\_number\_8532SRS147139\_contig\_number\_43551SRS147653\_contig\_number\_1183SRS148159\_contig\_number\_21047SRS148970\_contig\_number\_contig-100\_882.883SRS893279\_contig\_number\_258SRS893279\_contig\_number\_8200SRS893342\_contig\_number\_235
817
94
species

species
1
457392

SRS147557\_contig\_number\_32909
