# Supplementary material for: MetaRibo-Seq measures translation in microbiomes
Source: Nat Commun. 2020 Jun 29;11:3268. doi: 10.1038/s41467-020-17081-z (PMC7324362; doi:10.1038/s41467-020-17081-z)
Supplement: Supplementary file 10 — Supplementary Data 7 [file 41467_2020_17081_MOESM10_ESM.zip › File2/Confidence_VeryHigh_Taxonomy/335173_out.krona.html]

Javascript must be enabled to view this page.

members
magnitude
magnitudeUnassigned
count
unassigned
taxon
rank

335173\_out

5

2759
2
superkingdom

4751
2
kingdom

subkingdom
451864
2

5204
1

SRS051031\_contig\_number\_27411
phylum

phylum
4890
1

1
147538
subphylum

147541
1
class

order
1
451869

family
1
45131

genus
66739
1

45133
1

SRS015190\_contig\_number\_3777
species

2
2
superkingdom

phylum
201174
2

class
2
1760

order
2
85011

family
2062
2

1883

SRS023914\_contig\_number\_5634SRS098571\_contig\_number\_56233
2
genus

1

SRS013687\_contig\_number\_contig-100\_11698.102598
