# Supplementary material for: MetaRibo-Seq measures translation in microbiomes
Source: Nat Commun. 2020 Jun 29;11:3268. doi: 10.1038/s41467-020-17081-z (PMC7324362; doi:10.1038/s41467-020-17081-z)
Supplement: Supplementary file 10 — Supplementary Data 7 [file 41467_2020_17081_MOESM10_ESM.zip › File2/Confidence_VeryHigh_Taxonomy/333514_out.krona.html]

Javascript must be enabled to view this page.

members
magnitude
magnitudeUnassigned
count
unassigned
taxon
rank

333514\_out

61

61
superkingdom
2

phylum
976
61

200643
class
61

61
order
171549

59
171552
family

59
838
genus

2292365
species

SRS023526\_contig\_number\_34146SRS077641\_contig\_number\_18300
2

species
2292054
1

SRS012849\_contig\_number\_15477


SRS015794\_contig\_number\_5580
1
159272
species

10

SRS012969\_contig\_number\_contig-100\_1142.155930SRS012983\_contig\_number\_contig-100\_9681.9681SRS013638\_contig\_number\_contig-100\_2366.91557SRS015782\_contig\_number\_34805SRS049959\_contig\_number\_43392SRS077641\_contig\_number\_3538SRS077849\_contig\_number\_27090SRS144297\_contig\_number\_105SRS144506\_contig\_number\_51110SRS147614\_contig\_number\_4078
59823
species


SRS012916\_contig\_number\_23088SRS012969\_contig\_number\_71SRS012989\_contig\_number\_contig-100\_1089.83947.83947SRS013098\_contig\_number\_24680SRS014415\_contig\_number\_1774SRS015794\_contig\_number\_7519SRS016541\_contig\_number\_15857SRS017307\_contig\_number\_144SRS017375\_contig\_number\_contig-100\_25.26SRS018888\_contig\_number\_3019SRS019285\_contig\_number\_112SRS023526\_contig\_number\_71SRS023914\_contig\_number\_21001SRS043667\_contig\_number\_775SRS043841\_contig\_number\_contig-100\_3123.66578SRS045713\_contig\_number\_contig-100\_2855.110239SRS046502\_contig\_number\_12921SRS047044\_contig\_number\_4088SRS047433\_contig\_number\_15493SRS049712\_contig\_number\_6743SRS049959\_contig\_number\_34160SRS049995\_contig\_number\_32227SRS053356\_contig\_number\_46331SRS054059\_contig\_number\_contig-100\_962.49665SRS058145\_contig\_number\_1846SRS064757\_contig\_number\_6242SRS065397\_contig\_number\_11167SRS074586\_contig\_number\_5524SRS077849\_contig\_number\_11182SRS077863\_contig\_number\_contig-100\_6357.6357SRS077991\_contig\_number\_50SRS078176\_contig\_number\_3130SRS078419\_contig\_number\_13431SRS097958\_contig\_number\_7127SRS1041031\_contig\_number\_30919SRS1041116\_contig\_number\_516SRS1041134\_contig\_number\_contig-100\_1213.57379SRS1041140\_contig\_number\_18549SRS104327\_contig\_number\_6287SRS105082\_contig\_number\_2011SRS143181\_contig\_number\_58SRS144537\_contig\_number\_46665SRS144603\_contig\_number\_10984SRS144714\_contig\_number\_1499SRS148253\_contig\_number\_1957
45
165179
species

2
family
815

2
816
genus

species
47678
1

SRS012849\_contig\_number\_15489

310297
species
1

SRS012849\_contig\_number\_30239
