# Supplementary material for: MetaRibo-Seq measures translation in microbiomes
Source: Nat Commun. 2020 Jun 29;11:3268. doi: 10.1038/s41467-020-17081-z (PMC7324362; doi:10.1038/s41467-020-17081-z)
Supplement: Supplementary file 10 — Supplementary Data 7 [file 41467_2020_17081_MOESM10_ESM.zip › File2/Confidence_VeryHigh_Taxonomy/179825_out.krona.html]

Javascript must be enabled to view this page.

members
magnitude
magnitudeUnassigned
count
unassigned
taxon
rank

179825\_out

18

superkingdom
13
2

12
phylum
1239

186801
class
12

order
12
186802

family
1
541000

genus
1
946234


SRS1041138\_contig\_number\_12922
1
species
292800

family
6
31979

1485
genus
6

6
species

SRS017521\_contig\_number\_contig-100\_2274.251212SRS021153\_contig\_number\_contig-100\_16141.16141SRS049402\_contig\_number\_contig-100\_62.75946SRS103987\_contig\_number\_contig-100\_18973.101844SRS1041091\_contig\_number\_contig-100\_3527.110298SRS146764\_contig\_number\_28941
1650661

family
5
186803

658089
4
species

SRS024132\_contig\_number\_contig-100\_22892.178393SRS142503\_contig\_number\_22288SRS148874\_contig\_number\_216SRS893300\_contig\_number\_8261

genus
1
1506553

1531
species
1

SRS021153\_contig\_number\_248

1
phylum
976

class
1
117743

order
1
200644

49546
family
1

1
genus
104267

1
species

SRS015190\_contig\_number\_contig-100\_18162.18162
1906242

4

SRS013687\_contig\_number\_contig-100\_6888.160331SRS046717\_contig\_number\_contig-100\_9552.9553SRS104311\_contig\_number\_37242SRS146764\_contig\_number\_29010

superkingdom
1
2759

kingdom
1
4751

1
subkingdom
451864

4890
phylum
1

147538
1
subphylum

147550
1
class

subclass
1
222543

order
1
1028384

681950
family
1

genus
1
5455


SRS051610\_contig\_number\_contig-100\_2731.40824
1
species
129314
