# Supplementary material for: MetaRibo-Seq measures translation in microbiomes
Source: Nat Commun. 2020 Jun 29;11:3268. doi: 10.1038/s41467-020-17081-z (PMC7324362; doi:10.1038/s41467-020-17081-z)
Supplement: Supplementary file 10 — Supplementary Data 7 [file 41467_2020_17081_MOESM10_ESM.zip › File2/Confidence_VeryHigh_Taxonomy/273918_out.krona.html]

Javascript must be enabled to view this page.

members
magnitude
magnitudeUnassigned
count
unassigned
taxon
rank

273918\_out

1
5

SRS018936\_contig\_number\_5953

2
superkingdom
2

2
phylum
1239

class
2
909932

2
order
1843488

909930
2
family

33024
2
genus

2
species
33025

SRS065176\_contig\_number\_10156SRS148721\_contig\_number\_19404

10239
2
superkingdom

2
10841
family

1
686565
subfamily


SRS148159\_contig\_number\_contig-100\_883.326759
1986031
1
species

1
2202644
species

SRS017433\_contig\_number\_contig-100\_1670.96008
