# Supplementary material for: MetaRibo-Seq measures translation in microbiomes
Source: Nat Commun. 2020 Jun 29;11:3268. doi: 10.1038/s41467-020-17081-z (PMC7324362; doi:10.1038/s41467-020-17081-z)
Supplement: Supplementary file 10 — Supplementary Data 7 [file 41467_2020_17081_MOESM10_ESM.zip › File2/Confidence_VeryHigh_Taxonomy/203325_out.krona.html]

Javascript must be enabled to view this page.

members
magnitude
magnitudeUnassigned
count
unassigned
taxon
rank

203325\_out

8

8
superkingdom
2

phylum
8
1239

186801
8
class

order
8
186802

6
family
186806

1730
genus
6

species
3
142586

SRS015217\_contig\_number\_contig-100\_2565.187795SRS024132\_contig\_number\_contig-100\_12544.42289SRS049712\_contig\_number\_12315

39485

SRS024132\_contig\_number\_contig-100\_448.155949SRS063040\_contig\_number\_40628SRS143876\_contig\_number\_37789
3
species

186803
2
family

genus
2
841

1262947

SRS077086\_contig\_number\_2917
1
species

species
1

SRS143417\_contig\_number\_contig-100\_34091.77079
2292065
