# Supplementary material for: MetaRibo-Seq measures translation in microbiomes
Source: Nat Commun. 2020 Jun 29;11:3268. doi: 10.1038/s41467-020-17081-z (PMC7324362; doi:10.1038/s41467-020-17081-z)
Supplement: Supplementary file 10 — Supplementary Data 7 [file 41467_2020_17081_MOESM10_ESM.zip › File2/Confidence_VeryHigh_Taxonomy/145681_out.krona.html]

Javascript must be enabled to view this page.

members
magnitude
magnitudeUnassigned
count
unassigned
taxon
rank

145681\_out

4

superkingdom
1
2
4

SRS077502\_contig\_number\_15856

phylum
2
1239

186801
2
class

order
2
186802

family
186803
2

genus
2
572511

species
2
33038

SRS057717\_contig\_number\_contig-100\_72.53226SRS140645\_contig\_number\_contig-100\_52.25849

phylum
976
1

class
200643
1

1
171549
order

171552
1
family

genus
838
1

species
1
1703337

SRS023466\_contig\_number\_2829
