# Supplementary material for: MetaRibo-Seq measures translation in microbiomes
Source: Nat Commun. 2020 Jun 29;11:3268. doi: 10.1038/s41467-020-17081-z (PMC7324362; doi:10.1038/s41467-020-17081-z)
Supplement: Supplementary file 10 — Supplementary Data 7 [file 41467_2020_17081_MOESM10_ESM.zip › File2/Confidence_VeryHigh_Taxonomy/110387_out.krona.html]

Javascript must be enabled to view this page.

members
magnitude
magnitudeUnassigned
count
unassigned
taxon
rank

110387\_out

13


SRS052697\_contig\_number\_33737
1

2
12
1

SRS014235\_contig\_number\_31946
superkingdom

10
1239
phylum

species

SRS019445\_contig\_number\_16954SRS144506\_contig\_number\_37706SRS146813\_contig\_number\_8167
3
1263000

class
7
186801


SRS147271\_contig\_number\_3375
order
7
186802
1

1
541000
family

genus
1
1017280

1871015

SRS142503\_contig\_number\_11265
1
species

species

SRS015431\_contig\_number\_64401SRS1041038\_contig\_number\_contig-100\_68.36428
2
1898207

1
186806
family

1
1730
genus

species
39485

SRS1041140\_contig\_number\_21085
1

31979
1
family

1
1485
genus

species
1262848
1

SRS018541\_contig\_number\_4064

species
1

SRS054905\_contig\_number\_16522
1950927

phylum
1134404
1

species
2026749
1

SRS065397\_contig\_number\_contig-100\_18465.61896
