# Supplementary material for: MetaRibo-Seq measures translation in microbiomes
Source: Nat Commun. 2020 Jun 29;11:3268. doi: 10.1038/s41467-020-17081-z (PMC7324362; doi:10.1038/s41467-020-17081-z)
Supplement: Supplementary file 10 — Supplementary Data 7 [file 41467_2020_17081_MOESM10_ESM.zip › File2/Confidence_VeryHigh_Taxonomy/66631_out.krona.html]

Javascript must be enabled to view this page.

members
magnitude
magnitudeUnassigned
count
unassigned
taxon
rank

66631\_out

6


SRS075821\_contig\_number\_contig-100\_5895.56113
1

superkingdom
5
2

5
1239
phylum

5
186801
class

186802
5
order

family
31979
1

genus
1
1485

1776383
1

SRS098571\_contig\_number\_63612
species

family
541000
4

946234
3
genus

species

SRS022713\_contig\_number\_6467SRS098655\_contig\_number\_contig-100\_85.89302SRS1041090\_contig\_number\_2265
3
292800

genus
1
1263

species
165186

SRS046502\_contig\_number\_998
1
