# Supplementary material for: MetaRibo-Seq measures translation in microbiomes
Source: Nat Commun. 2020 Jun 29;11:3268. doi: 10.1038/s41467-020-17081-z (PMC7324362; doi:10.1038/s41467-020-17081-z)
Supplement: Supplementary file 10 — Supplementary Data 7 [file 41467_2020_17081_MOESM10_ESM.zip › File2/Confidence_VeryHigh_Taxonomy/65753_out.krona.html]

Javascript must be enabled to view this page.

members
magnitude
magnitudeUnassigned
count
unassigned
taxon
rank

65753\_out

6

superkingdom
2
6

phylum
976
6

200643
class
6

6
order
171549

family
171552
6

6
genus
838


SRS077641\_contig\_number\_2006
2292365
species
1

1
2293125
species

SRS049959\_contig\_number\_16868


SRS019910\_contig\_number\_contig-100\_455.125942SRS104912\_contig\_number\_19526SRS144537\_contig\_number\_21032
3
species
159272

1
species
2079531

SRS019808\_contig\_number\_2064
