# Supplementary material for: MetaRibo-Seq measures translation in microbiomes
Source: Nat Commun. 2020 Jun 29;11:3268. doi: 10.1038/s41467-020-17081-z (PMC7324362; doi:10.1038/s41467-020-17081-z)
Supplement: Supplementary file 10 — Supplementary Data 7 [file 41467_2020_17081_MOESM10_ESM.zip › File2/Confidence_VeryHigh_Taxonomy/117607_out.krona.html]

Javascript must be enabled to view this page.

members
magnitude
magnitudeUnassigned
count
unassigned
taxon
rank

117607\_out

4

superkingdom
2
4

1224
4
phylum

3
28216
class

order
206351
3

481
3
family

3
482
genus


SRS051930\_contig\_number\_contig-100\_423.197977SRS075872\_contig\_number\_7613SRS075876\_contig\_number\_11004
species
3
1581104

class
1236
1

order
135615
1

868
1
family

genus
2717
1

species
2718
1

SRS042984\_contig\_number\_41974
