# Supplementary material for: MetaRibo-Seq measures translation in microbiomes
Source: Nat Commun. 2020 Jun 29;11:3268. doi: 10.1038/s41467-020-17081-z (PMC7324362; doi:10.1038/s41467-020-17081-z)
Supplement: Supplementary file 10 — Supplementary Data 7 [file 41467_2020_17081_MOESM10_ESM.zip › File2/Confidence_VeryHigh_Taxonomy/180592_out.krona.html]

Javascript must be enabled to view this page.

members
magnitude
magnitudeUnassigned
count
unassigned
taxon
rank

180592\_out

7

superkingdom
2
7

7
phylum
1239

class
186801
7

1
7
186802

SRS014459\_contig\_number\_17574
order

216572
family
1

459786
genus
1


SRS147377\_contig\_number\_1773
1
species
876091


SRS050998\_contig\_number\_999
1
species
1898207

1
31979
family

genus
1485
1


SRS1054691\_contig\_number\_contig-100\_9656.47689
1
species
1776383

186806
family
1

1
1730
genus


SRS142923\_contig\_number\_23094
1
species
142586

1
family
541000

1
216851
genus


SRS014855\_contig\_number\_15074
1
species
1897006

1852363
species
1

SRS104311\_contig\_number\_24586
