# Supplementary material for: MetaRibo-Seq measures translation in microbiomes
Source: Nat Commun. 2020 Jun 29;11:3268. doi: 10.1038/s41467-020-17081-z (PMC7324362; doi:10.1038/s41467-020-17081-z)
Supplement: Supplementary file 10 — Supplementary Data 7 [file 41467_2020_17081_MOESM10_ESM.zip › File2/Confidence_VeryHigh_Taxonomy/357465_out.krona.html]

Javascript must be enabled to view this page.

members
magnitude
magnitudeUnassigned
count
unassigned
taxon
rank

357465\_out

8

2
superkingdom
8

phylum
8
976

200643
8
class

8
order
171549

171552
family
8

8
genus
838

species
2
1263102

SRS049959\_contig\_number\_38381SRS144537\_contig\_number\_46037

1262932
species
2

SRS049959\_contig\_number\_38282SRS144537\_contig\_number\_46559

3
species
165179

SRS012849\_contig\_number\_15142SRS019808\_contig\_number\_18217SRS104912\_contig\_number\_21010


SRS019910\_contig\_number\_12934
2293125
species
1
