# Supplementary material for: MetaRibo-Seq measures translation in microbiomes
Source: Nat Commun. 2020 Jun 29;11:3268. doi: 10.1038/s41467-020-17081-z (PMC7324362; doi:10.1038/s41467-020-17081-z)
Supplement: Supplementary file 10 — Supplementary Data 7 [file 41467_2020_17081_MOESM10_ESM.zip › File2/Confidence_VeryHigh_Taxonomy/87358_out.krona.html]

Javascript must be enabled to view this page.

members
magnitude
magnitudeUnassigned
count
unassigned
taxon
rank

87358\_out

19

19
superkingdom
2

phylum
1239
19

class
186801
19

19
order
186802

19
541000
family

216851
genus
19


SRS015663\_contig\_number\_14433SRS017307\_contig\_number\_contig-100\_1200.48960SRS018351\_contig\_number\_1046SRS018836\_contig\_number\_382SRS047433\_contig\_number\_12947SRS051031\_contig\_number\_contig-100\_7428.123689SRS075078\_contig\_number\_8034SRS075773\_contig\_number\_19455SRS097958\_contig\_number\_contig-100\_18703.56859SRS1041092\_contig\_number\_contig-100\_1080.63631SRS1041145\_contig\_number\_1775SRS143895\_contig\_number\_28722SRS144135\_contig\_number\_17728SRS144297\_contig\_number\_3526SRS144714\_contig\_number\_430SRS144753\_contig\_number\_5384SRS146812\_contig\_number\_12375SRS148196\_contig\_number\_1139SRS893366\_contig\_number\_20492
19
species
853
