# Supplementary material for: MetaRibo-Seq measures translation in microbiomes
Source: Nat Commun. 2020 Jun 29;11:3268. doi: 10.1038/s41467-020-17081-z (PMC7324362; doi:10.1038/s41467-020-17081-z)
Supplement: Supplementary file 10 — Supplementary Data 7 [file 41467_2020_17081_MOESM10_ESM.zip › File2/Confidence_VeryHigh_Taxonomy/91704_out.krona.html]

Javascript must be enabled to view this page.

members
magnitude
magnitudeUnassigned
count
unassigned
taxon
rank

91704\_out

6

superkingdom
6
2

1239
5
phylum

class
186801
5

186802
5
order

1897042
1
species

SRS143780\_contig\_number\_8311

4
186803
family

genus
841
4

2293136
2
species

SRS098571\_contig\_number\_64938SRS104311\_contig\_number\_34200

species

SRS022071\_contig\_number\_contig-100\_8281.159573SRS1055069\_contig\_number\_contig-100\_1625.78058
360807
2

95818
1
phylum

species

SRS098571\_contig\_number\_39929
1
1970479
