# Supplementary material for: MetaRibo-Seq measures translation in microbiomes
Source: Nat Commun. 2020 Jun 29;11:3268. doi: 10.1038/s41467-020-17081-z (PMC7324362; doi:10.1038/s41467-020-17081-z)
Supplement: Supplementary file 10 — Supplementary Data 7 [file 41467_2020_17081_MOESM10_ESM.zip › File2/Confidence_VeryHigh_Taxonomy/360644_out.krona.html]

Javascript must be enabled to view this page.

members
magnitude
magnitudeUnassigned
count
unassigned
taxon
rank

360644\_out

26

2
superkingdom
24

phylum
1
976

200643
1
class

171549
1
order

family
1
171550

2049048

SRS019030\_contig\_number\_18211
1
species

23
phylum
1239

class
23
186801

186802
23
order

1897045

SRS1041157\_contig\_number\_1246
1
species

family
7
31979

genus
1
580596

2292294

SRS144183\_contig\_number\_5679
1
species

6
genus
1485

species
6

SRS043411\_contig\_number\_14991SRS049959\_contig\_number\_43923SRS1041136\_contig\_number\_13974SRS142599\_contig\_number\_27888SRS147766\_contig\_number\_contig-100\_44.141448SRS148721\_contig\_number\_contig-100\_2506.310433
1262769

541000

SRS1041147\_contig\_number\_1414SRS1055099\_contig\_number\_4178SRS147022\_contig\_number\_1676SRS148424\_contig\_number\_14720
4
15
family

2292180

SRS062654\_contig\_number\_27793
species
1


SRS019787\_contig\_number\_contig-100\_1376.66711SRS104485\_contig\_number\_3373SRS143085\_contig\_number\_contig-100\_3510.3511SRS147377\_contig\_number\_4068SRS148511\_contig\_number\_20623SRS149244\_contig\_number\_contig-100\_13.81887
552398
6
species

3
genus
946234

1
species

SRS100021\_contig\_number\_4959
1193534

2
species
292800

SRS098655\_contig\_number\_13364SRS098717\_contig\_number\_20884


SRS144537\_contig\_number\_34201
2292270
species
1


SRS019496\_contig\_number\_contig-100\_22718.22719SRS077589\_contig\_number\_contig-100\_10843.10844
2
