# Supplementary material for: MetaRibo-Seq measures translation in microbiomes
Source: Nat Commun. 2020 Jun 29;11:3268. doi: 10.1038/s41467-020-17081-z (PMC7324362; doi:10.1038/s41467-020-17081-z)
Supplement: Supplementary file 10 — Supplementary Data 7 [file 41467_2020_17081_MOESM10_ESM.zip › File2/Confidence_VeryHigh_Taxonomy/118241_out.krona.html]

Javascript must be enabled to view this page.

members
magnitude
magnitudeUnassigned
count
unassigned
taxon
rank

118241\_out

28

superkingdom
2
28

1239
28
phylum

1
1263007

SRS049959\_contig\_number\_1796
species

class
186801
27

order
27
186802

216572
26
family

genus
26
459786

species

SRS045826\_contig\_number\_contig-100\_7337.7338SRS048164\_contig\_number\_contig-100\_6437.46251SRS050925\_contig\_number\_contig-100\_5801.170192SRS052697\_contig\_number\_contig-100\_1882.247776SRS053356\_contig\_number\_33014SRS053649\_contig\_number\_7383SRS075821\_contig\_number\_18597SRS077024\_contig\_number\_contig-100\_7224.76428SRS077392\_contig\_number\_23922SRS098571\_contig\_number\_3740SRS101376\_contig\_number\_28264SRS104084\_contig\_number\_10533SRS1041140\_contig\_number\_contig-100\_17523.53410SRS104912\_contig\_number\_contig-100\_19001.66677SRS105082\_contig\_number\_18227SRS142890\_contig\_number\_21551SRS142980\_contig\_number\_5451SRS143372\_contig\_number\_7486SRS143876\_contig\_number\_22206SRS147271\_contig\_number\_40210SRS147919\_contig\_number\_8454SRS148159\_contig\_number\_23474SRS149075\_contig\_number\_18607SRS893288\_contig\_number\_contig-100\_11265.11266SRS893378\_contig\_number\_18945SRS971275\_contig\_number\_6153
26
1897011

1898207
1

SRS142503\_contig\_number\_18540
species
