# Supplementary material for: MetaRibo-Seq measures translation in microbiomes
Source: Nat Commun. 2020 Jun 29;11:3268. doi: 10.1038/s41467-020-17081-z (PMC7324362; doi:10.1038/s41467-020-17081-z)
Supplement: Supplementary file 10 — Supplementary Data 7 [file 41467_2020_17081_MOESM10_ESM.zip › File2/Confidence_VeryHigh_Taxonomy/30851_out.krona.html]

Javascript must be enabled to view this page.

members
magnitude
magnitudeUnassigned
count
unassigned
taxon
rank

30851\_out

105

8

SRS013965\_contig\_number\_24862SRS016267\_contig\_number\_contig-100\_4096.4096SRS017103\_contig\_number\_contig-100\_9612.53695SRS020328\_contig\_number\_contig-100\_14414.44181SRS042966\_contig\_number\_1473SRS063985\_contig\_number\_contig-100\_6826.148132SRS074670\_contig\_number\_contig-100\_15025.49404SRS149181\_contig\_number\_contig-100\_4386.189665
2
105
superkingdom

phylum
3
976

3
200643
class

3
171549
order

family
815
3

genus
3
816

species
3
384639

SRS016753\_contig\_number\_contig-100\_2707.2707SRS018427\_contig\_number\_20292SRS146764\_contig\_number\_21821

94
1239
phylum

class
186801
94

order

SRS011405\_contig\_number\_7418SRS013476\_contig\_number\_contig-100\_1453.217661SRS013521\_contig\_number\_6639SRS015190\_contig\_number\_contig-100\_2096.103029SRS023715\_contig\_number\_10406SRS048870\_contig\_number\_9019SRS063518\_contig\_number\_contig-100\_3029.220760SRS074964\_contig\_number\_contig-100\_3402.38702SRS077335\_contig\_number\_contig-100\_1050.60755SRS078176\_contig\_number\_17786SRS144362\_contig\_number\_10472SRS147377\_contig\_number\_contig-100\_10654.10655SRS147919\_contig\_number\_6406SRS148253\_contig\_number\_10499SRS148319\_contig\_number\_11492SRS148784\_contig\_number\_23439
16
94
186802

family
2
31979

genus
1
1649459


SRS145308\_contig\_number\_contig-100\_3225.3226
1
1946602
species

1485
1
genus


SRS051031\_contig\_number\_36833
1
1262828
species

family
186803
12

genus
1

SRS043411\_contig\_number\_contig-100\_3384.49648
841
3

166486
1

SRS097920\_contig\_number\_23744
species

2049040
1

SRS015663\_contig\_number\_2809
species

6
28050
genus


SRS049959\_contig\_number\_39159SRS050925\_contig\_number\_contig-100\_4517.85008SRS143342\_contig\_number\_21964SRS144537\_contig\_number\_45260SRS146813\_contig\_number\_26333SRS148091\_contig\_number\_12095
28052
6
species

1
1506553
genus

species
1
1946684

SRS014855\_contig\_number\_4334


SRS015782\_contig\_number\_contig-100\_1157.1158
33042
1
genus

species

SRS019161\_contig\_number\_19432
39491
1

family
541000
1

genus

SRS144714\_contig\_number\_contig-100\_9844.45800
1263
1

63
186806
family


SRS075878\_contig\_number\_5276SRS142890\_contig\_number\_45280
2
63
1730
genus


SRS015579\_contig\_number\_11554SRS018984\_contig\_number\_26743SRS024331\_contig\_number\_36988SRS043667\_contig\_number\_4604SRS050941\_contig\_number\_contig-100\_7080.57896SRS063040\_contig\_number\_33107SRS097889\_contig\_number\_33654SRS144183\_contig\_number\_5547SRS148159\_contig\_number\_contig-100\_1652.265881
9
1262892
species


SRS015578\_contig\_number\_30402SRS052027\_contig\_number\_14501
1262889
2
species

32
142586

SRS013951\_contig\_number\_36549SRS014235\_contig\_number\_45210SRS015065\_contig\_number\_contig-100\_537.185300SRS015264\_contig\_number\_15757SRS015431\_contig\_number\_16317SRS015431\_contig\_number\_50110SRS017307\_contig\_number\_contig-100\_3863.120687SRS018313\_contig\_number\_7696SRS019068\_contig\_number\_19524SRS019267\_contig\_number\_9052SRS019787\_contig\_number\_12089SRS024663\_contig\_number\_13556SRS044535\_contig\_number\_4905SRS047044\_contig\_number\_17724SRS049896\_contig\_number\_contig-100\_1062.194625SRS049900\_contig\_number\_980SRS050422\_contig\_number\_contig-100\_374.167928SRS052027\_contig\_number\_contig-100\_17916.52717SRS056519\_contig\_number\_6866SRS058723\_contig\_number\_6128SRS065504\_contig\_number\_36983SRS077127\_contig\_number\_8438SRS077502\_contig\_number\_8439SRS098827\_contig\_number\_12363SRS100021\_contig\_number\_10529SRS100021\_contig\_number\_11766SRS1041038\_contig\_number\_8112SRS142503\_contig\_number\_49866SRS143070\_contig\_number\_8721SRS143722\_contig\_number\_contig-100\_3496.36933SRS143780\_contig\_number\_17619SRS148721\_contig\_number\_36574
species

39485
1

SRS053398\_contig\_number\_contig-100\_4707.141887
species

species

SRS014459\_contig\_number\_contig-100\_877.176855SRS016095\_contig\_number\_contig-100\_5989.138131SRS017247\_contig\_number\_contig-100\_15075.48926SRS046717\_contig\_number\_contig-100\_31113.31114SRS052697\_contig\_number\_contig-100\_573.42850SRS054956\_contig\_number\_15644SRS077849\_contig\_number\_contig-100\_34866.71056SRS1041136\_contig\_number\_contig-100\_6430.82274SRS143372\_contig\_number\_contig-100\_19431.58397SRS149075\_contig\_number\_12238
1262885
10


SRS063370\_contig\_number\_1611SRS104311\_contig\_number\_10435
1262887
2
species


SRS017191\_contig\_number\_17747SRS023346\_contig\_number\_6864SRS043701\_contig\_number\_contig-100\_76.117648SRS144135\_contig\_number\_34685SRS147271\_contig\_number\_25505
1897026
5
species
