# Supplementary material for: MetaRibo-Seq measures translation in microbiomes
Source: Nat Commun. 2020 Jun 29;11:3268. doi: 10.1038/s41467-020-17081-z (PMC7324362; doi:10.1038/s41467-020-17081-z)
Supplement: Supplementary file 10 — Supplementary Data 7 [file 41467_2020_17081_MOESM10_ESM.zip › File2/Confidence_VeryHigh_Taxonomy/134827_out.krona.html]

Javascript must be enabled to view this page.

members
magnitude
magnitudeUnassigned
count
unassigned
taxon
rank

134827\_out

16

superkingdom
15
2

phylum
2
1239

class
1
91061

1
order
186826

1300
family
1

genus
1
1301

28037
species
1

SRS019172\_contig\_number\_contig-100\_2506.2507

186801
1
class

1
order
186802

SRS016133\_contig\_number\_7818

13
phylum
32066

203490
13
class

203491
13
order

1
family
203492

1
genus
848


SRS015057\_contig\_number\_contig-100\_19.88929
860
species
1

12
family
1129771

12
genus
32067

7
species
157692

SRS014470\_contig\_number\_3876SRS050244\_contig\_number\_14045SRS143037\_contig\_number\_28159SRS147541\_contig\_number\_4767SRS148973\_contig\_number\_12918SRS149720\_contig\_number\_5064SRS893352\_contig\_number\_12844

species
2
109328

SRS042589\_contig\_number\_13427SRS144300\_contig\_number\_23846


SRS015762\_contig\_number\_contig-100\_3078.246577.246577
671213
1
species

1
species
712359

SRS144560\_contig\_number\_24007


SRS020571\_contig\_number\_27719
157687
1
species


SRS147226\_contig\_number\_contig-100\_8322.156272
1
