# Supplementary material for: MetaRibo-Seq measures translation in microbiomes
Source: Nat Commun. 2020 Jun 29;11:3268. doi: 10.1038/s41467-020-17081-z (PMC7324362; doi:10.1038/s41467-020-17081-z)
Supplement: Supplementary file 10 — Supplementary Data 7 [file 41467_2020_17081_MOESM10_ESM.zip › File2/Confidence_VeryHigh_Taxonomy/31009_out.krona.html]

Javascript must be enabled to view this page.

members
magnitude
magnitudeUnassigned
count
unassigned
taxon
rank

31009\_out

13

2
superkingdom
13

1239
phylum
13

class
186801
13

13
order
186802

13
186803
family
2

SRS012273\_contig\_number\_contig-100\_2426.246339SRS023715\_contig\_number\_contig-100\_26427.116161

7
1432051
genus

species

SRS146888\_contig\_number\_12289
1432052
1

species

SRS043768\_contig\_number\_contig-100\_39512.90514SRS105153\_contig\_number\_contig-100\_11634.11634SRS1054691\_contig\_number\_contig-100\_1292.72683SRS143780\_contig\_number\_contig-100\_8781.152223SRS146888\_contig\_number\_13502SRS148511\_contig\_number\_7647
1720294
6

genus
1407607
1

1
1806509
species

SRS148511\_contig\_number\_17097

1
658086
species

SRS143991\_contig\_number\_11031

1
39491

SRS098717\_contig\_number\_contig-100\_38.138662
species

189330
genus

SRS105153\_contig\_number\_28056
1
