# Supplementary material for: MetaRibo-Seq measures translation in microbiomes
Source: Nat Commun. 2020 Jun 29;11:3268. doi: 10.1038/s41467-020-17081-z (PMC7324362; doi:10.1038/s41467-020-17081-z)
Supplement: Supplementary file 10 — Supplementary Data 7 [file 41467_2020_17081_MOESM10_ESM.zip › File2/Confidence_VeryHigh_Taxonomy/85467_out.krona.html]

Javascript must be enabled to view this page.

members
magnitude
magnitudeUnassigned
count
unassigned
taxon
rank

85467\_out

64

2
superkingdom
64

1239
64
phylum

186801
class
64

64
order
186802

31979
family
1

1
genus
1485

1
species
2293011

SRS143417\_contig\_number\_17088

186803
family
60

28050
genus
1

1
species
28052

SRS144714\_contig\_number\_3151

2
genus
841

1
species

SRS048870\_contig\_number\_19699
2293144


SRS893342\_contig\_number\_8858
360807
1
species

39491

SRS014235\_contig\_number\_35488SRS014313\_contig\_number\_4215SRS014459\_contig\_number\_contig-100\_45975.45976SRS014736\_contig\_number\_9486SRS014855\_contig\_number\_16689SRS015190\_contig\_number\_12804SRS015217\_contig\_number\_19281SRS015486\_contig\_number\_contig-100\_1366.1367SRS015578\_contig\_number\_31382SRS015782\_contig\_number\_46922SRS016018\_contig\_number\_11796SRS016495\_contig\_number\_contig-100\_53.125303SRS017433\_contig\_number\_contig-100\_2876.116284SRS018427\_contig\_number\_28328SRS019685\_contig\_number\_21352SRS022137\_contig\_number\_22463SRS022713\_contig\_number\_contig-100\_13087.13087SRS042628\_contig\_number\_37997SRS043001\_contig\_number\_contig-100\_5397.74074SRS043411\_contig\_number\_contig-100\_880.109792SRS045004\_contig\_number\_contig-100\_5104.99122SRS047044\_contig\_number\_12966SRS047433\_contig\_number\_14426SRS048060\_contig\_number\_13769SRS048164\_contig\_number\_contig-100\_609.182079SRS049712\_contig\_number\_contig-100\_273.146097SRS050925\_contig\_number\_36268SRS051882\_contig\_number\_14583SRS053214\_contig\_number\_14351SRS053398\_contig\_number\_20136SRS054956\_contig\_number\_2701SRS055982\_contig\_number\_19868SRS057478\_contig\_number\_9045SRS058723\_contig\_number\_contig-100\_509.63744SRS075021\_contig\_number\_contig-100\_2314.62911SRS077335\_contig\_number\_contig-100\_850.60555SRS077502\_contig\_number\_18803SRS098644\_contig\_number\_26753SRS103987\_contig\_number\_32939SRS1041091\_contig\_number\_contig-100\_773.125800SRS104311\_contig\_number\_39617SRS104327\_contig\_number\_contig-100\_59.54327SRS104485\_contig\_number\_5737SRS104975\_contig\_number\_9309SRS1055049\_contig\_number\_contig-100\_1448.81738SRS1055056\_contig\_number\_2346SRS143598\_contig\_number\_contig-100\_9027.112006SRS147346\_contig\_number\_contig-100\_568.308939SRS147377\_contig\_number\_contig-100\_155.85142SRS147614\_contig\_number\_contig-100\_517.68728SRS147919\_contig\_number\_16125SRS148196\_contig\_number\_contig-100\_588.255858SRS148253\_contig\_number\_14577SRS148721\_contig\_number\_53821SRS149784\_contig\_number\_9556SRS893295\_contig\_number\_contig-100\_365.82989SRS893341\_contig\_number\_12616
57
species

family
3
186806

3
genus
1730

species
1
1262885

SRS019068\_contig\_number\_104920

1
species

SRS064276\_contig\_number\_41811
29322

39485

SRS147272\_contig\_number\_217
species
1
