# Supplementary material for: MetaRibo-Seq measures translation in microbiomes
Source: Nat Commun. 2020 Jun 29;11:3268. doi: 10.1038/s41467-020-17081-z (PMC7324362; doi:10.1038/s41467-020-17081-z)
Supplement: Supplementary file 10 — Supplementary Data 7 [file 41467_2020_17081_MOESM10_ESM.zip › File2/Confidence_VeryHigh_Taxonomy/83015_out.krona.html]

Javascript must be enabled to view this page.

members
magnitude
magnitudeUnassigned
count
unassigned
taxon
rank

83015\_out

20

2
20
superkingdom

phylum
4
976

4
200643
class

order
171549
4

3
171552
family

genus
838
3


SRS011134\_contig\_number\_6650SRS022609\_contig\_number\_contig-100\_1421.246609SRS049959\_contig\_number\_1891
species
165179
3

family
815
1

816
1
genus

818
1
species

SRS144537\_contig\_number\_45268

phylum
204428
16

204429
16
class

order
16
51291

16
809
family

genus
810
16

813
16

SRS014415\_contig\_number\_1891SRS015590\_contig\_number\_contig-100\_1307.101246SRS017307\_contig\_number\_contig-100\_2125.154506SRS019285\_contig\_number\_contig-100\_343.22233SRS043841\_contig\_number\_10621SRS044535\_contig\_number\_24047SRS045195\_contig\_number\_contig-100\_1779.59843SRS046502\_contig\_number\_contig-100\_1310.68474SRS047433\_contig\_number\_contig-100\_2240.103950SRS049959\_contig\_number\_contig-100\_2208.275874SRS051610\_contig\_number\_contig-100\_1767.59594SRS054059\_contig\_number\_contig-100\_1267.36620SRS065397\_contig\_number\_contig-100\_1243.92290SRS1041134\_contig\_number\_contig-100\_4892.61058SRS105070\_contig\_number\_contig-100\_439.439SRS143729\_contig\_number\_contig-100\_1437.1437
species
