# Supplementary material for: MetaRibo-Seq measures translation in microbiomes
Source: Nat Commun. 2020 Jun 29;11:3268. doi: 10.1038/s41467-020-17081-z (PMC7324362; doi:10.1038/s41467-020-17081-z)
Supplement: Supplementary file 10 — Supplementary Data 7 [file 41467_2020_17081_MOESM10_ESM.zip › File2/Confidence_VeryHigh_Taxonomy/121918_out.krona.html]

Javascript must be enabled to view this page.

members
magnitude
magnitudeUnassigned
count
unassigned
taxon
rank

121918\_out

5

2
5
superkingdom

5
1239
phylum

class
186801
5

order
1
5

SRS077502\_contig\_number\_contig-100\_1202.114066
186802

family
4
541000

4
946234
genus

species
1193534

SRS018427\_contig\_number\_422SRS078665\_contig\_number\_contig-100\_6821.42766SRS104485\_contig\_number\_contig-100\_3784.3784SRS142599\_contig\_number\_contig-100\_4294.98078
4
