# Supplementary material for: MetaRibo-Seq measures translation in microbiomes
Source: Nat Commun. 2020 Jun 29;11:3268. doi: 10.1038/s41467-020-17081-z (PMC7324362; doi:10.1038/s41467-020-17081-z)
Supplement: Supplementary file 10 — Supplementary Data 7 [file 41467_2020_17081_MOESM10_ESM.zip › File2/Confidence_VeryHigh_Taxonomy/306500_out.krona.html]

Javascript must be enabled to view this page.

members
magnitude
magnitudeUnassigned
count
unassigned
taxon
rank

306500\_out

102

superkingdom
2
102

1239
101
phylum

class
186801
101

order
101
186802

family
216572
101

genus
31

SRS016056\_contig\_number\_contig-100\_12308.12308SRS018313\_contig\_number\_contig-100\_17617.50778SRS020233\_contig\_number\_contig-100\_34941.161037SRS020869\_contig\_number\_contig-100\_38574.123294SRS022713\_contig\_number\_5998SRS048060\_contig\_number\_8562SRS048164\_contig\_number\_contig-100\_18048.102286SRS049773\_contig\_number\_13449SRS051610\_contig\_number\_6731SRS054590\_contig\_number\_203SRS054956\_contig\_number\_15204SRS056519\_contig\_number\_10904SRS057478\_contig\_number\_7203SRS065397\_contig\_number\_contig-100\_4059.4060SRS074964\_contig\_number\_contig-100\_13062.13062SRS076804\_contig\_number\_17111SRS077086\_contig\_number\_contig-100\_35710.35710SRS077335\_contig\_number\_contig-100\_6837.6837SRS077454\_contig\_number\_2690SRS1041036\_contig\_number\_3318SRS1041090\_contig\_number\_contig-100\_6223.6224SRS1041144\_contig\_number\_5828SRS1041146\_contig\_number\_contig-100\_10680.10681SRS104327\_contig\_number\_4972SRS104485\_contig\_number\_contig-100\_1039.41897SRS1055076\_contig\_number\_6984SRS1055099\_contig\_number\_contig-100\_2431.70798SRS142923\_contig\_number\_8800SRS147139\_contig\_number\_40506SRS148319\_contig\_number\_contig-100\_5301.71362SRS148424\_contig\_number\_18055
101
459786

species
1262910

SRS971275\_contig\_number\_39350
1

species
2109687

SRS098655\_contig\_number\_1305SRS1041112\_contig\_number\_5065SRS142890\_contig\_number\_5687SRS146888\_contig\_number\_7999SRS148253\_contig\_number\_13486
5

61

SRS013098\_contig\_number\_contig-100\_2134.278202SRS015217\_contig\_number\_30521SRS016132\_contig\_number\_5299SRS017103\_contig\_number\_21725SRS017521\_contig\_number\_36309SRS017916\_contig\_number\_17380SRS018427\_contig\_number\_26593SRS019286\_contig\_number\_contig-100\_12262.12263SRS023715\_contig\_number\_9948SRS043411\_contig\_number\_14445SRS046712\_contig\_number\_2729SRS048981\_contig\_number\_4248SRS049959\_contig\_number\_3747SRS050026\_contig\_number\_1409SRS053214\_contig\_number\_16030SRS064232\_contig\_number\_2196SRS075878\_contig\_number\_19040SRS076976\_contig\_number\_2166SRS077194\_contig\_number\_contig-100\_3982.148918SRS077552\_contig\_number\_26814SRS077589\_contig\_number\_contig-100\_1119.44411SRS078242\_contig\_number\_7686SRS097889\_contig\_number\_contig-100\_1389.217514SRS097958\_contig\_number\_10650SRS098571\_contig\_number\_52619SRS098717\_contig\_number\_15792SRS100021\_contig\_number\_11577SRS103987\_contig\_number\_30807SRS104084\_contig\_number\_contig-100\_122.42550SRS1041091\_contig\_number\_423SRS1041136\_contig\_number\_12575SRS104400\_contig\_number\_46481SRS104636\_contig\_number\_16063SRS105153\_contig\_number\_10058SRS1054691\_contig\_number\_19933SRS1054716\_contig\_number\_9833SRS1055043\_contig\_number\_10027SRS1055049\_contig\_number\_2140SRS1055067\_contig\_number\_10844SRS142599\_contig\_number\_25767SRS142712\_contig\_number\_2293SRS143342\_contig\_number\_18442SRS143780\_contig\_number\_contig-100\_1906.188673SRS143876\_contig\_number\_30858SRS144297\_contig\_number\_8544SRS144362\_contig\_number\_15315SRS144537\_contig\_number\_8205SRS144603\_contig\_number\_2388SRS146764\_contig\_number\_21837SRS147022\_contig\_number\_contig-100\_983.89704SRS147377\_contig\_number\_contig-100\_237.41690SRS147766\_contig\_number\_contig-100\_1824.137950SRS147919\_contig\_number\_7228SRS148511\_contig\_number\_6387SRS148721\_contig\_number\_33856SRS148784\_contig\_number\_26579SRS148874\_contig\_number\_6721SRS149181\_contig\_number\_8738SRS893253\_contig\_number\_2265SRS893256\_contig\_number\_1582SRS893270\_contig\_number\_2765
876091
species

3

SRS104311\_contig\_number\_42475SRS143991\_contig\_number\_17989SRS147346\_contig\_number\_33892
1945593
species

phylum
1
976

class
1
200643

order
1
171549

family
815
1

1
816
genus

species

SRS097920\_contig\_number\_9246
1
28116
