# Supplementary material for: MetaRibo-Seq measures translation in microbiomes
Source: Nat Commun. 2020 Jun 29;11:3268. doi: 10.1038/s41467-020-17081-z (PMC7324362; doi:10.1038/s41467-020-17081-z)
Supplement: Supplementary file 10 — Supplementary Data 7 [file 41467_2020_17081_MOESM10_ESM.zip › File2/Confidence_VeryHigh_Taxonomy/227971_out.krona.html]

Javascript must be enabled to view this page.

members
magnitude
magnitudeUnassigned
count
unassigned
taxon
rank

227971\_out

4

2
4
superkingdom

4
976
phylum

class
4
200643

4
171549
order

family
815
4

816
4
genus


SRS018836\_contig\_number\_27900SRS144506\_contig\_number\_contig-100\_2772.234835SRS148196\_contig\_number\_contig-100\_814.238258
species
2291999
3

2292002
1

SRS020233\_contig\_number\_contig-100\_17601.216212
species
