# Supplementary material for: MetaRibo-Seq measures translation in microbiomes
Source: Nat Commun. 2020 Jun 29;11:3268. doi: 10.1038/s41467-020-17081-z (PMC7324362; doi:10.1038/s41467-020-17081-z)
Supplement: Supplementary file 10 — Supplementary Data 7 [file 41467_2020_17081_MOESM10_ESM.zip › File2/Confidence_VeryHigh_Taxonomy/98228_out.krona.html]

Javascript must be enabled to view this page.

members
magnitude
magnitudeUnassigned
count
unassigned
taxon
rank

98228\_out

20

2
20
superkingdom

20
1239
phylum

20
186801
class


SRS013965\_contig\_number\_contig-100\_25491.126493SRS014855\_contig\_number\_contig-100\_5996.5997SRS014923\_contig\_number\_contig-100\_7342.184956SRS097889\_contig\_number\_contig-100\_9260.9260SRS098644\_contig\_number\_contig-100\_3346.3346SRS143876\_contig\_number\_10383SRS893256\_contig\_number\_contig-100\_7740.7741
7
order
186802
20

family
31979
4

genus
1649459
3


SRS016495\_contig\_number\_contig-100\_2545.97153SRS019910\_contig\_number\_contig-100\_1648.118983SRS971275\_contig\_number\_9459
species
1946603
3

genus
1
1485

species

SRS147425\_contig\_number\_611
1
1262776

family
541000
8


SRS015217\_contig\_number\_3016SRS019601\_contig\_number\_29189SRS148091\_contig\_number\_12948SRS148511\_contig\_number\_19947
species
1627893
4

1

SRS056519\_contig\_number\_16357
292632
3
genus


SRS015217\_contig\_number\_27297SRS098655\_contig\_number\_14088
species
2302961
2

1
216851
genus

853
1
species

SRS098717\_contig\_number\_23746

family

SRS018427\_contig\_number\_14551
1
186803
