# Supplementary material for: MetaRibo-Seq measures translation in microbiomes
Source: Nat Commun. 2020 Jun 29;11:3268. doi: 10.1038/s41467-020-17081-z (PMC7324362; doi:10.1038/s41467-020-17081-z)
Supplement: Supplementary file 10 — Supplementary Data 7 [file 41467_2020_17081_MOESM10_ESM.zip › File2/Confidence_VeryHigh_Taxonomy/155749_out.krona.html]

Javascript must be enabled to view this page.

members
magnitude
magnitudeUnassigned
count
unassigned
taxon
rank

155749\_out

21

2
superkingdom
21

11
phylum
1239

11
class
186801

186802
order
11

9
family
31979

1485
3

SRS018157\_contig\_number\_26666SRS024087\_contig\_number\_39306SRS049995\_contig\_number\_contig-100\_225.225
9
genus

species
1

SRS013723\_contig\_number\_44487
179628

59620
species
1

SRS019028\_contig\_number\_16290

1896978

SRS064260\_contig\_number\_contig-100\_13875.97113SRS147386\_contig\_number\_12282
species
2

1262810

SRS077849\_contig\_number\_contig-100\_4004.170003SRS144506\_contig\_number\_54517
2
species

186804
1
family

1870884
genus
1

1
species

SRS103983\_contig\_number\_18017
1496

species
1

SRS140492\_contig\_number\_contig-100\_44403.80727
1950927

544448
1
phylum


SRS015893\_contig\_number\_4687
species
1
1911684

32066
phylum
8

8
class
203490

203491
8
order

203492
family
8

genus
8
848

2
species

SRS013252\_contig\_number\_3186
1
851

76857

SRS017227\_contig\_number\_contig-100\_60.60
subspecies
1

5
species

SRS011152\_contig\_number\_23353SRS012285\_contig\_number\_contig-100\_4752.174406SRS018826\_contig\_number\_contig-100\_8110.212640SRS019077\_contig\_number\_24545SRS064449\_contig\_number\_38828
860


SRS1055086\_contig\_number\_2026
1
species
1583098

phylum
1
1224

class
1
28216

1
order
206351

family
1
481

482
1
genus

487
1
species

SRS147126\_contig\_number\_13224
