# Supplementary material for: MetaRibo-Seq measures translation in microbiomes
Source: Nat Commun. 2020 Jun 29;11:3268. doi: 10.1038/s41467-020-17081-z (PMC7324362; doi:10.1038/s41467-020-17081-z)
Supplement: Supplementary file 10 — Supplementary Data 7 [file 41467_2020_17081_MOESM10_ESM.zip › File2/Confidence_VeryHigh_Taxonomy/83_out.krona.html]

Javascript must be enabled to view this page.

members
magnitude
magnitudeUnassigned
count
unassigned
taxon
rank

83\_out

19

19
superkingdom
2

1224
1
phylum

class
1
28211

order
1
204441

41295
1
family

1
genus
191


SRS022071\_contig\_number\_31938
species
1
34012

18
phylum
1239

4
species

SRS062654\_contig\_number\_contig-100\_31112.73877SRS076929\_contig\_number\_3631SRS1055038\_contig\_number\_contig-100\_7023.7024SRS147271\_contig\_number\_contig-100\_27468.112521
1262988

class
14
186801

order
14
186802

family
13
186803


SRS011084\_contig\_number\_29697SRS011134\_contig\_number\_48095SRS022609\_contig\_number\_contig-100\_9814.144716SRS023914\_contig\_number\_16012SRS024132\_contig\_number\_12033SRS049959\_contig\_number\_51301SRS055017\_contig\_number\_16137SRS056259\_contig\_number\_contig-100\_7318.125701SRS065504\_contig\_number\_21819SRS075773\_contig\_number\_19857SRS1041133\_contig\_number\_10990SRS144537\_contig\_number\_40337SRS893383\_contig\_number\_32979
species
13
1898203

species

SRS049773\_contig\_number\_24225
1
1898207
