# Supplementary material for: MetaRibo-Seq measures translation in microbiomes
Source: Nat Commun. 2020 Jun 29;11:3268. doi: 10.1038/s41467-020-17081-z (PMC7324362; doi:10.1038/s41467-020-17081-z)
Supplement: Supplementary file 10 — Supplementary Data 7 [file 41467_2020_17081_MOESM10_ESM.zip › File2/Confidence_VeryHigh_Taxonomy/267407_out.krona.html]

Javascript must be enabled to view this page.

members
magnitude
magnitudeUnassigned
count
unassigned
taxon
rank

267407\_out

13

2
13
superkingdom

phylum
1
203691

1
class
203692

136
order
1

species
1

SRS148196\_contig\_number\_34758
1951286

1239
phylum
12

class
8
186801

order
8
186802

7
family
541000

216851
7
genus

5
species
1971605

SRS045826\_contig\_number\_8365SRS075716\_contig\_number\_contig-100\_6318.6319SRS075821\_contig\_number\_22189SRS098571\_contig\_number\_58602SRS142781\_contig\_number\_3928

2
species
2292356

SRS052078\_contig\_number\_3358SRS143523\_contig\_number\_contig-100\_2062.108839

186803
1
family

1
species
742723

SRS893256\_contig\_number\_2963

species
3

SRS012849\_contig\_number\_17572SRS1041129\_contig\_number\_11082SRS144537\_contig\_number\_23176
1263006

1
class
526524

1
order
526525

128827
family
1

135858
1
genus

1946309

SRS018836\_contig\_number\_contig-100\_2.163083
species
1
