# Supplementary material for: MetaRibo-Seq measures translation in microbiomes
Source: Nat Commun. 2020 Jun 29;11:3268. doi: 10.1038/s41467-020-17081-z (PMC7324362; doi:10.1038/s41467-020-17081-z)
Supplement: Supplementary file 10 — Supplementary Data 7 [file 41467_2020_17081_MOESM10_ESM.zip › File2/Confidence_VeryHigh_Taxonomy/181950_out.krona.html]

Javascript must be enabled to view this page.

members
magnitude
magnitudeUnassigned
count
unassigned
taxon
rank

181950\_out

16

1

SRS019601\_contig\_number\_contig-100\_8021.48377

2759
superkingdom
1

33208
1
kingdom

1
phylum
7711

subphylum
1
89593

1
superclass
8287

1
class
40674

314146
1
superorder

9443
1
order

376913
suborder
1

314293
1
infraorder

parvorder
1
9526

1
superfamily
314295

9604
1
family

207598
subfamily
1

9605
1
genus

9606

SRS043411\_contig\_number\_contig-100\_7392.53656
species
1

1
superkingdom
14
2

SRS097889\_contig\_number\_contig-100\_1840.207695

phylum
6
976

6
class
200643

order
6
171549


SRS053214\_contig\_number\_17728
1950409
species
1

family
1
171552

species
1
370804

SRS143598\_contig\_number\_23620

4
family
1853231

4
genus
283168

626933

SRS013098\_contig\_number\_contig-100\_366.263171SRS019068\_contig\_number\_8149SRS056519\_contig\_number\_contig-100\_124.105772SRS104636\_contig\_number\_contig-100\_188.132548
species
4

4
phylum
1224

subphylum
4
68525

class
4
28221

order
4
213118

213119
4
family

4
genus
2289

SRS014683\_contig\_number\_7348SRS015794\_contig\_number\_contig-100\_2430.2430SRS019445\_contig\_number\_5665SRS064276\_contig\_number\_contig-100\_6181.101859

1239
3
phylum

class
3
186801

186802
3
order


SRS014923\_contig\_number\_contig-100\_2482.91256SRS015578\_contig\_number\_6731SRS104311\_contig\_number\_37225
1676156
species
3
