# Supplementary material for: MetaRibo-Seq measures translation in microbiomes
Source: Nat Commun. 2020 Jun 29;11:3268. doi: 10.1038/s41467-020-17081-z (PMC7324362; doi:10.1038/s41467-020-17081-z)
Supplement: Supplementary file 10 — Supplementary Data 7 [file 41467_2020_17081_MOESM10_ESM.zip › File2/Confidence_VeryHigh_Taxonomy/57009_out.krona.html]

Javascript must be enabled to view this page.

members
magnitude
magnitudeUnassigned
count
unassigned
taxon
rank

57009\_out

1

SRS043768\_contig\_number\_24330
21

1

SRS014287\_contig\_number\_contig-100\_11352.87960

2
19
superkingdom

1239
phylum
19

186801
class
19

order
1
19
186802

SRS147139\_contig\_number\_8679

186803
family
2


SRS149879\_contig\_number\_1499
1898203
species
1


SRS1041038\_contig\_number\_contig-100\_763.74320
2305244
1
species

family
16
31979

1485
16
genus

11
species
1262824

SRS011084\_contig\_number\_4920SRS014459\_contig\_number\_34149SRS015065\_contig\_number\_17158SRS021484\_contig\_number\_12940SRS024549\_contig\_number\_contig-100\_5386.5386SRS098644\_contig\_number\_contig-100\_20613.137168SRS104084\_contig\_number\_4832SRS1041095\_contig\_number\_2714SRS1055069\_contig\_number\_4141SRS143991\_contig\_number\_contig-100\_22340.22340SRS147766\_contig\_number\_contig-100\_1832.24876

species
2
1262842

SRS015890\_contig\_number\_11325SRS019397\_contig\_number\_contig-100\_1984.1984

species
1
2292308

SRS142503\_contig\_number\_contig-100\_2458.94299

1
species
2292008

SRS076929\_contig\_number\_6375


SRS100021\_contig\_number\_11293
59620
1
species
