# Supplementary material for: MetaRibo-Seq measures translation in microbiomes
Source: Nat Commun. 2020 Jun 29;11:3268. doi: 10.1038/s41467-020-17081-z (PMC7324362; doi:10.1038/s41467-020-17081-z)
Supplement: Supplementary file 10 — Supplementary Data 7 [file 41467_2020_17081_MOESM10_ESM.zip › File2/Confidence_VeryHigh_Taxonomy/200248_out.krona.html]

Javascript must be enabled to view this page.

members
magnitude
magnitudeUnassigned
count
unassigned
taxon
rank

200248\_out

11

superkingdom
11
2

phylum
11
976

class
11
200643

171549
11
order

2005525
11
family

11
2
genus

SRS011239\_contig\_number\_14662SRS971275\_contig\_number\_contig-100\_2106.209367
375288

2292242

SRS020869\_contig\_number\_25016
species
1

8
species

SRS020328\_contig\_number\_21277SRS023715\_contig\_number\_12097SRS049959\_contig\_number\_5689SRS055966\_contig\_number\_1956SRS056259\_contig\_number\_9393SRS063127\_contig\_number\_3215SRS1055043\_contig\_number\_2881SRS1055099\_contig\_number\_5164
328812
