# Supplementary material for: MetaRibo-Seq measures translation in microbiomes
Source: Nat Commun. 2020 Jun 29;11:3268. doi: 10.1038/s41467-020-17081-z (PMC7324362; doi:10.1038/s41467-020-17081-z)
Supplement: Supplementary file 10 — Supplementary Data 7 [file 41467_2020_17081_MOESM10_ESM.zip › File2/Confidence_VeryHigh_Taxonomy/83478_out.krona.html]

Javascript must be enabled to view this page.

members
magnitude
magnitudeUnassigned
count
unassigned
taxon
rank

83478\_out

53

53
2
superkingdom

53
phylum
1239

53
class
186801

53
order
186802

216572
family
9

459786
genus
9

1897011
species

SRS012849\_contig\_number\_29731SRS012969\_contig\_number\_contig-100\_4253.221254SRS015782\_contig\_number\_contig-100\_13859.148257SRS015794\_contig\_number\_contig-100\_1216.91050SRS018656\_contig\_number\_4132SRS019808\_contig\_number\_12554SRS023526\_contig\_number\_28269SRS024435\_contig\_number\_18050SRS045004\_contig\_number\_25961
9

1898207
species
42

SRS011302\_contig\_number\_24098SRS012273\_contig\_number\_37204SRS013940\_contig\_number\_14830SRS013951\_contig\_number\_34205SRS014235\_contig\_number\_contig-100\_21606.155230SRS014459\_contig\_number\_contig-100\_42073.94579SRS014923\_contig\_number\_37971SRS014979\_contig\_number\_3263SRS015578\_contig\_number\_33784SRS015782\_contig\_number\_contig-100\_15329.176872SRS015960\_contig\_number\_contig-100\_14311.92115SRS017521\_contig\_number\_3222SRS018623\_contig\_number\_contig-100\_32012.79535SRS019601\_contig\_number\_contig-100\_212.151765SRS022071\_contig\_number\_contig-100\_927.152219SRS022524\_contig\_number\_5650SRS045826\_contig\_number\_contig-100\_15303.15304SRS047433\_contig\_number\_12033SRS048060\_contig\_number\_contig-100\_12028.52057SRS050752\_contig\_number\_21148SRS050925\_contig\_number\_contig-100\_11011.119323SRS056519\_contig\_number\_13282SRS063518\_contig\_number\_16452SRS063985\_contig\_number\_10518SRS065504\_contig\_number\_contig-100\_20488.133981SRS075078\_contig\_number\_14443SRS077194\_contig\_number\_20270SRS097958\_contig\_number\_contig-100\_993.994SRS098717\_contig\_number\_12237SRS1041033\_contig\_number\_21207SRS1041091\_contig\_number\_19858SRS1041136\_contig\_number\_16618SRS104636\_contig\_number\_2269SRS1054691\_contig\_number\_9652SRS1055022\_contig\_number\_contig-100\_16241.53918SRS140492\_contig\_number\_21891SRS144135\_contig\_number\_contig-100\_16861.144915SRS147139\_contig\_number\_44260SRS148091\_contig\_number\_contig-100\_35845.35846SRS148159\_contig\_number\_contig-100\_5490.239598SRS893270\_contig\_number\_contig-100\_12624.92914SRS893373\_contig\_number\_14126

1950935
species

SRS043411\_contig\_number\_contig-100\_3667.49931SRS098881\_contig\_number\_contig-100\_34845.84376
2
