# Supplementary material for: MetaRibo-Seq measures translation in microbiomes
Source: Nat Commun. 2020 Jun 29;11:3268. doi: 10.1038/s41467-020-17081-z (PMC7324362; doi:10.1038/s41467-020-17081-z)
Supplement: Supplementary file 10 — Supplementary Data 7 [file 41467_2020_17081_MOESM10_ESM.zip › File2/Confidence_VeryHigh_Taxonomy/283329_out.krona.html]

Javascript must be enabled to view this page.

members
magnitude
magnitudeUnassigned
count
unassigned
taxon
rank

283329\_out

4

superkingdom
2
4

1239
phylum
4

class
186801
4

186802
order
4

186803
family
4

2
1407607
genus

1150298
species
2

SRS049773\_contig\_number\_27801SRS1055043\_contig\_number\_8244

genus
841
1

1

SRS144135\_contig\_number\_32722
360807
species

1
572511
genus

1

SRS144537\_contig\_number\_27749
1262757
species
