# Supplementary material for: MetaRibo-Seq measures translation in microbiomes
Source: Nat Commun. 2020 Jun 29;11:3268. doi: 10.1038/s41467-020-17081-z (PMC7324362; doi:10.1038/s41467-020-17081-z)
Supplement: Supplementary file 10 — Supplementary Data 7 [file 41467_2020_17081_MOESM10_ESM.zip › File2/Confidence_VeryHigh_Taxonomy/184061_out.krona.html]

Javascript must be enabled to view this page.

members
magnitude
magnitudeUnassigned
count
unassigned
taxon
rank

184061\_out

4

superkingdom
2759
1

1
4751
kingdom

subkingdom
451864
1

1
phylum
5204

subphylum
29000
1

1
162484
class

1
order
5258


SRS065397\_contig\_number\_2355
27352
family
1

3
superkingdom
2

phylum
1239
3

class
186801
3

3
order
186802

2
186803
family

1
841
genus

species
301302

SRS020328\_contig\_number\_33207
1

1
33042
genus

species
2302943

SRS146813\_contig\_number\_5168
1

541000
family
1

292632
genus
1

species
1897022

SRS018541\_contig\_number\_contig-100\_154.94414
1
