# Supplementary material for: MetaRibo-Seq measures translation in microbiomes
Source: Nat Commun. 2020 Jun 29;11:3268. doi: 10.1038/s41467-020-17081-z (PMC7324362; doi:10.1038/s41467-020-17081-z)
Supplement: Supplementary file 10 — Supplementary Data 7 [file 41467_2020_17081_MOESM10_ESM.zip › File2/Confidence_VeryHigh_Taxonomy/87415_out.krona.html]

Javascript must be enabled to view this page.

members
magnitude
magnitudeUnassigned
count
unassigned
taxon
rank

87415\_out

16

16
superkingdom
2

1239
phylum
16

class
16
186801

186802
16
order

541000
family
16

1263
genus
16

40518

SRS015663\_contig\_number\_contig-100\_635.201200SRS024331\_contig\_number\_contig-100\_696.204306SRS051031\_contig\_number\_2169SRS054905\_contig\_number\_16233SRS065504\_contig\_number\_1873SRS075821\_contig\_number\_22234SRS098571\_contig\_number\_66112SRS104084\_contig\_number\_34SRS104912\_contig\_number\_3868SRS1055043\_contig\_number\_1616SRS1055099\_contig\_number\_14932SRS144183\_contig\_number\_22262SRS147346\_contig\_number\_46570SRS148424\_contig\_number\_contig-100\_621.162166SRS893369\_contig\_number\_contig-100\_696.72528SRS893383\_contig\_number\_2053
16
species
