# Supplementary material for: MetaRibo-Seq measures translation in microbiomes
Source: Nat Commun. 2020 Jun 29;11:3268. doi: 10.1038/s41467-020-17081-z (PMC7324362; doi:10.1038/s41467-020-17081-z)
Supplement: Supplementary file 10 — Supplementary Data 7 [file 41467_2020_17081_MOESM10_ESM.zip › File2/Confidence_VeryHigh_Taxonomy/35356_out.krona.html]

Javascript must be enabled to view this page.

members
magnitude
magnitudeUnassigned
count
unassigned
taxon
rank

35356\_out

8

8
2
superkingdom

phylum
8
1239

class
186801
8

186802
8
order

186804
6
family

genus
3
1505657

species
261299
3

SRS015854\_contig\_number\_contig-100\_6492.109512SRS017521\_contig\_number\_contig-100\_1126.145752SRS143372\_contig\_number\_contig-100\_17995.56961

genus
1501226
3

3
1776391

SRS015578\_contig\_number\_contig-100\_169.198700SRS019068\_contig\_number\_112121SRS142503\_contig\_number\_contig-100\_12157.103998
species

family
2
186803

2
841
genus

species
2
360807

SRS023346\_contig\_number\_8007SRS098827\_contig\_number\_3348
