# Supplementary material for: MetaRibo-Seq measures translation in microbiomes
Source: Nat Commun. 2020 Jun 29;11:3268. doi: 10.1038/s41467-020-17081-z (PMC7324362; doi:10.1038/s41467-020-17081-z)
Supplement: Supplementary file 10 — Supplementary Data 7 [file 41467_2020_17081_MOESM10_ESM.zip › File2/Confidence_VeryHigh_Taxonomy/334198_out.krona.html]

Javascript must be enabled to view this page.

members
magnitude
magnitudeUnassigned
count
unassigned
taxon
rank

334198\_out

10

superkingdom
2
10

10
976
phylum

class
200643
10

10
order
171549

family
2005473
3


SRS049446\_contig\_number\_10804SRS098073\_contig\_number\_16317SRS149325\_contig\_number\_11517
3
species
1807756

family
815
3

816
genus
3

1

SRS024435\_contig\_number\_contig-100\_208.244005
species
376805


SRS013800\_contig\_number\_12789SRS144537\_contig\_number\_1219
2
species
1262741

family
171552
4

838
genus
3

59823
species
1

SRS049959\_contig\_number\_22606


SRS104912\_contig\_number\_3881
1
1262924
species

1

SRS063370\_contig\_number\_10098
159272
species

1

SRS048164\_contig\_number\_32710
species
2049047
