# Supplementary material for: MetaRibo-Seq measures translation in microbiomes
Source: Nat Commun. 2020 Jun 29;11:3268. doi: 10.1038/s41467-020-17081-z (PMC7324362; doi:10.1038/s41467-020-17081-z)
Supplement: Supplementary file 10 — Supplementary Data 7 [file 41467_2020_17081_MOESM10_ESM.zip › File2/Confidence_VeryHigh_Taxonomy/160594_out.krona.html]

Javascript must be enabled to view this page.

members
magnitude
magnitudeUnassigned
count
unassigned
taxon
rank

160594\_out

19

2
superkingdom
19

phylum
201174
19

19
1760
class

19
order
85004

19
family
31953

19
genus
1678
2

SRS105082\_contig\_number\_contig-100\_6031.116917SRS144183\_contig\_number\_23053

12
1685
species

SRS016753\_contig\_number\_contig-100\_42.63948SRS017103\_contig\_number\_4647SRS020328\_contig\_number\_contig-100\_31028.102948SRS042966\_contig\_number\_contig-100\_1334.82008SRS045739\_contig\_number\_8979SRS074670\_contig\_number\_7764SRS075716\_contig\_number\_contig-100\_3654.38710SRS075975\_contig\_number\_contig-100\_932.932SRS075977\_contig\_number\_contig-100\_366.366SRS075979\_contig\_number\_contig-100\_56.56SRS1041031\_contig\_number\_11650SRS1041140\_contig\_number\_6146


SRS049773\_contig\_number\_15244
1
28026
species

4
species
216816

SRS018656\_contig\_number\_9124SRS023715\_contig\_number\_19192SRS049402\_contig\_number\_contig-100\_2688.94710
3

subspecies
1679
1

SRS140492\_contig\_number\_32968
