# Supplementary material for: MetaRibo-Seq measures translation in microbiomes
Source: Nat Commun. 2020 Jun 29;11:3268. doi: 10.1038/s41467-020-17081-z (PMC7324362; doi:10.1038/s41467-020-17081-z)
Supplement: Supplementary file 10 — Supplementary Data 7 [file 41467_2020_17081_MOESM10_ESM.zip › File2/Confidence_VeryHigh_Taxonomy/180097_out.krona.html]

Javascript must be enabled to view this page.

members
magnitude
magnitudeUnassigned
count
unassigned
taxon
rank

180097\_out

20

2
19
superkingdom

1239
1
phylum

1
class
186801

1
order
186802

family
1
31979

1
genus
1485

1262793
1

SRS049995\_contig\_number\_31230
species

phylum
18
976

200643
class
18

18
order
171549

815
18
family

18
1
genus

SRS1055043\_contig\_number\_13381
816

1

SRS014979\_contig\_number\_26333
species
28116

2292002
species

SRS012273\_contig\_number\_13774
1

species

SRS013940\_contig\_number\_20437SRS018836\_contig\_number\_contig-100\_432.163513SRS019968\_contig\_number\_contig-100\_5815.103559SRS021948\_contig\_number\_contig-100\_5595.176154SRS046369\_contig\_number\_73SRS056273\_contig\_number\_22924SRS075078\_contig\_number\_contig-100\_428.197137SRS076756\_contig\_number\_20160SRS143895\_contig\_number\_38843SRS144506\_contig\_number\_37927SRS144537\_contig\_number\_23920SRS148196\_contig\_number\_31928
12
329854

1263048

SRS013940\_contig\_number\_contig-100\_16435.16436
species
1

371601
2

SRS065504\_contig\_number\_31843SRS144183\_contig\_number\_32534
species

1

SRS1055067\_contig\_number\_9032
