# Supplementary material for: MetaRibo-Seq measures translation in microbiomes
Source: Nat Commun. 2020 Jun 29;11:3268. doi: 10.1038/s41467-020-17081-z (PMC7324362; doi:10.1038/s41467-020-17081-z)
Supplement: Supplementary file 10 — Supplementary Data 7 [file 41467_2020_17081_MOESM10_ESM.zip › File2/Confidence_VeryHigh_Taxonomy/39771_out.krona.html]

Javascript must be enabled to view this page.

members
magnitude
magnitudeUnassigned
count
unassigned
taxon
rank

39771\_out

22

superkingdom
22
2

22
1239
phylum

class
186801
13

186802
13
order

541000
4
family

1
1898205

SRS049773\_contig\_number\_contig-100\_52.202378
species

1263
1
genus

species

SRS014412\_contig\_number\_8047
1
1262952

species

SRS049959\_contig\_number\_22048SRS144537\_contig\_number\_49716
1952413
2

6
186806
family

1730
6
genus

1262886
6

SRS042628\_contig\_number\_17420SRS051882\_contig\_number\_contig-100\_4297.121153SRS063040\_contig\_number\_contig-100\_7.260763SRS097958\_contig\_number\_20543SRS144714\_contig\_number\_29923SRS147977\_contig\_number\_contig-100\_437.87806
species

species
1
1950932

SRS142503\_contig\_number\_contig-100\_29241.72791

family
186803
2

genus
1506553
2


SRS147557\_contig\_number\_33589SRS893369\_contig\_number\_contig-100\_499.66185
2
1946707
species

species

SRS022071\_contig\_number\_contig-100\_1220.233507SRS062701\_contig\_number\_7613SRS064276\_contig\_number\_41303SRS065504\_contig\_number\_37897SRS1041033\_contig\_number\_26270SRS104975\_contig\_number\_20033SRS143780\_contig\_number\_5624SRS144183\_contig\_number\_35420SRS148721\_contig\_number\_52755
9
1263019
