# Supplementary material for: MetaRibo-Seq measures translation in microbiomes
Source: Nat Commun. 2020 Jun 29;11:3268. doi: 10.1038/s41467-020-17081-z (PMC7324362; doi:10.1038/s41467-020-17081-z)
Supplement: Supplementary file 10 — Supplementary Data 7 [file 41467_2020_17081_MOESM10_ESM.zip › File2/Confidence_VeryHigh_Taxonomy/90368_out.krona.html]

Javascript must be enabled to view this page.

members
magnitude
magnitudeUnassigned
count
unassigned
taxon
rank

90368\_out

9

2
superkingdom
9

9
phylum
1239

class
8
186801

1

SRS1041033\_contig\_number\_6664
3
order
186802


SRS012849\_contig\_number\_28764
1950906
species
1

186807
1
family

genus
1
2282740

1
species
265477

SRS024435\_contig\_number\_contig-100\_10213.191560


SRS019068\_contig\_number\_10063SRS104400\_contig\_number\_43789SRS144506\_contig\_number\_contig-100\_50890.104009SRS147271\_contig\_number\_contig-100\_5805.44677SRS148424\_contig\_number\_contig-100\_34750.89090
2044939
species
5

1263002

SRS077194\_contig\_number\_18347
1
species
