# Supplementary material for: MetaRibo-Seq measures translation in microbiomes
Source: Nat Commun. 2020 Jun 29;11:3268. doi: 10.1038/s41467-020-17081-z (PMC7324362; doi:10.1038/s41467-020-17081-z)
Supplement: Supplementary file 10 — Supplementary Data 7 [file 41467_2020_17081_MOESM10_ESM.zip › File2/Confidence_VeryHigh_Taxonomy/3483_out.krona.html]

Javascript must be enabled to view this page.

members
magnitude
magnitudeUnassigned
count
unassigned
taxon
rank

3483\_out

8

superkingdom
2
8

7
1239
phylum

7
186801
class

order
6
186802

216572
2
family

2
459786
genus

2
1945593
species

SRS014235\_contig\_number\_contig-100\_4536.168386SRS015854\_contig\_number\_contig-100\_9557.98853

family
1
31979

genus
1
1981033

2086584
1

SRS013098\_contig\_number\_contig-100\_6277.207328
species

family
424536
3

270497
3
genus

species

SRS013965\_contig\_number\_28654SRS049959\_contig\_number\_24165SRS144537\_contig\_number\_28148
3
1946247


SRS017916\_contig\_number\_15805
species
2044939
1

201174
1
phylum

class
1
84998

order
1
84999

family
84107
1

genus
102106
1

species

SRS015782\_contig\_number\_21423
1
74426
