# Supplementary material for: MetaRibo-Seq measures translation in microbiomes
Source: Nat Commun. 2020 Jun 29;11:3268. doi: 10.1038/s41467-020-17081-z (PMC7324362; doi:10.1038/s41467-020-17081-z)
Supplement: Supplementary file 10 — Supplementary Data 7 [file 41467_2020_17081_MOESM10_ESM.zip › File2/Confidence_VeryHigh_Taxonomy/63357_out.krona.html]

Javascript must be enabled to view this page.

members
magnitude
magnitudeUnassigned
count
unassigned
taxon
rank

63357\_out

5

superkingdom
5
2

976
phylum
5

200643
5
class

171549
order
5

171552
family
4

4
1
genus

SRS017307\_contig\_number\_contig-100\_256.159870
838

165179
1
species

SRS1041116\_contig\_number\_9033

1262930

SRS049896\_contig\_number\_3369SRS144537\_contig\_number\_45000
species
2

family
1
815

1
genus
816

1262751
species

SRS049959\_contig\_number\_40686
1
