# Supplementary material for: MetaRibo-Seq measures translation in microbiomes
Source: Nat Commun. 2020 Jun 29;11:3268. doi: 10.1038/s41467-020-17081-z (PMC7324362; doi:10.1038/s41467-020-17081-z)
Supplement: Supplementary file 10 — Supplementary Data 7 [file 41467_2020_17081_MOESM10_ESM.zip › File2/Confidence_VeryHigh_Taxonomy/21298_out.krona.html]

Javascript must be enabled to view this page.

members
magnitude
magnitudeUnassigned
count
unassigned
taxon
rank

21298\_out

15

superkingdom
15
2

1239
15
phylum

class
15
186801

186802
1
order
15

SRS143342\_contig\_number\_7762

family
31979
5

5
1485
genus

59620
5

SRS053573\_contig\_number\_contig-100\_12610.12610SRS075078\_contig\_number\_contig-100\_1981.1981SRS104485\_contig\_number\_contig-100\_1283.1283SRS147425\_contig\_number\_contig-100\_215.216SRS148784\_contig\_number\_contig-100\_18621.56989
species

family
8
541000

946234
8
genus

8
1193534
species

SRS104311\_contig\_number\_24314SRS104400\_contig\_number\_13033SRS142890\_contig\_number\_15968SRS143148\_contig\_number\_contig-100\_15601.100029SRS143876\_contig\_number\_18240SRS147139\_contig\_number\_contig-100\_2883.230418SRS147766\_contig\_number\_22940SRS148511\_contig\_number\_contig-100\_14633.14634

family
1
216572

genus
1
459786

1
351091
species

SRS1054691\_contig\_number\_5904
