# Supplementary material for: MetaRibo-Seq measures translation in microbiomes
Source: Nat Commun. 2020 Jun 29;11:3268. doi: 10.1038/s41467-020-17081-z (PMC7324362; doi:10.1038/s41467-020-17081-z)
Supplement: Supplementary file 10 — Supplementary Data 7 [file 41467_2020_17081_MOESM10_ESM.zip › File2/Confidence_VeryHigh_Taxonomy/38696_out.krona.html]

Javascript must be enabled to view this page.

members
magnitude
magnitudeUnassigned
count
unassigned
taxon
rank

38696\_out

5

superkingdom
2
5

phylum
1239
5

5
186801
class

order
186802
5

186803
family
5


SRS019286\_contig\_number\_contig-100\_23130.23131SRS020394\_contig\_number\_18012SRS057478\_contig\_number\_contig-100\_912.913SRS143780\_contig\_number\_contig-100\_1282.195506SRS893341\_contig\_number\_contig-100\_1971.95097
39491
species
5
