# Supplementary material for: MetaRibo-Seq measures translation in microbiomes
Source: Nat Commun. 2020 Jun 29;11:3268. doi: 10.1038/s41467-020-17081-z (PMC7324362; doi:10.1038/s41467-020-17081-z)
Supplement: Supplementary file 10 — Supplementary Data 7 [file 41467_2020_17081_MOESM10_ESM.zip › File2/Confidence_VeryHigh_Taxonomy/439780_out.krona.html]

Javascript must be enabled to view this page.

members
magnitude
magnitudeUnassigned
count
unassigned
taxon
rank

439780\_out

29

superkingdom
2
29

29
1239
phylum

27
186801
class

27
186802
order

26
541000
family

1898205
species

SRS011134\_contig\_number\_24034SRS012849\_contig\_number\_18098SRS015663\_contig\_number\_14452SRS016203\_contig\_number\_8342SRS018541\_contig\_number\_10088SRS018836\_contig\_number\_17131SRS019161\_contig\_number\_38871SRS019445\_contig\_number\_26168SRS020394\_contig\_number\_9749SRS045004\_contig\_number\_26427SRS048164\_contig\_number\_30335SRS051031\_contig\_number\_contig-100\_392.148978SRS063518\_contig\_number\_10959SRS064757\_contig\_number\_18433SRS075773\_contig\_number\_15659SRS078176\_contig\_number\_19799SRS1041092\_contig\_number\_14940SRS1041133\_contig\_number\_20768SRS1041136\_contig\_number\_18233SRS1041141\_contig\_number\_13278SRS1041145\_contig\_number\_37590SRS1055083\_contig\_number\_8435SRS142503\_contig\_number\_43562SRS143895\_contig\_number\_811SRS144537\_contig\_number\_28902SRS971276\_contig\_number\_19150
26

species
1898207

SRS017916\_contig\_number\_16096
1

2

SRS147346\_contig\_number\_56902SRS149784\_contig\_number\_6706
species
1263007
