# Supplementary material for: MetaRibo-Seq measures translation in microbiomes
Source: Nat Commun. 2020 Jun 29;11:3268. doi: 10.1038/s41467-020-17081-z (PMC7324362; doi:10.1038/s41467-020-17081-z)
Supplement: Supplementary file 10 — Supplementary Data 7 [file 41467_2020_17081_MOESM10_ESM.zip › File2/Confidence_VeryHigh_Taxonomy/32005_out.krona.html]

Javascript must be enabled to view this page.

members
magnitude
magnitudeUnassigned
count
unassigned
taxon
rank

32005\_out

6

superkingdom
2
6

6
1239
phylum

class
186801
5

1
186802

SRS013800\_contig\_number\_contig-100\_29466.29466
order
5

31979
family
1

1
1485
genus

2293036
species
1

SRS014235\_contig\_number\_contig-100\_7153.209966

family
186803
3

species
39491
2

SRS013940\_contig\_number\_20034SRS014412\_contig\_number\_contig-100\_10123.93677

1

SRS011134\_contig\_number\_36981
species
1898203

class
1737404
1

1588751
species

SRS013542\_contig\_number\_50
1
