# Supplementary material for: MetaRibo-Seq measures translation in microbiomes
Source: Nat Commun. 2020 Jun 29;11:3268. doi: 10.1038/s41467-020-17081-z (PMC7324362; doi:10.1038/s41467-020-17081-z)
Supplement: Supplementary file 10 — Supplementary Data 7 [file 41467_2020_17081_MOESM10_ESM.zip › File2/Confidence_VeryHigh_Taxonomy/8032_out.krona.html]

Javascript must be enabled to view this page.

members
magnitude
magnitudeUnassigned
count
unassigned
taxon
rank

8032\_out

6

2
superkingdom
6

phylum
1239
1

1
186801
class

1
186802
order

1
541000
family

1
1898205

SRS024132\_contig\_number\_30193
species

phylum
976
5

200643
class
5

171549
order
5

family
171550
2

2049048

SRS019808\_contig\_number\_1923SRS104912\_contig\_number\_contig-100\_2378.122301
species
2

family
815
2

816
genus
2

species

SRS017701\_contig\_number\_9065SRS049959\_contig\_number\_contig-100\_19917.150479
1262748
2

1
171552
family

genus
838
1

228604
species

SRS144194\_contig\_number\_889
1
