# Supplementary material for: MetaRibo-Seq measures translation in microbiomes
Source: Nat Commun. 2020 Jun 29;11:3268. doi: 10.1038/s41467-020-17081-z (PMC7324362; doi:10.1038/s41467-020-17081-z)
Supplement: Supplementary file 10 — Supplementary Data 7 [file 41467_2020_17081_MOESM10_ESM.zip › File2/Confidence_VeryHigh_Taxonomy/11139_out.krona.html]

Javascript must be enabled to view this page.

members
magnitude
magnitudeUnassigned
count
unassigned
taxon
rank

11139\_out

4

superkingdom
4
2

4
976
phylum

class
4
200643

4
171549
order

4
815
family

3

SRS024492\_contig\_number\_13752SRS024625\_contig\_number\_8992SRS1041118\_contig\_number\_contig-100\_21567.60698
genus
816
4

species
246787
1

SRS021153\_contig\_number\_301
