# Supplementary material for: MetaRibo-Seq measures translation in microbiomes
Source: Nat Commun. 2020 Jun 29;11:3268. doi: 10.1038/s41467-020-17081-z (PMC7324362; doi:10.1038/s41467-020-17081-z)
Supplement: Supplementary file 10 — Supplementary Data 7 [file 41467_2020_17081_MOESM10_ESM.zip › File2/Confidence_VeryHigh_Taxonomy/358384_out.krona.html]

Javascript must be enabled to view this page.

members
magnitude
magnitudeUnassigned
count
unassigned
taxon
rank

358384\_out

31

2
superkingdom
31

32066
phylum
1

1
class
203490

203491
order
1

family
1129771
1

1
genus
32067


SRS104087\_contig\_number\_805
1
species
712357

30
phylum
1239

29
class
186801

29
186802
order

29
family
186803

29

SRS014313\_contig\_number\_contig-100\_649.53230SRS014613\_contig\_number\_15157SRS014736\_contig\_number\_9465SRS014855\_contig\_number\_contig-100\_1329.98430SRS014948\_contig\_number\_2970SRS018623\_contig\_number\_21136SRS019267\_contig\_number\_contig-100\_568.63791SRS021484\_contig\_number\_9357SRS022137\_contig\_number\_11570SRS044535\_contig\_number\_24515SRS047044\_contig\_number\_18460SRS048060\_contig\_number\_contig-100\_1104.84666SRS048870\_contig\_number\_4092SRS050998\_contig\_number\_17382SRS058723\_contig\_number\_588SRS058723\_contig\_number\_3126SRS064276\_contig\_number\_38526SRS077335\_contig\_number\_6907SRS1041036\_contig\_number\_2651SRS1041091\_contig\_number\_contig-100\_1393.126420SRS1041144\_contig\_number\_6804SRS104485\_contig\_number\_2148SRS1055049\_contig\_number\_4054SRS143342\_contig\_number\_14126SRS144362\_contig\_number\_25043SRS146764\_contig\_number\_33407SRS147377\_contig\_number\_11932SRS147614\_contig\_number\_5144SRS893341\_contig\_number\_16474
39491
species

909932
class
1

order
1843488
1

1
909930
family

1
genus
33024

1

SRS019496\_contig\_number\_contig-100\_16626.16627
626940
species
