# Supplementary material for: MetaRibo-Seq measures translation in microbiomes
Source: Nat Commun. 2020 Jun 29;11:3268. doi: 10.1038/s41467-020-17081-z (PMC7324362; doi:10.1038/s41467-020-17081-z)
Supplement: Supplementary file 10 — Supplementary Data 7 [file 41467_2020_17081_MOESM10_ESM.zip › File2/Confidence_VeryHigh_Taxonomy/31465_out.krona.html]

Javascript must be enabled to view this page.

members
magnitude
magnitudeUnassigned
count
unassigned
taxon
rank

31465\_out

17

17
superkingdom
2

phylum
1239
17


SRS011239\_contig\_number\_contig-100\_20881.62454SRS013521\_contig\_number\_contig-100\_13732.13732SRS013638\_contig\_number\_18744SRS014683\_contig\_number\_contig-100\_26004.74233
species
1262992
4

186801
class
13

13
order
186802

family
216572
13

459786
genus
13

13
1945593
species

SRS013098\_contig\_number\_40398SRS049896\_contig\_number\_10119SRS055017\_contig\_number\_contig-100\_15249.151270SRS063518\_contig\_number\_7478SRS143417\_contig\_number\_12679SRS143598\_contig\_number\_22477SRS143876\_contig\_number\_25191SRS144183\_contig\_number\_4615SRS144603\_contig\_number\_contig-100\_139.43785SRS147271\_contig\_number\_27897SRS147346\_contig\_number\_48861SRS892882\_contig\_number\_contig-100\_315.90121SRS893373\_contig\_number\_16643
