# Supplementary material for: MetaRibo-Seq measures translation in microbiomes
Source: Nat Commun. 2020 Jun 29;11:3268. doi: 10.1038/s41467-020-17081-z (PMC7324362; doi:10.1038/s41467-020-17081-z)
Supplement: Supplementary file 10 — Supplementary Data 7 [file 41467_2020_17081_MOESM10_ESM.zip › File2/Confidence_VeryHigh_Taxonomy/188891_out.krona.html]

Javascript must be enabled to view this page.

members
magnitude
magnitudeUnassigned
count
unassigned
taxon
rank

188891\_out

179
1

SRS054352\_contig\_number\_5617

178
superkingdom
2

976
phylum
178

178
class
200643

171549
178
order

family
176
171550

genus
176
239759

species
1

SRS045826\_contig\_number\_17482
1118061

28117
175
species

SRS011134\_contig\_number\_40657SRS011239\_contig\_number\_27631SRS011302\_contig\_number\_28222SRS012273\_contig\_number\_24292SRS012969\_contig\_number\_39726SRS013476\_contig\_number\_39961SRS013521\_contig\_number\_10484SRS013638\_contig\_number\_17097SRS013800\_contig\_number\_20863SRS013951\_contig\_number\_40815SRS014287\_contig\_number\_16794SRS014412\_contig\_number\_17746SRS014948\_contig\_number\_21573SRS015065\_contig\_number\_36042SRS015264\_contig\_number\_8351SRS015663\_contig\_number\_35798SRS015782\_contig\_number\_27270SRS015890\_contig\_number\_12326SRS016095\_contig\_number\_30920SRS016897\_contig\_number\_1704SRS017103\_contig\_number\_34377SRS017191\_contig\_number\_20100SRS017307\_contig\_number\_29324SRS017433\_contig\_number\_12442SRS017622\_contig\_number\_646SRS017701\_contig\_number\_19780SRS018133\_contig\_number\_698SRS018427\_contig\_number\_contig-100\_919.187083SRS018541\_contig\_number\_10051SRS018623\_contig\_number\_18434SRS018888\_contig\_number\_4078SRS018984\_contig\_number\_25421SRS019161\_contig\_number\_40005SRS019267\_contig\_number\_8751SRS019286\_contig\_number\_6122SRS019381\_contig\_number\_17875SRS019445\_contig\_number\_37041SRS019685\_contig\_number\_38385SRS019808\_contig\_number\_20870SRS020233\_contig\_number\_15363SRS020271\_contig\_number\_3851SRS020508\_contig\_number\_741SRS020622\_contig\_number\_1704SRS021219\_contig\_number\_12131SRS022071\_contig\_number\_37702SRS022137\_contig\_number\_20808SRS022524\_contig\_number\_15475SRS022609\_contig\_number\_43552SRS023715\_contig\_number\_26024SRS023971\_contig\_number\_3952SRS024132\_contig\_number\_51932SRS024331\_contig\_number\_14954SRS024435\_contig\_number\_5274SRS043667\_contig\_number\_contig-100\_1664.1664SRS043701\_contig\_number\_16218SRS045004\_contig\_number\_10207SRS045195\_contig\_number\_contig-100\_527.66092SRS045244\_contig\_number\_2802SRS045645\_contig\_number\_26427SRS046717\_contig\_number\_10950SRS047044\_contig\_number\_17782SRS048060\_contig\_number\_11366SRS049712\_contig\_number\_contig-100\_822.135401SRS049773\_contig\_number\_32447SRS050026\_contig\_number\_9832SRS050299\_contig\_number\_12722SRS050422\_contig\_number\_36541SRS050520\_contig\_number\_1267SRS050801\_contig\_number\_contig-100\_225.35687SRS050941\_contig\_number\_20423SRS050998\_contig\_number\_contig-100\_1507.109588SRS051031\_contig\_number\_35986SRS052576\_contig\_number\_703SRS053087\_contig\_number\_2704SRS053398\_contig\_number\_contig-100\_430.119417SRS053649\_contig\_number\_21107SRS054059\_contig\_number\_4832SRS055982\_contig\_number\_7090SRS057480\_contig\_number\_10581SRS058145\_contig\_number\_contig-100\_1081.35684SRS058770\_contig\_number\_17206SRS062701\_contig\_number\_7229SRS063040\_contig\_number\_33474SRS063370\_contig\_number\_5399SRS063489\_contig\_number\_7540SRS063518\_contig\_number\_35494SRS065176\_contig\_number\_16618SRS074670\_contig\_number\_8849SRS075773\_contig\_number\_38042SRS075878\_contig\_number\_contig-100\_740.147037SRS075963\_contig\_number\_contig-100\_5.22715SRS075984\_contig\_number\_14278SRS077024\_contig\_number\_contig-100\_1680.81034SRS077086\_contig\_number\_13624SRS077127\_contig\_number\_16815SRS077294\_contig\_number\_14037SRS077392\_contig\_number\_43111SRS077641\_contig\_number\_contig-100\_1195.131271SRS077849\_contig\_number\_30941SRS078176\_contig\_number\_18796SRS078242\_contig\_number\_30173SRS078419\_contig\_number\_36865SRS078665\_contig\_number\_7693SRS097920\_contig\_number\_27604SRS098061\_contig\_number\_11645SRS098620\_contig\_number\_8657SRS098655\_contig\_number\_9338SRS100907\_contig\_number\_contig-100\_295.68831SRS101376\_contig\_number\_26755SRS101433\_contig\_number\_contig-100\_1140.96175SRS103987\_contig\_number\_15196SRS104036\_contig\_number\_7957SRS1041033\_contig\_number\_contig-100\_962.156500SRS1041037\_contig\_number\_contig-100\_196.101234SRS1041095\_contig\_number\_contig-100\_1352.1353SRS1041130\_contig\_number\_1579SRS1041133\_contig\_number\_28081SRS1041134\_contig\_number\_15111SRS1041142\_contig\_number\_9166SRS1041143\_contig\_number\_2098SRS1041145\_contig\_number\_39226SRS1041147\_contig\_number\_4189SRS1041157\_contig\_number\_5197SRS1041164\_contig\_number\_5601SRS104327\_contig\_number\_5987SRS104636\_contig\_number\_17148SRS104912\_contig\_number\_20446SRS104975\_contig\_number\_24935SRS105082\_contig\_number\_1493SRS105153\_contig\_number\_4504SRS1054691\_contig\_number\_827SRS1054716\_contig\_number\_11395SRS1055022\_contig\_number\_contig-100\_422.122849SRS1055034\_contig\_number\_contig-100\_1171.83898SRS1055038\_contig\_number\_5107SRS1055076\_contig\_number\_10112SRS1055083\_contig\_number\_11002SRS140513\_contig\_number\_10030SRS142542\_contig\_number\_12801SRS143148\_contig\_number\_20233SRS143342\_contig\_number\_20765SRS143372\_contig\_number\_8832SRS143523\_contig\_number\_5937SRS143876\_contig\_number\_18012SRS143895\_contig\_number\_32391SRS144297\_contig\_number\_14588SRS144362\_contig\_number\_24780SRS144506\_contig\_number\_5210SRS144603\_contig\_number\_17098SRS144714\_contig\_number\_20556SRS145308\_contig\_number\_4544SRS145497\_contig\_number\_18691SRS146812\_contig\_number\_49223SRS147346\_contig\_number\_15093SRS147425\_contig\_number\_3725SRS148196\_contig\_number\_40106SRS148253\_contig\_number\_5648SRS148424\_contig\_number\_7727SRS148784\_contig\_number\_28533SRS148817\_contig\_number\_13091SRS148874\_contig\_number\_8202SRS149075\_contig\_number\_16560SRS149784\_contig\_number\_27219SRS893170\_contig\_number\_11SRS893172\_contig\_number\_8589SRS893187\_contig\_number\_11532SRS893230\_contig\_number\_9528SRS893252\_contig\_number\_6311SRS893270\_contig\_number\_10809SRS893279\_contig\_number\_16915SRS893358\_contig\_number\_contig-100\_599.82524SRS893369\_contig\_number\_11377SRS893378\_contig\_number\_23511SRS893380\_contig\_number\_2581SRS971275\_contig\_number\_24163

family
2
815

816
genus
2


SRS076976\_contig\_number\_896
1
species
46506

2292921
1

SRS018836\_contig\_number\_4548
species
