# Supplementary material for: MetaRibo-Seq measures translation in microbiomes
Source: Nat Commun. 2020 Jun 29;11:3268. doi: 10.1038/s41467-020-17081-z (PMC7324362; doi:10.1038/s41467-020-17081-z)
Supplement: Supplementary file 10 — Supplementary Data 7 [file 41467_2020_17081_MOESM10_ESM.zip › File2/Confidence_VeryHigh_Taxonomy/7588_out.krona.html]

Javascript must be enabled to view this page.

members
magnitude
magnitudeUnassigned
count
unassigned
taxon
rank

7588\_out

15

2
15
superkingdom

phylum
1
1224

1
class
28216

80840
1
order

995019
family
1

40544
genus
1


SRS142890\_contig\_number\_3165
40545
species
1

phylum
14
1239

186801
class
14

species
14

SRS017191\_contig\_number\_18078SRS018623\_contig\_number\_20071SRS020328\_contig\_number\_contig-100\_713.713SRS023526\_contig\_number\_contig-100\_2943.86794SRS043701\_contig\_number\_contig-100\_3014.109696SRS047014\_contig\_number\_32352SRS048164\_contig\_number\_7701SRS050998\_contig\_number\_12121SRS098644\_contig\_number\_contig-100\_35779.112207SRS143895\_contig\_number\_46517SRS144362\_contig\_number\_24906SRS149181\_contig\_number\_contig-100\_12.185291SRS893369\_contig\_number\_3168SRS971276\_contig\_number\_17554
2044939
