# Supplementary material for: MetaRibo-Seq measures translation in microbiomes
Source: Nat Commun. 2020 Jun 29;11:3268. doi: 10.1038/s41467-020-17081-z (PMC7324362; doi:10.1038/s41467-020-17081-z)
Supplement: Supplementary file 10 — Supplementary Data 7 [file 41467_2020_17081_MOESM10_ESM.zip › File2/Confidence_VeryHigh_Taxonomy/180245_out.krona.html]

Javascript must be enabled to view this page.

members
magnitude
magnitudeUnassigned
count
unassigned
taxon
rank

180245\_out

14

2
superkingdom
14

1239
phylum
14

class
186801
14

14
order
186802

14
family
31979

1485
genus
14

8

SRS011239\_contig\_number\_22440SRS012273\_contig\_number\_42848SRS022071\_contig\_number\_30595SRS049995\_contig\_number\_33946SRS063985\_contig\_number\_22363SRS078176\_contig\_number\_10874SRS148159\_contig\_number\_20025SRS971275\_contig\_number\_contig-100\_337.256158
species
1262820


SRS013951\_contig\_number\_17961SRS015663\_contig\_number\_24166SRS017916\_contig\_number\_17399SRS019161\_contig\_number\_5596SRS051031\_contig\_number\_29539SRS144135\_contig\_number\_24630
species
1262815
6
