# Supplementary material for: MetaRibo-Seq measures translation in microbiomes
Source: Nat Commun. 2020 Jun 29;11:3268. doi: 10.1038/s41467-020-17081-z (PMC7324362; doi:10.1038/s41467-020-17081-z)
Supplement: Supplementary file 10 — Supplementary Data 7 [file 41467_2020_17081_MOESM10_ESM.zip › File2/Confidence_VeryHigh_Taxonomy/31167_out.krona.html]

Javascript must be enabled to view this page.

members
magnitude
magnitudeUnassigned
count
unassigned
taxon
rank

31167\_out

62

2
62
superkingdom

976
62
phylum

62
200643
class

62
171549
order

family
171552
62

genus
62
1
577309

SRS050520\_contig\_number\_contig-100\_9346.9347

1263095
10

SRS014534\_contig\_number\_contig-100\_581.582SRS017701\_contig\_number\_14430SRS047433\_contig\_number\_16978SRS063985\_contig\_number\_26485SRS077294\_contig\_number\_contig-100\_2405.82229SRS1041130\_contig\_number\_4809SRS1041133\_contig\_number\_7011SRS1041143\_contig\_number\_8373SRS1055022\_contig\_number\_4448SRS145497\_contig\_number\_12676
species

species

SRS013800\_contig\_number\_26353SRS024435\_contig\_number\_33704SRS044535\_contig\_number\_15021SRS053649\_contig\_number\_3147SRS054352\_contig\_number\_12046SRS077641\_contig\_number\_20972SRS078665\_contig\_number\_8316SRS1041037\_contig\_number\_14062SRS1041134\_contig\_number\_13016SRS1054928\_contig\_number\_6412SRS146764\_contig\_number\_28305SRS148159\_contig\_number\_56919
12
454155


SRS012849\_contig\_number\_18154SRS013521\_contig\_number\_13040SRS015782\_contig\_number\_29175SRS016381\_contig\_number\_contig-100\_2248.2249SRS017307\_contig\_number\_26719SRS017433\_contig\_number\_14518SRS017821\_contig\_number\_26594SRS023914\_contig\_number\_12994SRS024492\_contig\_number\_12026SRS043841\_contig\_number\_contig-100\_8582.54324SRS045004\_contig\_number\_contig-100\_1636.159454SRS045645\_contig\_number\_22386SRS047044\_contig\_number\_7112SRS048164\_contig\_number\_24375SRS049712\_contig\_number\_contig-100\_7014.37297SRS049896\_contig\_number\_1086SRS049995\_contig\_number\_24203SRS050026\_contig\_number\_9411SRS053356\_contig\_number\_17648SRS055017\_contig\_number\_5252SRS063370\_contig\_number\_13105SRS064232\_contig\_number\_contig-100\_23281.23282SRS077392\_contig\_number\_19144SRS077589\_contig\_number\_6304SRS078176\_contig\_number\_17494SRS101376\_contig\_number\_14949SRS1041031\_contig\_number\_22972SRS1041091\_contig\_number\_contig-100\_14294.49726SRS1041118\_contig\_number\_1109SRS1041138\_contig\_number\_12903SRS1041140\_contig\_number\_contig-100\_511.97489SRS105082\_contig\_number\_23076SRS143070\_contig\_number\_4200SRS143181\_contig\_number\_contig-100\_27668.27668SRS143876\_contig\_number\_21010SRS147271\_contig\_number\_19400SRS147653\_contig\_number\_5957SRS893252\_contig\_number\_contig-100\_573.114416SRS893366\_contig\_number\_11755
species
454154
39
