# Supplementary material for: MetaRibo-Seq measures translation in microbiomes
Source: Nat Commun. 2020 Jun 29;11:3268. doi: 10.1038/s41467-020-17081-z (PMC7324362; doi:10.1038/s41467-020-17081-z)
Supplement: Supplementary file 10 — Supplementary Data 7 [file 41467_2020_17081_MOESM10_ESM.zip › File2/Confidence_VeryHigh_Taxonomy/158520_out.krona.html]

Javascript must be enabled to view this page.

members
magnitude
magnitudeUnassigned
count
unassigned
taxon
rank

158520\_out

11

11
superkingdom
2

1239
phylum
11

186801
class
11

11
order
186802

541000
11
family

1263
11
genus

species
2
41978

SRS058070\_contig\_number\_27794SRS097889\_contig\_number\_20320


SRS014979\_contig\_number\_30336SRS047014\_contig\_number\_contig-100\_434.75682SRS144183\_contig\_number\_23898SRS146764\_contig\_number\_9816
1638786
4
species

2292375

SRS015217\_contig\_number\_4180SRS146812\_contig\_number\_59470
2
species

40519

SRS014855\_contig\_number\_8286SRS015431\_contig\_number\_20729
2
species

species
1
1262954

SRS019068\_contig\_number\_23152
