# Supplementary material for: MetaRibo-Seq measures translation in microbiomes
Source: Nat Commun. 2020 Jun 29;11:3268. doi: 10.1038/s41467-020-17081-z (PMC7324362; doi:10.1038/s41467-020-17081-z)
Supplement: Supplementary file 10 — Supplementary Data 7 [file 41467_2020_17081_MOESM10_ESM.zip › File2/Confidence_VeryHigh_Taxonomy/350740_out.krona.html]

Javascript must be enabled to view this page.

members
magnitude
magnitudeUnassigned
count
unassigned
taxon
rank

350740\_out

29

2
superkingdom
29

29
976
phylum

28
200643
class

28
order
171549

28
815
family

816
genus
28

1
species
46506

SRS013098\_contig\_number\_19690

species
162156
1

SRS017821\_contig\_number\_12633


SRS013638\_contig\_number\_11996
1263045
species
1


SRS013521\_contig\_number\_1791SRS013800\_contig\_number\_4363SRS015794\_contig\_number\_275SRS019445\_contig\_number\_15828SRS019496\_contig\_number\_15151SRS021948\_contig\_number\_1877SRS023583\_contig\_number\_2842SRS024435\_contig\_number\_1780SRS045713\_contig\_number\_6779SRS063040\_contig\_number\_35108SRS064757\_contig\_number\_24248SRS077294\_contig\_number\_1696SRS1041129\_contig\_number\_11029SRS104693\_contig\_number\_4880SRS104975\_contig\_number\_5623SRS1054716\_contig\_number\_11053SRS140513\_contig\_number\_13251SRS147346\_contig\_number\_20130SRS149181\_contig\_number\_4711SRS893252\_contig\_number\_6206SRS893270\_contig\_number\_21485SRS893292\_contig\_number\_135SRS893295\_contig\_number\_7062SRS893300\_contig\_number\_1918
24
species
338188

47678
species
1

SRS063370\_contig\_number\_559

117743
class
1


SRS024504\_contig\_number\_contig-100\_9926.75862
order
200644
1
