# Supplementary material for: MetaRibo-Seq measures translation in microbiomes
Source: Nat Commun. 2020 Jun 29;11:3268. doi: 10.1038/s41467-020-17081-z (PMC7324362; doi:10.1038/s41467-020-17081-z)
Supplement: Supplementary file 10 — Supplementary Data 7 [file 41467_2020_17081_MOESM10_ESM.zip › File2/Confidence_VeryHigh_Taxonomy/135859_out.krona.html]

Javascript must be enabled to view this page.

members
magnitude
magnitudeUnassigned
count
unassigned
taxon
rank

135859\_out

24

2
24
superkingdom

24
phylum
1239

186801
24
class

24

SRS1041147\_contig\_number\_720SRS148721\_contig\_number\_48916
186802
order
2

186803
family
1

658087
species
1

SRS016132\_contig\_number\_5688

186806
1
family

genus
1
1730

species
1

SRS019601\_contig\_number\_contig-100\_783.157756
39485

541000
family
20

946234
genus
20


SRS015264\_contig\_number\_contig-100\_337.108788SRS023346\_contig\_number\_8589SRS046712\_contig\_number\_2851SRS049402\_contig\_number\_16866SRS051610\_contig\_number\_contig-100\_485.38578SRS053214\_contig\_number\_20888SRS054956\_contig\_number\_9303SRS055966\_contig\_number\_contig-100\_620.29210SRS063127\_contig\_number\_3264SRS077730\_contig\_number\_contig-100\_713.101339SRS097889\_contig\_number\_contig-100\_168.227822SRS098514\_contig\_number\_contig-100\_1359.5234SRS098655\_contig\_number\_14177SRS143780\_contig\_number\_5978SRS143991\_contig\_number\_28425SRS146764\_contig\_number\_29892SRS146888\_contig\_number\_15678SRS147022\_contig\_number\_14753SRS148511\_contig\_number\_21740SRS149244\_contig\_number\_3706
20
species
292800
