# Supplementary material for: MetaRibo-Seq measures translation in microbiomes
Source: Nat Commun. 2020 Jun 29;11:3268. doi: 10.1038/s41467-020-17081-z (PMC7324362; doi:10.1038/s41467-020-17081-z)
Supplement: Supplementary file 10 — Supplementary Data 7 [file 41467_2020_17081_MOESM10_ESM.zip › File2/Confidence_VeryHigh_Taxonomy/34750_out.krona.html]

Javascript must be enabled to view this page.

members
magnitude
magnitudeUnassigned
count
unassigned
taxon
rank

34750\_out

16

superkingdom
16
2

phylum
16
976

200643
class
16

171549
order
16

16
family
171552

1852368
species
16

SRS015484\_contig\_number\_1529SRS015797\_contig\_number\_contig-100\_1604.86697SRS016225\_contig\_number\_contig-100\_6627.159028SRS016225\_contig\_number\_contig-100\_6669.159070SRS017757\_contig\_number\_39111SRS018055\_contig\_number\_2315SRS020222\_contig\_number\_contig-100\_3864.3864SRS046686\_contig\_number\_4671SRS047113\_contig\_number\_53365SRS055401\_contig\_number\_8679SRS057022\_contig\_number\_575SRS104384\_contig\_number\_36366SRS1054712\_contig\_number\_contig-100\_6471.37850SRS144374\_contig\_number\_19193SRS147281\_contig\_number\_contig-100\_3318.3319SRS893274\_contig\_number\_1528
