# Supplementary material for: MetaRibo-Seq measures translation in microbiomes
Source: Nat Commun. 2020 Jun 29;11:3268. doi: 10.1038/s41467-020-17081-z (PMC7324362; doi:10.1038/s41467-020-17081-z)
Supplement: Supplementary file 10 — Supplementary Data 7 [file 41467_2020_17081_MOESM10_ESM.zip › File2/Confidence_VeryHigh_Taxonomy/338371_out.krona.html]

Javascript must be enabled to view this page.

members
magnitude
magnitudeUnassigned
count
unassigned
taxon
rank

338371\_out

10

superkingdom
2
10

phylum
976
10

10
200643
class

10
171549
order

171552
10
family

838
10
genus

2
59823
species

SRS049995\_contig\_number\_15075SRS078176\_contig\_number\_10720


SRS023715\_contig\_number\_26026SRS043667\_contig\_number\_646SRS045195\_contig\_number\_2562SRS065397\_contig\_number\_13999SRS1041031\_contig\_number\_21130SRS1041134\_contig\_number\_3144SRS1041140\_contig\_number\_10526SRS143181\_contig\_number\_10158
species
165179
8
