# Supplementary material for: MetaRibo-Seq measures translation in microbiomes
Source: Nat Commun. 2020 Jun 29;11:3268. doi: 10.1038/s41467-020-17081-z (PMC7324362; doi:10.1038/s41467-020-17081-z)
Supplement: Supplementary file 10 — Supplementary Data 7 [file 41467_2020_17081_MOESM10_ESM.zip › File2/Confidence_VeryHigh_Taxonomy/247661_out.krona.html]

Javascript must be enabled to view this page.

members
magnitude
magnitudeUnassigned
count
unassigned
taxon
rank

247661\_out

69

2
69
superkingdom

976
phylum
69

69
class
200643


SRS013951\_contig\_number\_contig-100\_8686.164797
1
171549
order
69

171550
family
1

genus
1
239759

species
1
1118061

SRS020328\_contig\_number\_33014

815
family
3

3
genus
816

162156

SRS077127\_contig\_number\_6769
1
species

821

SRS023971\_contig\_number\_1110
1
species

1
species
818

SRS077730\_contig\_number\_15495

64
family
2005525

64
genus
375288
26

SRS013800\_contig\_number\_contig-100\_3039.159358SRS015794\_contig\_number\_12506SRS023715\_contig\_number\_26899SRS024331\_contig\_number\_687SRS024388\_contig\_number\_contig-100\_639.84769SRS045004\_contig\_number\_29859SRS047044\_contig\_number\_3058SRS052697\_contig\_number\_32763SRS058070\_contig\_number\_7279SRS075773\_contig\_number\_38346SRS077392\_contig\_number\_32467SRS098571\_contig\_number\_14705SRS1041038\_contig\_number\_8192SRS1041118\_contig\_number\_contig-100\_5134.71293SRS1041136\_contig\_number\_20150SRS1041140\_contig\_number\_2402SRS1041141\_contig\_number\_contig-100\_12101.48740SRS1041144\_contig\_number\_1524SRS1054716\_contig\_number\_contig-100\_272.39401SRS1055069\_contig\_number\_13735SRS142503\_contig\_number\_54043SRS147271\_contig\_number\_contig-100\_5012.220513SRS149244\_contig\_number\_7509SRS150029\_contig\_number\_15757SRS893172\_contig\_number\_8854SRS893270\_contig\_number\_10194

2293117

SRS893231\_contig\_number\_1248
1
species

1
species

SRS021219\_contig\_number\_2189
2293115


SRS022071\_contig\_number\_32845SRS1055043\_contig\_number\_21032SRS1055099\_contig\_number\_9765
2293116
3
species

species
3

SRS1041092\_contig\_number\_8143SRS1041137\_contig\_number\_21292SRS144135\_contig\_number\_18555
46503

species
29
823

SRS012273\_contig\_number\_44791SRS013158\_contig\_number\_1305SRS015782\_contig\_number\_39234SRS017103\_contig\_number\_27346SRS017433\_contig\_number\_8885SRS018313\_contig\_number\_10854SRS018623\_contig\_number\_18108SRS019030\_contig\_number\_17161SRS019161\_contig\_number\_9064SRS020233\_contig\_number\_59516SRS021948\_contig\_number\_33543SRS022713\_contig\_number\_6897SRS045645\_contig\_number\_15539SRS045713\_contig\_number\_3391SRS050026\_contig\_number\_3338SRS053214\_contig\_number\_13620SRS057478\_contig\_number\_5969SRS074964\_contig\_number\_6451SRS077194\_contig\_number\_29486SRS078665\_contig\_number\_2372SRS1041132\_contig\_number\_1617SRS1041133\_contig\_number\_19916SRS1054928\_contig\_number\_231SRS144362\_contig\_number\_12475SRS146764\_contig\_number\_17625SRS893187\_contig\_number\_6937SRS893253\_contig\_number\_284SRS893256\_contig\_number\_2783SRS893382\_contig\_number\_contig-100\_1168.27549

species
1

SRS1041039\_contig\_number\_14811
2293114
