# Supplementary material for: MetaRibo-Seq measures translation in microbiomes
Source: Nat Commun. 2020 Jun 29;11:3268. doi: 10.1038/s41467-020-17081-z (PMC7324362; doi:10.1038/s41467-020-17081-z)
Supplement: Supplementary file 10 — Supplementary Data 7 [file 41467_2020_17081_MOESM10_ESM.zip › File2/Confidence_VeryHigh_Taxonomy/282070_out.krona.html]

Javascript must be enabled to view this page.

members
magnitude
magnitudeUnassigned
count
unassigned
taxon
rank

282070\_out

7

2

SRS058070\_contig\_number\_contig-100\_1055.189608SRS098571\_contig\_number\_30771
6
superkingdom
2

phylum
201174
4

4

SRS053356\_contig\_number\_16546SRS076929\_contig\_number\_6372
1760
2
class

2
order
85012

2
family
2004

genus
1995
2


SRS023914\_contig\_number\_14575SRS051031\_contig\_number\_26272
2
1997
species

1
superkingdom
2759

4751
kingdom
1

451864
subkingdom
1

4890
phylum
1

147538
subphylum
1

class
147550
1

subclass
222544
1

639021
order
1

1
family
81093


SRS1041145\_contig\_number\_8608
1
48558
genus
