# Supplementary material for: MetaRibo-Seq measures translation in microbiomes
Source: Nat Commun. 2020 Jun 29;11:3268. doi: 10.1038/s41467-020-17081-z (PMC7324362; doi:10.1038/s41467-020-17081-z)
Supplement: Supplementary file 10 — Supplementary Data 7 [file 41467_2020_17081_MOESM10_ESM.zip › File2/Confidence_VeryHigh_Taxonomy/155929_out.krona.html]

Javascript must be enabled to view this page.

members
magnitude
magnitudeUnassigned
count
unassigned
taxon
rank

155929\_out

167

167
2
superkingdom

phylum
1239
167

186801
167
class

order
167
186802

species

SRS097889\_contig\_number\_16755
1
1897045

172733
1

SRS048164\_contig\_number\_20826
species

541000
164
family

3
164

SRS016840\_contig\_number\_1918SRS063370\_contig\_number\_5954SRS143070\_contig\_number\_12905
216851
genus


SRS023715\_contig\_number\_19752SRS893230\_contig\_number\_contig-100\_515.81318
species
259315
2


SRS011302\_contig\_number\_1613SRS012849\_contig\_number\_36848SRS013476\_contig\_number\_9168SRS013521\_contig\_number\_9390SRS013638\_contig\_number\_contig-100\_1433.109177SRS013940\_contig\_number\_21888SRS013951\_contig\_number\_29531SRS013965\_contig\_number\_contig-100\_873.223790SRS014412\_contig\_number\_contig-100\_842.124094SRS014459\_contig\_number\_30551SRS014613\_contig\_number\_20900SRS014683\_contig\_number\_4895SRS014855\_contig\_number\_16402SRS014948\_contig\_number\_14979SRS015217\_contig\_number\_14454SRS015431\_contig\_number\_60453SRS015578\_contig\_number\_35405SRS015663\_contig\_number\_28051SRS015694\_contig\_number\_23815SRS015782\_contig\_number\_46368SRS015854\_contig\_number\_contig-100\_720.139909SRS016203\_contig\_number\_contig-100\_733.83086SRS016495\_contig\_number\_19151SRS016954\_contig\_number\_1011SRS016989\_contig\_number\_17329SRS017191\_contig\_number\_6440SRS017307\_contig\_number\_30323SRS018351\_contig\_number\_2207SRS018427\_contig\_number\_33204SRS018575\_contig\_number\_6871SRS018836\_contig\_number\_28030SRS019030\_contig\_number\_contig-100\_895.119909SRS019068\_contig\_number\_86082SRS019161\_contig\_number\_39050SRS019445\_contig\_number\_28933SRS019496\_contig\_number\_17247SRS019808\_contig\_number\_contig-100\_599.122280SRS019968\_contig\_number\_contig-100\_714.113454SRS020233\_contig\_number\_34894SRS020328\_contig\_number\_32350SRS020869\_contig\_number\_39186SRS022524\_contig\_number\_contig-100\_579.86598SRS023346\_contig\_number\_3744SRS023526\_contig\_number\_contig-100\_1359.217224SRS024075\_contig\_number\_contig-100\_279.109165SRS024132\_contig\_number\_33300SRS024331\_contig\_number\_contig-100\_198.208773SRS024435\_contig\_number\_1684SRS024663\_contig\_number\_contig-100\_477.98579SRS042628\_contig\_number\_35634SRS042966\_contig\_number\_18012SRS043001\_contig\_number\_9890SRS043701\_contig\_number\_contig-100\_126.117698SRS043701\_contig\_number\_contig-100\_332.117904SRS043768\_contig\_number\_contig-100\_751.192884SRS045004\_contig\_number\_contig-100\_103.192532SRS046369\_contig\_number\_contig-100\_539.107520SRS046502\_contig\_number\_365SRS049446\_contig\_number\_contig-100\_1033.87799SRS049712\_contig\_number\_contig-100\_781.140101SRS049959\_contig\_number\_53179SRS049995\_contig\_number\_39250SRS050422\_contig\_number\_35970SRS050925\_contig\_number\_15923SRS050941\_contig\_number\_contig-100\_1050.138858SRS051031\_contig\_number\_35250SRS051882\_contig\_number\_22508SRS052078\_contig\_number\_4476SRS052697\_contig\_number\_contig-100\_626.250996SRS054352\_contig\_number\_24345SRS054905\_contig\_number\_contig-100\_886.147338SRS054956\_contig\_number\_16741SRS055017\_contig\_number\_contig-100\_609.209007SRS056273\_contig\_number\_contig-100\_1096.195232SRS056519\_contig\_number\_13110SRS058770\_contig\_number\_contig-100\_512.157772SRS062654\_contig\_number\_4736SRS063040\_contig\_number\_3062SRS063985\_contig\_number\_11284SRS064276\_contig\_number\_4131SRS064557\_contig\_number\_contig-100\_924.127283SRS065397\_contig\_number\_contig-100\_796.91843SRS075078\_contig\_number\_contig-100\_1324.188677SRS075878\_contig\_number\_contig-100\_917.150534SRS075984\_contig\_number\_contig-100\_1249.155463SRS076756\_contig\_number\_contig-100\_1267.116207SRS076804\_contig\_number\_20351SRS076929\_contig\_number\_contig-100\_456.152459SRS076976\_contig\_number\_14458SRS077127\_contig\_number\_9547SRS077194\_contig\_number\_12991SRS077194\_contig\_number\_contig-100\_555.192575SRS077231\_contig\_number\_11431SRS077294\_contig\_number\_contig-100\_800.118051SRS077392\_contig\_number\_42855SRS077454\_contig\_number\_1278SRS077589\_contig\_number\_591SRS077641\_contig\_number\_20756SRS077730\_contig\_number\_contig-100\_490.118595SRS077849\_contig\_number\_contig-100\_1156.191207SRS078176\_contig\_number\_14732SRS078242\_contig\_number\_contig-100\_867.192997SRS097889\_contig\_number\_39113SRS097920\_contig\_number\_contig-100\_1210.186621SRS097958\_contig\_number\_17769SRS098061\_contig\_number\_contig-100\_897.94138SRS098514\_contig\_number\_contig-100\_1371.1371SRS098644\_contig\_number\_47496SRS098717\_contig\_number\_19096SRS101376\_contig\_number\_contig-100\_732.169243SRS104084\_contig\_number\_contig-100\_421.90747SRS1041037\_contig\_number\_contig-100\_960.74732SRS1041090\_contig\_number\_contig-100\_853.54696SRS1041157\_contig\_number\_6786SRS104311\_contig\_number\_43943SRS104400\_contig\_number\_57631SRS104693\_contig\_number\_contig-100\_728.92402SRS105153\_contig\_number\_36374SRS1055056\_contig\_number\_89SRS140513\_contig\_number\_2733SRS142542\_contig\_number\_contig-100\_663.166305SRS142890\_contig\_number\_contig-100\_864.244705SRS142980\_contig\_number\_contig-100\_960.46779SRS143148\_contig\_number\_contig-100\_1253.147032SRS143342\_contig\_number\_18575SRS143372\_contig\_number\_12409SRS143466\_contig\_number\_30559SRS143523\_contig\_number\_contig-100\_511.160155SRS143722\_contig\_number\_4349SRS143780\_contig\_number\_29822SRS143895\_contig\_number\_contig-100\_988.259312SRS144135\_contig\_number\_13253SRS144506\_contig\_number\_55613SRS144714\_contig\_number\_contig-100\_1138.164104SRS146812\_contig\_number\_56262SRS146813\_contig\_number\_contig-100\_1044.154000SRS147022\_contig\_number\_contig-100\_454.93988SRS147346\_contig\_number\_contig-100\_449.356261SRS147919\_contig\_number\_1049SRS147977\_contig\_number\_6804SRS148091\_contig\_number\_7743SRS148159\_contig\_number\_38095SRS148196\_contig\_number\_39446SRS148319\_contig\_number\_16147SRS148817\_contig\_number\_contig-100\_978.89585SRS148874\_contig\_number\_17201SRS149075\_contig\_number\_15922SRS149879\_contig\_number\_24105SRS893231\_contig\_number\_123SRS893279\_contig\_number\_15218SRS893292\_contig\_number\_contig-100\_419.66180SRS893327\_contig\_number\_10539SRS893358\_contig\_number\_contig-100\_976.72796SRS893373\_contig\_number\_27581SRS893378\_contig\_number\_18554SRS971275\_contig\_number\_contig-100\_516.278175SRS971276\_contig\_number\_18257
species
853
157

species

SRS012969\_contig\_number\_contig-100\_1090.234292SRS014923\_contig\_number\_contig-100\_742.247219
2
2302957

family
186803
1

genus
572511
1


SRS147653\_contig\_number\_contig-100\_271.59438
species
2292990
1
