# Supplementary material for: MetaRibo-Seq measures translation in microbiomes
Source: Nat Commun. 2020 Jun 29;11:3268. doi: 10.1038/s41467-020-17081-z (PMC7324362; doi:10.1038/s41467-020-17081-z)
Supplement: Supplementary file 10 — Supplementary Data 7 [file 41467_2020_17081_MOESM10_ESM.zip › File2/Confidence_VeryHigh_Taxonomy/156160_out.krona.html]

Javascript must be enabled to view this page.

members
magnitude
magnitudeUnassigned
count
unassigned
taxon
rank

156160\_out

4

2
4
superkingdom

1239
4
phylum

186801
class
4

4
order
186802

family
4
31979

4
genus
1485

1
species

SRS019685\_contig\_number\_37574
1262841


SRS012273\_contig\_number\_41100SRS063985\_contig\_number\_15162SRS148159\_contig\_number\_25574
1262820
species
3
