# Supplementary material for: MetaRibo-Seq measures translation in microbiomes
Source: Nat Commun. 2020 Jun 29;11:3268. doi: 10.1038/s41467-020-17081-z (PMC7324362; doi:10.1038/s41467-020-17081-z)
Supplement: Supplementary file 10 — Supplementary Data 7 [file 41467_2020_17081_MOESM10_ESM.zip › File2/Confidence_VeryHigh_Taxonomy/121997_out.krona.html]

Javascript must be enabled to view this page.

members
magnitude
magnitudeUnassigned
count
unassigned
taxon
rank

121997\_out

5

superkingdom
2
4

4
phylum
1239

186801
class
4

186802
order
4

2
family
186806

2
genus
1730


SRS147272\_contig\_number\_contig-100\_3783.3784
1
species
2293107

1

SRS147919\_contig\_number\_19214
1262881
species

2
family
186803

1235793
species
1

SRS143991\_contig\_number\_25396

1392836
species
1

SRS147377\_contig\_number\_12291

1
superkingdom
2759

1
kingdom
4751

subkingdom
451864
1

1
5204
phylum

1
29000
subphylum

class
162484
1

order
5258
1

genus
203903
1

species
203904

SRS077552\_contig\_number\_contig-100\_9896.51506
1
