# Supplementary material for: MetaRibo-Seq measures translation in microbiomes
Source: Nat Commun. 2020 Jun 29;11:3268. doi: 10.1038/s41467-020-17081-z (PMC7324362; doi:10.1038/s41467-020-17081-z)
Supplement: Supplementary file 10 — Supplementary Data 7 [file 41467_2020_17081_MOESM10_ESM.zip › File2/Confidence_VeryHigh_Taxonomy/200408_out.krona.html]

Javascript must be enabled to view this page.

members
magnitude
magnitudeUnassigned
count
unassigned
taxon
rank

200408\_out

20

superkingdom
2
19

1
976
phylum

1
200643
class

171549
1
order

1
171550
family

1
239759
genus


SRS050925\_contig\_number\_4783
species
28117
1

1239
18
phylum

class
18
186801

order
18
186802

541000
3
family

genus
3
35829


SRS016335\_contig\_number\_26692SRS148721\_contig\_number\_9723SRS893230\_contig\_number\_10751
species
3
290052

186803
14
family

genus
28050
1

species

SRS147919\_contig\_number\_14750
1
1946712

5
1898203

SRS013687\_contig\_number\_32583SRS098717\_contig\_number\_5296SRS1041112\_contig\_number\_contig-100\_1541.1542SRS143466\_contig\_number\_15121SRS147346\_contig\_number\_contig-100\_5292.354086
species

39491
1

SRS011302\_contig\_number\_contig-100\_149.171165
species

species

SRS024435\_contig\_number\_contig-100\_525.248416
1
397288

genus
6
841


SRS015578\_contig\_number\_15401SRS018313\_contig\_number\_6799SRS048981\_contig\_number\_7994SRS075021\_contig\_number\_contig-100\_13.60610SRS142890\_contig\_number\_14644
species
5
2049040

species

SRS018575\_contig\_number\_contig-100\_3604.55442
166486
1

family
186806
1

genus
1
1730

1
142586
species

SRS047014\_contig\_number\_33968

1

SRS893295\_contig\_number\_contig-100\_31414.31415
