# Supplementary material for: MetaRibo-Seq measures translation in microbiomes
Source: Nat Commun. 2020 Jun 29;11:3268. doi: 10.1038/s41467-020-17081-z (PMC7324362; doi:10.1038/s41467-020-17081-z)
Supplement: Supplementary file 10 — Supplementary Data 7 [file 41467_2020_17081_MOESM10_ESM.zip › File2/Confidence_VeryHigh_Taxonomy/385061_out.krona.html]

Javascript must be enabled to view this page.

members
magnitude
magnitudeUnassigned
count
unassigned
taxon
rank

385061\_out

18

18
2
superkingdom

phylum
18
1224

class
1236
18

order
18
91347

1
543
18

SRS971427\_contig\_number\_18530
family

genus
2
547

1
354276

SRS012849\_contig\_number\_11942
species group

species

SRS1055050\_contig\_number\_contig-100\_417.418
1
539813


SRS019601\_contig\_number\_10230SRS1055050\_contig\_number\_236SRS140492\_contig\_number\_24099
genus
14
561
3

species

SRS012273\_contig\_number\_8283SRS017191\_contig\_number\_11725SRS017521\_contig\_number\_48294SRS019068\_contig\_number\_35810SRS046502\_contig\_number\_contig-100\_4065.4066SRS049402\_contig\_number\_16971SRS064276\_contig\_number\_18621SRS077646\_contig\_number\_contig-100\_2189.2190SRS144362\_contig\_number\_31603SRS146888\_contig\_number\_14676SRS147022\_contig\_number\_12272
562
11


SRS143070\_contig\_number\_contig-100\_2479.77251
genus
570
1
