# Supplementary material for: MetaRibo-Seq measures translation in microbiomes
Source: Nat Commun. 2020 Jun 29;11:3268. doi: 10.1038/s41467-020-17081-z (PMC7324362; doi:10.1038/s41467-020-17081-z)
Supplement: Supplementary file 10 — Supplementary Data 7 [file 41467_2020_17081_MOESM10_ESM.zip › File2/Confidence_VeryHigh_Taxonomy/319842_out.krona.html]

Javascript must be enabled to view this page.

members
magnitude
magnitudeUnassigned
count
unassigned
taxon
rank

319842\_out

115

superkingdom
2
115

114
phylum
976

species
652708

SRS104711\_contig\_number\_4336
1

200643
class
112

171549
order
112

1
family
171552

838
genus
1

species
28131

SRS148771\_contig\_number\_contig-100\_1078.65956
1

family
815
111


SRS011134\_contig\_number\_contig-100\_820.293441SRS012849\_contig\_number\_8713SRS013098\_contig\_number\_49745SRS013951\_contig\_number\_contig-100\_445.231439SRS015264\_contig\_number\_5829SRS015579\_contig\_number\_contig-100\_652.135977SRS018575\_contig\_number\_contig-100\_656.64084SRS018984\_contig\_number\_20957SRS020869\_contig\_number\_contig-100\_1052.213442SRS022071\_contig\_number\_contig-100\_994.221355SRS023583\_contig\_number\_contig-100\_984.96498SRS024331\_contig\_number\_31350SRS043667\_contig\_number\_5488SRS050299\_contig\_number\_13666SRS050925\_contig\_number\_contig-100\_915.179211SRS056273\_contig\_number\_contig-100\_1170.190110SRS064757\_contig\_number\_23701SRS077392\_contig\_number\_contig-100\_1170.237019SRS1041039\_contig\_number\_contig-100\_999.146224SRS1041134\_contig\_number\_contig-100\_821.85666SRS1041137\_contig\_number\_22146SRS143181\_contig\_number\_contig-100\_646.89645SRS147139\_contig\_number\_19533SRS147557\_contig\_number\_contig-100\_1354.192302SRS148091\_contig\_number\_contig-100\_1146.86878SRS148159\_contig\_number\_contig-100\_705.326581SRS148721\_contig\_number\_contig-100\_950.294662SRS150029\_contig\_number\_contig-100\_877.110847SRS893230\_contig\_number\_8601SRS893292\_contig\_number\_contig-100\_747.62448
30
genus
111
816


SRS013215\_contig\_number\_1160SRS013940\_contig\_number\_22390SRS015794\_contig\_number\_11432SRS016018\_contig\_number\_16614SRS016381\_contig\_number\_2707SRS017701\_contig\_number\_contig-100\_1092.124978SRS017821\_contig\_number\_contig-100\_786.184154SRS018049\_contig\_number\_2508SRS019286\_contig\_number\_2687SRS022524\_contig\_number\_contig-100\_599.92522SRS023346\_contig\_number\_8481SRS023715\_contig\_number\_contig-100\_213.172480SRS024132\_contig\_number\_48891SRS024435\_contig\_number\_30653SRS024549\_contig\_number\_665SRS024663\_contig\_number\_11184SRS042628\_contig\_number\_37893SRS043001\_contig\_number\_8442SRS045195\_contig\_number\_6348SRS045713\_contig\_number\_contig-100\_976.108360SRS046717\_contig\_number\_11050SRS052078\_contig\_number\_4684SRS053649\_contig\_number\_contig-100\_1161.133719SRS057717\_contig\_number\_5842SRS063040\_contig\_number\_22732SRS075021\_contig\_number\_6108SRS077127\_contig\_number\_contig-100\_1157.109338SRS098655\_contig\_number\_10951SRS101268\_contig\_number\_1578SRS1041032\_contig\_number\_17062SRS1041038\_contig\_number\_contig-100\_39.100183SRS1041136\_contig\_number\_contig-100\_687.129014SRS104636\_contig\_number\_14208SRS140645\_contig\_number\_contig-100\_57.25854SRS142712\_contig\_number\_4184SRS143598\_contig\_number\_18665SRS145497\_contig\_number\_contig-100\_1809.114960SRS148874\_contig\_number\_3824SRS149181\_contig\_number\_9133SRS971276\_contig\_number\_29345
40
species
357276

338188
species

SRS058070\_contig\_number\_25036
1

1

SRS020233\_contig\_number\_24229
species
626929


SRS014923\_contig\_number\_44879
1
1263042
species


SRS013476\_contig\_number\_22507SRS015890\_contig\_number\_21250SRS043768\_contig\_number\_30829SRS054590\_contig\_number\_contig-100\_9.29018SRS058770\_contig\_number\_27255SRS1041112\_contig\_number\_contig-100\_494.42166SRS143523\_contig\_number\_24401SRS148511\_contig\_number\_contig-100\_913.123778SRS149879\_contig\_number\_16044
9
821
species


SRS018133\_contig\_number\_10651SRS018541\_contig\_number\_12133SRS048981\_contig\_number\_3477SRS056259\_contig\_number\_15307SRS056519\_contig\_number\_3518SRS075341\_contig\_number\_25014SRS077024\_contig\_number\_14535SRS078419\_contig\_number\_20215SRS098717\_contig\_number\_15102SRS1055034\_contig\_number\_contig-100\_204.82931SRS149784\_contig\_number\_28402
11
species
817

species
2292935

SRS014613\_contig\_number\_21423SRS017191\_contig\_number\_contig-100\_965.116865SRS020394\_contig\_number\_contig-100\_688.110786SRS022137\_contig\_number\_contig-100\_1003.124998SRS043701\_contig\_number\_16317SRS048060\_contig\_number\_9500SRS053356\_contig\_number\_35131SRS055982\_contig\_number\_18672SRS064232\_contig\_number\_4007SRS064276\_contig\_number\_40412SRS101433\_contig\_number\_contig-100\_985.101995SRS1041145\_contig\_number\_40932SRS1054691\_contig\_number\_17669SRS1055043\_contig\_number\_21276SRS1055067\_contig\_number\_6312SRS1055099\_contig\_number\_13636SRS147653\_contig\_number\_2416SRS893342\_contig\_number\_contig-100\_1063.63171
18

117743
class
1

order
200644
1

1
family
49546

1016
genus
1

1

SRS019494\_contig\_number\_1074
species
1018

1
phylum
1239

186801
class
1

1
186802
order

1
186803
family

1952172
species
1

SRS056537\_contig\_number\_48359
