# Supplementary material for: MetaRibo-Seq measures translation in microbiomes
Source: Nat Commun. 2020 Jun 29;11:3268. doi: 10.1038/s41467-020-17081-z (PMC7324362; doi:10.1038/s41467-020-17081-z)
Supplement: Supplementary file 10 — Supplementary Data 7 [file 41467_2020_17081_MOESM10_ESM.zip › File2/Confidence_VeryHigh_Taxonomy/395529_out.krona.html]

Javascript must be enabled to view this page.

members
magnitude
magnitudeUnassigned
count
unassigned
taxon
rank

395529\_out

57

2
superkingdom
57

57
phylum
1239

186801
class
57

order
3
57
186802

SRS019068\_contig\_number\_contig-100\_42814.125092SRS051882\_contig\_number\_contig-100\_34989.34989SRS077392\_contig\_number\_contig-100\_24912.24913

7
186803
family


SRS016132\_contig\_number\_1871SRS046712\_contig\_number\_400SRS051610\_contig\_number\_7441SRS077552\_contig\_number\_contig-100\_7099.48709SRS143780\_contig\_number\_27754SRS147139\_contig\_number\_5687SRS149244\_contig\_number\_contig-100\_6504.45802
7
658087
species

541000
family
45

946234
genus
45

species
1193534
2

SRS049402\_contig\_number\_9071SRS148511\_contig\_number\_11133


SRS013098\_contig\_number\_26832SRS014459\_contig\_number\_15320SRS014683\_contig\_number\_contig-100\_25762.73991SRS015217\_contig\_number\_9697SRS015264\_contig\_number\_contig-100\_13575.81397SRS016267\_contig\_number\_6053SRS016495\_contig\_number\_contig-100\_4142.4142SRS017521\_contig\_number\_contig-100\_8196.222706SRS017745\_contig\_number\_contig-100\_19307.19308SRS019601\_contig\_number\_24876SRS022713\_contig\_number\_contig-100\_8185.8185SRS023346\_contig\_number\_8360SRS043411\_contig\_number\_contig-100\_15341.61605SRS045244\_contig\_number\_contig-100\_10506.49230SRS049402\_contig\_number\_contig-100\_3798.79682SRS052078\_contig\_number\_contig-100\_19851.19852SRS053214\_contig\_number\_contig-100\_15680.92986SRS054956\_contig\_number\_contig-100\_6183.68756SRS055966\_contig\_number\_1259SRS056519\_contig\_number\_contig-100\_7375.7375SRS057478\_contig\_number\_contig-100\_7553.30299SRS058770\_contig\_number\_contig-100\_22629.100333SRS063127\_contig\_number\_1878SRS075398\_contig\_number\_contig-100\_6479.6480SRS077454\_contig\_number\_1425SRS077502\_contig\_number\_6885SRS077730\_contig\_number\_15121SRS098717\_contig\_number\_8920SRS1041090\_contig\_number\_contig-100\_27258.27259SRS1041095\_contig\_number\_contig-100\_17797.17798SRS1041147\_contig\_number\_3457SRS142712\_contig\_number\_1818SRS143876\_contig\_number\_contig-100\_11793.124283SRS143991\_contig\_number\_19389SRS146764\_contig\_number\_3585SRS146888\_contig\_number\_contig-100\_1585.84586SRS147022\_contig\_number\_7393SRS147919\_contig\_number\_contig-100\_3837.3837SRS148511\_contig\_number\_17489SRS148721\_contig\_number\_contig-100\_6894.274268SRS148874\_contig\_number\_contig-100\_5344.48684SRS893253\_contig\_number\_2658SRS893256\_contig\_number\_1813
43
292800
species

31979
family
2

1485
genus
2

species
649724
1

SRS098655\_contig\_number\_8800

species
1650661
1

SRS054590\_contig\_number\_contig-100\_10249.10249
