# Supplementary material for: MetaRibo-Seq measures translation in microbiomes
Source: Nat Commun. 2020 Jun 29;11:3268. doi: 10.1038/s41467-020-17081-z (PMC7324362; doi:10.1038/s41467-020-17081-z)
Supplement: Supplementary file 10 — Supplementary Data 7 [file 41467_2020_17081_MOESM10_ESM.zip › File2/Confidence_VeryHigh_Taxonomy/56329_out.krona.html]

Javascript must be enabled to view this page.

members
magnitude
magnitudeUnassigned
count
unassigned
taxon
rank

56329\_out

6

6
superkingdom
2

5
phylum
1239


SRS065504\_contig\_number\_31351
1
species
1263000

186801
class
4

186802
4
order

species
1
172733

SRS893366\_contig\_number\_17197

3
family
186803

265975
genus
2


SRS018610\_contig\_number\_13224
237576
1
species


SRS053603\_contig\_number\_14494
1501329
species
1

841
1
genus


SRS015663\_contig\_number\_2538
360807
species
1

1
phylum
976

200643
class
1

1
order
171549

171552
family
1

1
genus
838

species
1
655809

SRS018394\_contig\_number\_4729
