# Supplementary material for: MetaRibo-Seq measures translation in microbiomes
Source: Nat Commun. 2020 Jun 29;11:3268. doi: 10.1038/s41467-020-17081-z (PMC7324362; doi:10.1038/s41467-020-17081-z)
Supplement: Supplementary file 10 — Supplementary Data 7 [file 41467_2020_17081_MOESM10_ESM.zip › File2/Confidence_VeryHigh_Taxonomy/108660_out.krona.html]

Javascript must be enabled to view this page.

members
magnitude
magnitudeUnassigned
count
unassigned
taxon
rank

108660\_out

9

9
2
superkingdom

phylum
1224
9

class
1236
9

9
91347
order

9
family
543

9
genus
561


SRS012273\_contig\_number\_46223SRS049402\_contig\_number\_14269SRS057478\_contig\_number\_4864SRS076876\_contig\_number\_contig-100\_1547.1548SRS077127\_contig\_number\_874SRS077454\_contig\_number\_299SRS144362\_contig\_number\_7879SRS146888\_contig\_number\_15462SRS147022\_contig\_number\_14067
9
562
species
