# Supplementary material for: MetaRibo-Seq measures translation in microbiomes
Source: Nat Commun. 2020 Jun 29;11:3268. doi: 10.1038/s41467-020-17081-z (PMC7324362; doi:10.1038/s41467-020-17081-z)
Supplement: Supplementary file 10 — Supplementary Data 7 [file 41467_2020_17081_MOESM10_ESM.zip › File2/Confidence_VeryHigh_Taxonomy/250593_out.krona.html]

Javascript must be enabled to view this page.

members
magnitude
magnitudeUnassigned
count
unassigned
taxon
rank

250593\_out

4

2

SRS054590\_contig\_number\_contig-100\_14032.14032SRS076929\_contig\_number\_contig-100\_1580.1580

superkingdom
2
2

1239
2
phylum

186801
2
class

2
186802
order

family
186803
1

1
1952136
species

SRS148511\_contig\_number\_17074

family
541000
1

1263
1
genus

1
1262958
species

SRS104400\_contig\_number\_45816
