# Supplementary material for: MetaRibo-Seq measures translation in microbiomes
Source: Nat Commun. 2020 Jun 29;11:3268. doi: 10.1038/s41467-020-17081-z (PMC7324362; doi:10.1038/s41467-020-17081-z)
Supplement: Supplementary file 10 — Supplementary Data 7 [file 41467_2020_17081_MOESM10_ESM.zip › File2/Confidence_VeryHigh_Taxonomy/79822_out.krona.html]

Javascript must be enabled to view this page.

members
magnitude
magnitudeUnassigned
count
unassigned
taxon
rank

79822\_out

7

7
2
superkingdom

phylum
1239
5

5
186801
class

order
186802
5

3
family
186803

1
1164882
genus

species
467210

SRS012279\_contig\_number\_16691
1

genus
265975
2


SRS018875\_contig\_number\_19673
1
species
1501332


SRS893173\_contig\_number\_contig-100\_33950.33951
1
1501329
species

family
186806
1

1730
genus
1

1262889
species

SRS047014\_contig\_number\_contig-100\_30101.63845
1

1
family
216572

459786
genus
1


SRS1055076\_contig\_number\_contig-100\_8333.82130
1
2109687
species

2
32066
phylum

class
203490
2

203491
order
2

2
family
1129771

genus
32067
2


SRS079308\_contig\_number\_contig-100\_7519.372279
1
species
712359

species
712357

SRS018665\_contig\_number\_9780
1
