# Supplementary material for: MetaRibo-Seq measures translation in microbiomes
Source: Nat Commun. 2020 Jun 29;11:3268. doi: 10.1038/s41467-020-17081-z (PMC7324362; doi:10.1038/s41467-020-17081-z)
Supplement: Supplementary file 10 — Supplementary Data 7 [file 41467_2020_17081_MOESM10_ESM.zip › File2/Confidence_VeryHigh_Taxonomy/61848_out.krona.html]

Javascript must be enabled to view this page.

members
magnitude
magnitudeUnassigned
count
unassigned
taxon
rank

61848\_out

12

12
2
superkingdom

phylum
1239
12

class
186801
12

12
order
186802

2
family
186806

genus
1730
2

1262880
species
1

SRS077730\_contig\_number\_contig-100\_60.121040


SRS051031\_contig\_number\_36128
39496
species
1

3
family
186803


SRS023715\_contig\_number\_contig-100\_2780.2781
658085
species
1

2
572511
genus


SRS147977\_contig\_number\_13862
1
species
40520


SRS149879\_contig\_number\_contig-100\_4090.163261
1262756
species
1

family
541000
7

7
1263
genus

7
46228
species

SRS015663\_contig\_number\_37462SRS015890\_contig\_number\_17824SRS018984\_contig\_number\_13827SRS048060\_contig\_number\_contig-100\_10642.50671SRS051031\_contig\_number\_36264SRS052697\_contig\_number\_38035SRS142503\_contig\_number\_contig-100\_20193.20193
