# Supplementary material for: MetaRibo-Seq measures translation in microbiomes
Source: Nat Commun. 2020 Jun 29;11:3268. doi: 10.1038/s41467-020-17081-z (PMC7324362; doi:10.1038/s41467-020-17081-z)
Supplement: Supplementary file 10 — Supplementary Data 7 [file 41467_2020_17081_MOESM10_ESM.zip › File2/Confidence_VeryHigh_Taxonomy/349338_out.krona.html]

Javascript must be enabled to view this page.

members
magnitude
magnitudeUnassigned
count
unassigned
taxon
rank

349338\_out

11

superkingdom
11
2

phylum
11
1239


SRS045826\_contig\_number\_11219SRS077294\_contig\_number\_18098SRS143523\_contig\_number\_13266SRS143895\_contig\_number\_31845SRS146813\_contig\_number\_contig-100\_3238.144055
1263000
species
5

class
6
186801

186802
order
1
6

SRS146812\_contig\_number\_contig-100\_4789.271364


SRS018836\_contig\_number\_26820SRS144506\_contig\_number\_55217
species
2
1950912

species
2
1898207

SRS019068\_contig\_number\_93787SRS1041140\_contig\_number\_18114

541000
family
1

1
genus
216851

species
1
853

SRS098073\_contig\_number\_13311
