# Supplementary material for: MetaRibo-Seq measures translation in microbiomes
Source: Nat Commun. 2020 Jun 29;11:3268. doi: 10.1038/s41467-020-17081-z (PMC7324362; doi:10.1038/s41467-020-17081-z)
Supplement: Supplementary file 10 — Supplementary Data 7 [file 41467_2020_17081_MOESM10_ESM.zip › File2/Confidence_VeryHigh_Taxonomy/320435_out.krona.html]

Javascript must be enabled to view this page.

members
magnitude
magnitudeUnassigned
count
unassigned
taxon
rank

320435\_out

17


SRS077861\_contig\_number\_contig-100\_18153.42776
1

superkingdom
16
2

976
phylum
16

200643
16
class

171549
order
16

171552
15
family

838
genus
14

59823
1

SRS012989\_contig\_number\_5066
species

165179
species

SRS015590\_contig\_number\_13960SRS015794\_contig\_number\_9290SRS023526\_contig\_number\_38892SRS043667\_contig\_number\_1238SRS049959\_contig\_number\_12620SRS077641\_contig\_number\_18288SRS077849\_contig\_number\_30770SRS1041134\_contig\_number\_contig-100\_2429.87274SRS104327\_contig\_number\_1770SRS143876\_contig\_number\_13321SRS144537\_contig\_number\_contig-100\_1749.268245SRS148253\_contig\_number\_12693
12

1262930
species

SRS019068\_contig\_number\_26062
1

1926672
genus
1

1

SRS015431\_contig\_number\_44971
species
1852370

family
1
2005525

genus
1
375288

823
1

SRS012969\_contig\_number\_38897
species
