# Supplementary material for: MetaRibo-Seq measures translation in microbiomes
Source: Nat Commun. 2020 Jun 29;11:3268. doi: 10.1038/s41467-020-17081-z (PMC7324362; doi:10.1038/s41467-020-17081-z)
Supplement: Supplementary file 10 — Supplementary Data 7 [file 41467_2020_17081_MOESM10_ESM.zip › File2/Confidence_VeryHigh_Taxonomy/398244_out.krona.html]

Javascript must be enabled to view this page.

members
magnitude
magnitudeUnassigned
count
unassigned
taxon
rank

398244\_out

5

5
2
superkingdom

1239
4
phylum

4
186801
class

order
4
186802

family
4
541000


SRS021948\_contig\_number\_24263SRS049959\_contig\_number\_49223SRS144537\_contig\_number\_30716SRS150029\_contig\_number\_19147
1898205
4
species

1224
1
phylum

class
1
28211

204455
1
order

31989
1
family

1
302485
genus

SRS098571\_contig\_number\_61300
