# Supplementary material for: MetaRibo-Seq measures translation in microbiomes
Source: Nat Commun. 2020 Jun 29;11:3268. doi: 10.1038/s41467-020-17081-z (PMC7324362; doi:10.1038/s41467-020-17081-z)
Supplement: Supplementary file 10 — Supplementary Data 7 [file 41467_2020_17081_MOESM10_ESM.zip › File2/Confidence_VeryHigh_Taxonomy/5409_out.krona.html]

Javascript must be enabled to view this page.

members
magnitude
magnitudeUnassigned
count
unassigned
taxon
rank

5409\_out

13

superkingdom
13
2

phylum
13
1239

13
class
186801

186802
13
order

family
13
186806

genus
13
7

SRS017103\_contig\_number\_contig-100\_20182.20183SRS018427\_contig\_number\_15841SRS023715\_contig\_number\_contig-100\_16071.16072SRS049402\_contig\_number\_contig-100\_26323.26323SRS051031\_contig\_number\_contig-100\_26830.26830SRS149879\_contig\_number\_contig-100\_47278.47279SRS149879\_contig\_number\_contig-100\_12991.62906
1730

4
species

SRS015431\_contig\_number\_25213SRS143342\_contig\_number\_18631SRS144183\_contig\_number\_26700SRS148159\_contig\_number\_44136
39488

165185
2
species

SRS077502\_contig\_number\_6445SRS1041091\_contig\_number\_9287
