# Supplementary material for: MetaRibo-Seq measures translation in microbiomes
Source: Nat Commun. 2020 Jun 29;11:3268. doi: 10.1038/s41467-020-17081-z (PMC7324362; doi:10.1038/s41467-020-17081-z)
Supplement: Supplementary file 10 — Supplementary Data 7 [file 41467_2020_17081_MOESM10_ESM.zip › File2/Confidence_VeryHigh_Taxonomy/83735_out.krona.html]

Javascript must be enabled to view this page.

members
magnitude
magnitudeUnassigned
count
unassigned
taxon
rank

83735\_out

8

8
superkingdom
2

phylum
8
1239

186801
8
class

186802
order
8

186806
family
5

1730
genus
5

39490

SRS012273\_contig\_number\_contig-100\_36180.111459SRS015578\_contig\_number\_10627SRS077552\_contig\_number\_3072SRS143523\_contig\_number\_contig-100\_792.793SRS143991\_contig\_number\_contig-100\_8563.100801
5
species

3
family
186803

841
2
genus

1
species
1262949

SRS014923\_contig\_number\_17698

1
species

SRS020233\_contig\_number\_47234
2292368

1432051
genus
1


SRS097889\_contig\_number\_contig-100\_24735.69481
1720294
1
species
