# Supplementary material for: MetaRibo-Seq measures translation in microbiomes
Source: Nat Commun. 2020 Jun 29;11:3268. doi: 10.1038/s41467-020-17081-z (PMC7324362; doi:10.1038/s41467-020-17081-z)
Supplement: Supplementary file 10 — Supplementary Data 7 [file 41467_2020_17081_MOESM10_ESM.zip › File2/Confidence_VeryHigh_Taxonomy/342275_out.krona.html]

Javascript must be enabled to view this page.

members
magnitude
magnitudeUnassigned
count
unassigned
taxon
rank

342275\_out

10

2
9
superkingdom

1239
9
phylum

class
8
186801


SRS014923\_contig\_number\_22025
1
8
186802
order

186803
6
family

genus
1
841

species

SRS017191\_contig\_number\_13672
1262940
1

572511
3
genus


SRS050752\_contig\_number\_contig-100\_2483.154777SRS147377\_contig\_number\_9325SRS148159\_contig\_number\_56471
species
3
1322

genus
830
2

1520808
2

SRS050925\_contig\_number\_22072SRS076929\_contig\_number\_26856
species

family
1
31979

genus
1485
1

species

SRS077024\_contig\_number\_4117
1
59620

91061
1
class

1
186826
order

1
1300
family

genus
1301
1

species

SRS104165\_contig\_number\_9396
1
1715098

1

SRS063985\_contig\_number\_contig-100\_3361.43181
