# Supplementary material for: MetaRibo-Seq measures translation in microbiomes
Source: Nat Commun. 2020 Jun 29;11:3268. doi: 10.1038/s41467-020-17081-z (PMC7324362; doi:10.1038/s41467-020-17081-z)
Supplement: Supplementary file 11 — Reporting Summary [file 41467_2020_17081_MOESM11_ESM.pdf]

nature research

Corresponding author(s): Ami Bhatt

Reporting Summary

Nature Research wishes to improve the reproducibility of the work that we publish. This form provides structure for consistency and transparency in reporting. For further information on Nature Research policies, see Authors & Referees and the Editorial Policy Checklist.

Statistical parameters

When statistical analyses are reported, confirm that the following items are present in the relevant location (e.g. figure legend, table legend, main text, or Methods section).

n/a	Confirmed	
☐	☒	The exact sample size (n) for each experimental group/condition, given as a discrete number and unit of measurement
☐	☒	An indication of whether measurements were taken from distinct samples or whether the same sample was measured repeatedly
☐	☒	The statistical test(s) used AND whether they are one- or two-sided<br>*Only common tests should be described solely by name; describe more complex techniques in the Methods section.*
☐	☒	A description of all covariates tested
☐	☒	A description of any assumptions or corrections, such as tests of normality and adjustment for multiple comparisons
☐	☒	A full description of the statistics including central tendency (e.g. means) or other basic estimates (e.g. regression coefficient) AND variation (e.g. standard deviation) or associated estimates of uncertainty (e.g. confidence intervals)
☐	☒	For null hypothesis testing, the test statistic (e.g. F, t, r) with confidence intervals, effect sizes, degrees of freedom and P value noted<br>*Give P values as exact values whenever suitable.*
☒	☐	For Bayesian analysis, information on the choice of priors and Markov chain Monte Carlo settings
☒	☐	For hierarchical and complex designs, identification of the appropriate level for tests and full reporting of outcomes
☐	☒	Estimates of effect sizes (e.g. Cohen's d, Pearson's r), indicating how they were calculated
☒	☐	Clearly defined error bars<br>*State explicitly what error bars represent (e.g. SD, SE, CI)*

Our web collection on statistics for biologists may be useful.

Software and code

Policy information about availability of computer code

Data collection	no software was used.
Data analysis	trim galore version 0.4.0 using cutadapt 1.8.1, bowtie version 1.1.1, Prokka, version 1.1.2, bedtools multicov, R packages: (RiboSeqR, Hmisc, DESeq2, cocor, gplots, ggplot2), One Codex database, metaSPAdes 3.7.0, Blast2GO, CD-hit, RNAcode version 3, David Functional Annotation, MicrobiomeAnalyst, KEGG database, UniProt database, nr database, Byonic proteomics search pipeline v 2.10.5

For manuscripts utilizing custom algorithms or software that are central to the research but not yet described in published literature, software must be made available to editors/reviewers upon request. We strongly encourage code deposition in a community repository (e.g. GitHub). See the Nature Research guidelines for submitting code & software for further information.

Data

Policy information about availability of data

All manuscripts must include a data availability statement. This statement should provide the following information, where applicable:

- Accession codes, unique identifiers, or web links for publicly available datasets
- A list of figures that have associated raw data
- A description of any restrictions on data availability

PRJNA510123: Deposition of raw reads (fastq) for all sequencing technologies.

Field-specific reporting

Please select the best fit for your research. If you are not sure, read the appropriate sections before making your selection.

[x] Life sciences [ ] Behavioural & social sciences [ ] Ecological, evolutionary & environmental sciences

For a reference copy of the document with all sections, see nature.com/authors/policies/ReportingSummary-flat.pdf

Life sciences study design

All studies must disclose on these points even when the disclosure is negative.

Sample size	Multi-omics were performed on 5 samples all as different representatives of various health statuses. Comparisons were not made between samples; rather comparisons were made across technologies independently for each of the 5 samples, all with their own unique de novo generated reference. Additionally, multi'omics was performed on two mock communities.
Data exclusions	No intentional data exclusion occurred. Given limitations in de novo assembly, however, genes that could not be assembled would not be included in downstream analyses.
Replication	All experiments sequencing small RNAs were performed in duplicate.
Randomization	n/a - no comparisons between groups occured. For a given sample, RNA sequencing technologies were performed in the same batch.
Blinding	n/a - no comparisons between groups occured.

Reporting for specific materials, systems and methods

Materials & experimental systems

n/a	Involved in the study	
[x]	[ ]	Unique biological materials
[x]	[ ]	Antibodies
[x]	[ ]	Eukaryotic cell lines
[x]	[ ]	Palaeontology
[x]	[ ]	Animals and other organisms
[ ]	[x]	Human research participants

Methods

n/a	Involved in the study	
[x]	[ ]	ChIP-seq
[x]	[ ]	Flow cytometry
[x]	[ ]	MRI-based neuroimaging

Human research participants

Policy information about studies involving human research participants

Population characteristics	The goal was to test our method's robustness using a large variety of samples types. The following includes information of fecal sample sources. Sample A is from a healthy individual. Sample B is from a patient with a hematological disorder who is undergoing treatment. Sample C is from a patient with a solid malignancy who is undergoing treatment. Sample D is from a patient with Alzheimer's disease. We also perform MetaRibo-Seq on a low diversity fecal sample from a patient with a hematological disorder who is undergoing antibiotic treatment with metronidazole – Sample E
Recruitment	All subjects were recruited at Stanford University as a part of one of three IRB-approved protocols for tissue biobanking and clinical metadata collection (PIs: Dr. Ami Bhatt, Dr. Victor Henderson, Dr. David Miklos). Informed consent was obtained.
